# Gene Bionetwork Analysis of Ovarian Primordial Follicle Development

**DOI:** 10.1371/journal.pone.0011637

**Published:** 2010-07-16

**Authors:** Eric E. Nilsson, Marina I. Savenkova, Ryan Schindler, Bin Zhang, Eric E. Schadt, Michael K. Skinner

**Affiliations:** 1 Center for Reproductive Biology, School of Molecular Biosciences, Washington State University, Pullman, Washington, United States of America; 2 Sage Bionetworks, Seattle, Washington, United States of America; McGill University, Canada

## Abstract

Ovarian primordial follicles are critical for female reproduction and comprise a finite pool of gametes arrested in development. A systems biology approach was used to identify regulatory gene networks essential for primordial follicle development. Transcriptional responses to eight different growth factors known to influence primordial follicles were used to construct a bionetwork of regulatory genes involved in rat primordial follicle development. Over 1,500 genes were found to be regulated by the various growth factors and a network analysis identified critical gene modules involved in a number of signaling pathways and cellular processes. A set of 55 genes was identified as potential critical regulators of these gene modules, and a sub-network associated with development was determined. Within the network two previously identified regulatory genes were confirmed (i.e., Pdgfa and Fgfr2) and a new factor was identified, connective tissue growth factor (CTGF). CTGF was tested in ovarian organ cultures and found to stimulate primordial follicle development. Therefore, the relevant gene network associated with primordial follicle development was validated and the critical genes and pathways involved in this process were identified. This is one of the first applications of network analysis to a normal developmental process. These observations provide insights into potential therapeutic targets for preventing ovarian disease and promoting female reproduction.

## Introduction

An emerging concern in the field of biomedical research is that the common reductionist approach to studying biological processes may not be adequate to fully understand the complex interplay of cellular signaling, gene expression, and other complex molecular processes that occur within a tissue or organ. Examples of reductionist studies that have driven much of our understanding of biological processes associated with complex phenotypes like disease include the knockout mouse experiments and *in vitro* cytokine treatment to assess the effects of gene-specific perturbations on cell or tissue biology. Results from these types of studies provide information on candidate regulatory factors, but typically do not elucidate the network of factors or processes required for a normal developmental biology or pathobiology. A holistic, systems biology, approach to studying normal developmental processes can be a powerful tool that is complementary to the more reductionist experiments. In the spirit of a systems-based approach to development, the current study was designed to identify gene networks involved in ovarian primordial follicle development and to characterize critical regulatory factors involved in this development process.

In mammals, all the oocytes (eggs) that will be used over a female's lifetime are present in the ovary at birth in a finite pool. These oocytes are arrested in prophase of the first meiotic division and are each surrounded by flattened pre-granulosa cells to form a structure called a primordial follicle [1]. During the reproductive lifespan of a female, follicles gradually leave the arrested pool to undergo a primordial to primary follicle transition. A follicle undergoing follicle transition has an increase in oocyte diameter and the associated granulosa cells proliferate and change from a flattened to cuboidal in shape. Once primordial to primary follicle transition has occurred the follicle either continues to develop to the point of ovulation or undergoes atresia [1], [2], [3], [4]. Previously cell-to-cell communication with extra-cellular growth factors has been shown to regulate the initiation of primordial follicle development. These studies have primarily used a reductionist approach to test candidate growth factors one at a time for their ability to affect follicle transition. A number of paracrine growth factors have been identified as having a role in early follicle development (reviews [4], [5]).

To move beyond examining single gene effects on this development process, gene network analysis can be employed to identify groups (e.g. modules) of genes whose expression is regulated in a coordinated manner (gene network) [6], [7], [8]. In this type of analysis, a biological system is surveyed in the context of disease (or other interesting phenotypes) with microarrays multiple times with and without perturbations that cause the system to change. A novel bioinformatics analysis is used to identify modules of genes associated with biological systems (bionetwork). The great majority of network analyses have focused on disease states and been used to better understand the systems biology of disease processes and identify potential therapeutic targets [9], [10], [11], [12], [13], [14], [15]. The current study was designed to determine if network analysis can be applied to study a normal development process.

The current study used whole rat ovaries cultured *in vitro* in a manner that allowed primordial to primary follicle transition. The ovaries were treated with one of eight different growth factors previously shown to regulate primordial follicle transition in comparison to untreated control cultures. The mRNA was isolated from the ovaries and used for microarray transcriptome analysis to globally survey gene expression under these different treatment conditions. The effects of each growth factor on gene expression were analyzed to determine similarities and differences in gene expression between the different growth factor treatments. Those genes whose mRNA expression changed with any treatment were subjected to network analysis to identify pathways and genes with a high degree of connectivity between other genes and pathways. From the networks constructed from these data we identified a list of critical modules of regulated genes forming gene sub-networks that were used to identify regulatory genes involved in primordial follicle development. Not only were previously identified regulatory factors/genes associated with this process identified from this network analysis, but a number of putative regulators of follicle transition not previously associated with this process were also determined. One of the new candidate genes, connective tissue growth factor (Ctgf) [16], was tested experimentally and found to promote primordial to primary follicle transition. Observations demonstrate the utility of this network analysis to be used as a systems biology approach to study normal developmental processes in complex systems.

## Results

### Primordial Follicle Transcriptome Analysis

**Figure 1:**
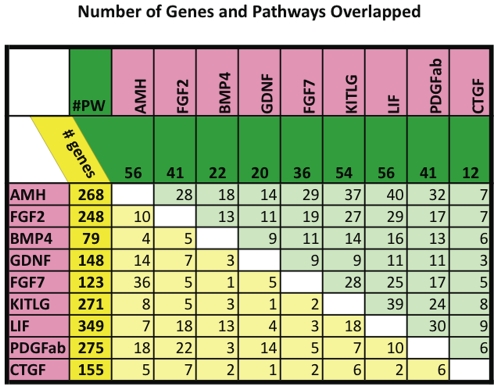
Number of genes and pathways overlapped between signature (growth factor treatment group) lists. Total number of differentially expressed genes for each growth factor is shown in dark yellow column, number of genes overlapped between each pair of signature lists – in light yellow columns. Total number of KEGG pathways affected by each growth factor is shown in dark green row; number of KEGG pathways overlapped for each pair of growth factors is shown in light green row. CTGF analysis separate from the network analysis.

A number of regulatory factors have been shown to affect primordial to primary follicle transition, including Amh [17], [18], Fgf2 [19], [20], [21], Bmp4 [22], [23], Gdnf [24], Fgf7/KGF [25], Kitlg [19], [26], [27], Lif [28] and Pdgfa [29]. In order to determine the underlying gene networks and processes involved in primordial follicle development, microarray analysis was performed on RNA from whole rat ovaries treated for two days in vitro with each of the above listed growth factors independently. There were three independent RNA samples of pooled ovaries for each growth factor treatment (except for GDNF, which had only two sample replicates), and corresponding control samples for a total of 38 RNA samples. These were evaluated using 38 Affymetrix Rat Gene 1.0 ST microarrays. The array data were analyzed together using normalization and pre-processing described in the Methods. Each growth factor treatment resulted in 79 to 349 genes with altered expression compared to controls (Figure 1). The lists of the genes affected by each treatment are presented in Table S1. There were relatively few genes with altered expression in common between the different treatments (Figure 1). Less than 10% of the genes changed by any one growth factor treatment were found to be changed in any other treatment. The exception was Fgf7/KGF, which had a more than 30% overlap of altered genes with Amh. There were no individual genes that changed expression levels in response to more than three of the eight original treatments (Table S1).

The complete list of genes whose expression levels changed with any of the treatments was compared to curated lists of genes from the KEGG database to identify processes that may be important for primordial follicle development. Automated unbiased matching of lists of affected genes to KEGG pathways was performed with Pathway Express (Intelligent Systems and Bioinformatics Laboratory; http://vortex.cs.wayne.edu/ontoexpress/). Pathways heavily impacted by genes whose expression altered in response to the growth factor treatments (Table 1) included pathways involved in cell surface and extracellular matrix regulation (cell adhesion molecules, adherens junction, focal adhesion, tight junction, gap junction, regulation of actin cytoskeleton), known signaling pathways (MAPK, notch, B-cell receptor, adipocytokine, toll-like receptor, ErbB, GnRH, Wnt, hedgehog, VEGF, Jak-STAT, TGF-beta, p53, insulin, PPAR), the complement cascade, axon guidance, glycan structure biosynthesis and pathways listing cell communication ligand-receptor interactions (cytokine-cytokine receptor, neuroactive ligand-receptor, ECM-receptor). There was a high degree of overlap of affected pathways between different growth factor treatments (Figure 1). For the list of pathways containing altered genes, from 70% to 82% of those pathways are shared with at least one other treatment. Application of the hypergeometrical Fisher Exact Test to assess whether the number of overlapped pathways was significantly greater than expected by chance, revealed that the majority were statistically significant. The pathways containing altered genes from several growth factor treatments are presented in Table 1. Although few altered genes were found to overlap between different treatments (Figure 1), each growth factor treatment influenced similar pathways, Table 1 and Table S1. Therefore, each growth factor affects similar pathways via different genes.

**Table 1:** Number of Regulated Genes in Pathways.

Pathway Name	Total List (1540)		Module Lists																Signature Lists								
	Total Overlap Number	Impact Factor	Short/Connected List	turquoise	blue	brown	yellow	green	red	black	pink	magenta	purple	green-yellow	tan	salmon	cyan	grey	AMH	bFGF	BMP4	GDNF	KGF	KL	LIF	PDGF	CTGF
Number of genes in the list			55	194	182	158	150	139	112	99	85	68	45	32	29	28	22	157	268	248	79	148	123	271	349	275	155
Neuroactive ligand-receptor interaction	**22**	**1.23**	1	2		5	2	1	3			1	1	2	1		1	3	4	5		1	4	5	4	6	3
Focal adhesion	**19**	**3.62**	3	7	1	7	1		1			1	1						3	3			4	8	3	1	
MAPK signaling pathway	**19**	**3.00**	6	7	1	3	4	1						1			1	1	6		4		5	4	6		
Cell adhesion molecules (CAMs)	**15**	**107**	1	1	3	2	1		1			3		1	1			2	6	2	3	2		3	2	2	1
Axon guidance	**15**	**6.34**	1	4	1	2	4	1						1	1				6		1	3	2	3	3	3	
Cytokine-cytokine receptor interaction	**15**	**2.24**	1	3	3	4	3			1								1	2	1	4		1	6	5		1
Calcium signaling pathway	**13**	**1.72**	1	3		5	1		2					1			1		3	2		1	2	3	4	1	
Regulation of actin cytoskeleton	**12**	**2.18**	2	5	1	4	2												4	2			5	4			
Cell Communication	**11**	**4.70**		2		4	3		1			1							3	1	2		1	4	1	1	
Jak-STAT signaling pathway	**11**	**1.88**		3	1	3	1			2				1					1	1			1	4	4		1
Adipocytokine signaling pathway	**10**	**2.94**		2			2	2			1		1	1		1			1	1	2				7	1	1
ECM-receptor interaction	**9**	**4.23**	2	2		4	2		1										1	2	1			5	2		
Complement and coagulation cascades	**9**	**3.63**		1	1	2	4							1						2	4			1	4		1
Apoptosis	**9**	**2.94**		1		2	2	1		2	1								2	1	2			2	5		
Toll-like receptor signaling pathway	**9**	**2.92**		3	1	1		1	1					1				1	2	1	1		1	2	1	1	
Leukocyte transendothelial migration	**9**	**2.74**		1	2	3	1					1						1	3	1				5	1		
Gap junction	**9**	**2.73**	1	2	1	2	2	1						1					2				1	3	1	3	
Insulin signaling pathway	**9**	**1.35**			1	1		1	2			1	2			1				2	1		1	4		2	1
Adherens junction	**8**	**33.1**		3		3						1						1	2					3	4	1	
Glycan structures - biosynthesis 2	**8**	**3.83**	1	2	2			1	1							1		1	1	3		1	3		1		
T cell receptor signaling pathway	**8**	**2.71**		2	1	1	1						1					2	3			1	2	2	1		
Tight junction	**7**	**3.28**		1		1	1	1	1			1						1		1				2	4		
GnRH signaling pathway	**6**	**2.84**	1	2		2											1	1	1				2	2	1		
TGF-beta signaling pathway	**6**	**1.40**		1	2						1	2							1		2	1	2		1	1	1
PPAR signaling pathway	**6**	**1.24**	1	1		1	1	1								1		1		1		1		1	2		
Antigen processing and presentation	**5**	**36.8**			1			1				2						1	1	1	1	1			1		
Melanogenesis	**5**	**3.34**	1	2		1	1				1								1	1				1	2	1	
B cell receptor signaling pathway	**5**	**2.95**		2	1	2													2	1		1		2			
Wnt signaling pathway	**5**	**2.84**		3			1								1				2	1				1	3		
Hematopoietic cell lineage	**5**	**1.28**		1	1		2											1	1	2	2		1		1		
ErbB signaling pathway	**4**	**2.89**				2	1											1	1				1	3			
Hedgehog signaling pathway	**4**	**2.38**		2		2													1	2					1	1	
VEGF signaling pathway	**4**	**2.17**		1		2							1						1					1	1	1	
p53 signaling pathway	**4**	**1.37**			1		1			1				1							1			1	2	1	
Glycan structures - biosynthesis 1	**4**	**0.85**		1	1				1										1	1	2	1	1				
Renin-angiotensin system	**3**	**3.21**															1	2		1				2			
Notch signaling pathway	**3**	**2.95**				1	1	1											1					1	1		1
mTOR signaling pathway	**3**	**2.82**				1						1	1							1				1	1	1	
Basal transcription factors	**3**	**2.03**							1		1					1				1		1	1				
Fc epsilon RI signaling pathway	**3**	**1.87**		1		1							1						1					2			
Nucleotide excision repair	**3**	**1.49**														1		2	2	1			1			1	
Cell cycle	**3**	**1.13**	1	1					1					1							1		1	1	1		
Ubiquitin mediated proteolysis	**3**	**0.98**	1	1		1					1									1			1	1			
Phosphatidylinositol signaling system	**2**	**28.0**						1										1							2		

Number of regulated genes in affected KEGG pathways for each of modules and signature (Growth Factor Treatment) lists.

Pathway enrichment analysis was performed using KEGG database, current for July 2009, for which gene symbols were used as input data. From 1,540 probe sets, 1,137 were identified as annotated genes using Affymetrix RaGene-1_0-st-v1 Transcript Cluster Annotations, release 28 (March, 2009). Top 44 pathways shown except for disease pathways (not shown). No KEGG pathways were affected by genes from light-cyan or midnight-blue modules.

### Bionetwork Analysis

**Figure 2:**
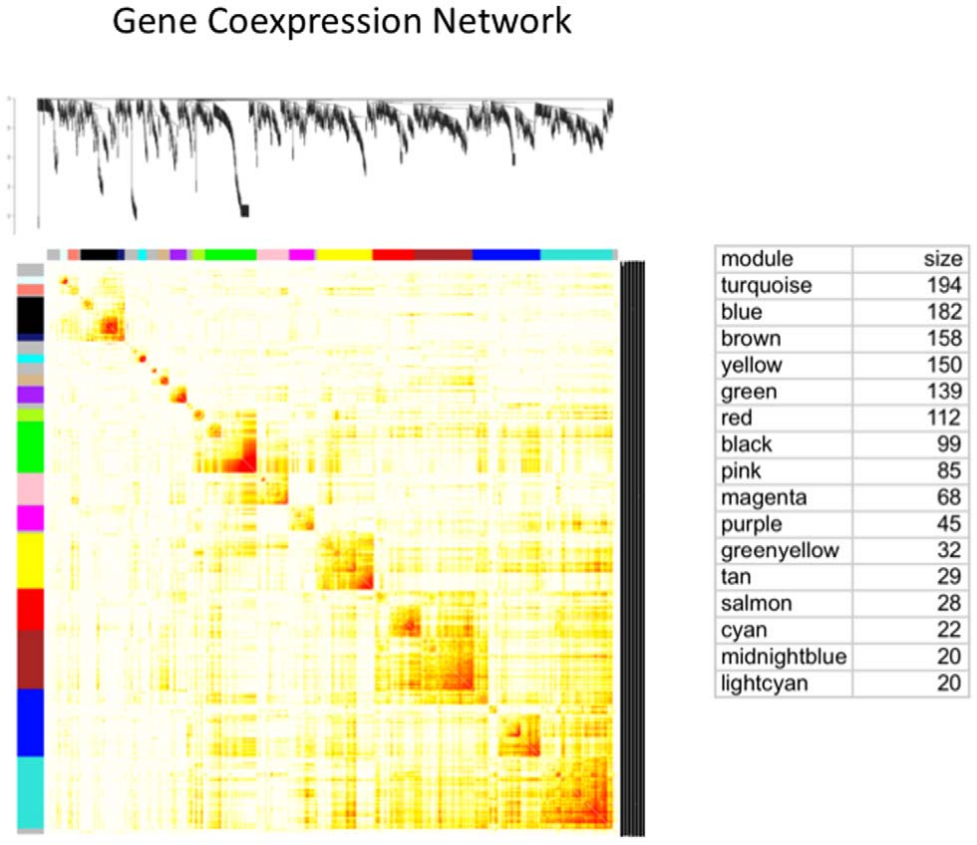
The ovary gene co-expression network and corresponding gene modules. A topological overlap matrix of the gene co-expression network consisting of the 1540 genes regulated by the various growth factors. Genes in the rows and columns are sorted by an agglomerative hierarchical clustering algorithm (see Methods). The different shades of color signify the strength of the connections between the nodes (from white signifying not significantly correlated to red signifying highly significantly correlated). The hierarchical clustering (top) and the topological overlap matrix strongly indicate highly interconnected subsets of genes (modules). Modules identified are colored along both column and row and are boxed. The number of genes in each module is listed as size of module.

The complete list of genes whose expression levels changed with any growth factor treatment was subjected to a network analysis as described in Methods. Potential batch effects for culture date, RNA processing data and microarray performance date were corrected during the analysis, with no major effect on the analysis. The data were fit using a robust linear regression model (rim function from R statistical package), and then the residuals with respect to the model fit were carried forward in all subsequent analysis. The network analysis scores each gene according to how well, under different treatments, its changes in gene expression are correlated with the changes in expression of every other gene. This gives a connectivity score for each gene. High connectivity scores indicate that expression of this gene changes in concert with that of many other genes. In addition, the network analysis identifies gene modules in which the member genes have similar changes in expression in response to the various growth factor treatments. Gene modules are functional components of the network that are often associated with specific biological processes. To identify modules comprised of highly interconnected expression traits within the co-expression network, we examined the topological overlap matrix [30] associated with this network. The topological overlap between two genes not only reflects their more proximal interactions (e.g., two genes physically interacting or having correlated expression values), but also reflects the higher order interactions that these two genes may have with other genes in the network. Figure 2 depicts a hierarchically clustered topological overlap map in which the most highly interconnected modules in the network are readily identified. The specific details of the gene co-expression network analysis (Figure 2) are presented in the Methods section. To identify gene modules (sub-networks) formally from the topological overlap map, we employed a previously described dynamic cut-tree algorithm with near optimal performance on complicated dendrograms [31] (see Methods for details). Figure 2 shows the topological overlap map of the co-expression network with gene modules color-coded for the 16 modules identified. The membership of each module can be found in Table S1. The sixteen modules contained 1,383 genes with the remaining 157 genes (colored as gray) not failing into any module.

The pathways containing genes whose expression changed with growth factor treatment were compared to the genes from each module that were associated with specific pathways (Table 1 and Table S1 for full list). For most pathways genes from several network modules were present. However, several pathways were associated with selected modules. For example, out of 19 altered genes present in the focal adhesion pathway, seven were from the turquoise module and seven from the brown. Similarly, of the five altered genes in the Wnt signaling pathway three were from the turquoise module. For the fifteen changed genes in the cell adhesion molecule pathways three were from blue and three from magenta modules. Those genes whose expression changed with specific growth factor treatments were cross-matched with the genes assigned to each network module to determine if specific modules were heavily influenced by particular growth factors. Interestingly, each module was biased toward having many genes in common with selected growth factors (Table S1). In contrast, some growth factor treatments induced changes in genes that were distributed among several different modules.

In order to identify genes that could be key regulators of primordial follicle development, a shorter list of candidate genes was generated from the results of the network analysis. Six modules were chosen for having the highest numbers of known regulatory genes and pathways (yellow, turquoise, blue, brown, red, purple). The top 10% of most connected transcripts in each of the six modules were identified as potential important regulators [13], [15], except for the blue module for which the top 20% were chosen since so many of that module's most highly connected transcripts were not annotated as genes. The compiled list included 55 transcripts annotated as genes (Table 2), and these genes were subjected to more intensive investigation.

**Table 2:** List of 55 Candidate Regulatory Genes.

Function	GeneSymbol	GeneBank	Probeset	k.in*	Module	Regulation by Growth Factor	GeneTitle
**Apoptosis**	Bcl2l10	NM_053733	10911690	10.0	red	KL-dwn	BCL2-like 10
**Cell Cycle**	Cdkn2c	NM_131902	10878705	7.6	red	KL-dwn	CDK4 inhibitor 2C
**ECM**	Cdh3	NC_005118	10807525	28.0	turq	AMH-dwn	cadherin 3, type 1, P-cadherin
	Col11a1	AJ005396	10818502	20.6	brown	KL-dwn	collagen, type XI, alpha 1
	Krt19	NM_199498	10747262	25.2	turq	AMH-dwn	keratin 19
	Lama5	NC_005102	10852270	30.9	turq	AMH-dwn	laminin, alpha 5
**Development**	Bnc2	NM_001106666	10877880	26.5	turq	AMH-dwn	basonuclin 2
	Emx2	NM_001109169	10716454	27.5	turq	KGF-dwn	empty spiracles homeobox 2
	Usmg5	NM_133544	10730633	13.5	blue	AMH, GDNF-dwn	muscle growth 5 homolog (mouse)
**Epigenetics**	Dnmt1	NM_053354	10915437	22.9	brown	KL-dwn	DNA (cytosine-5-)-methyltransferase 1
**Golgi**	B4galt6	NM_031740	10803394	11.7	blue	AMH-dwn, KGF-dwn	galactosyltransferase, polypeptide 6
**Growth Factors**	Ctgf	NM_022266	10717233	8.2	blue	KGF-up, LIF-dwn	connective tissue growth factor
	Il16	NM_001105749	10723351	31.9	turq	AMH-dwn, KGF-dwn	interleukin 16
	Pdgfa	NM_012801	10757129	27.0	turq	AMH-dwn, KGF-dwn	platelet-derived growth factor
**Immune**	LOC287167	NM_001013853	10741765	21.6	brown	KL-dwn	globin, alpha
**Metabolism**	Cacna2d3	NM_175595	10789819	19.9	brown	KL-dwn	calcium channel
	Hbq1	XM_001061675	10741761	24.0	brown	KL-dwn	hemoglobin, theta 1
	Hhatl	NM_001106868	10914424	15.7	yellow	PDGF-up	hedgehog acyltransferase-like
	Hmgcs2	NM_173094	10817759	19.5	brown	KL-dwn	Coenzyme A synthase 2
	Hsd11b1	NM_017080	10770795	17.0	yellow	BMP4-dwn	hydroxysteroid 11-beta dehydro
	Kirrel	NM_207606	10824123	14.1	yellow	AMH-dwn	kin of IRRE like (Drosophila)
	Plod2	NM_175869	10912255	18.3	brown	KL-dwn	procollagen lysine, 2-oxoglutarate
	Podxl	NM_138848	10861662	26.6	turq	AMH-dwn, KGF-dwn	podocalyxin-like
	Scn3a	NM_013119	10845809	17.4	brown	KL-dwn	sodium channel, type III, alpha
	Slc4a4	NM_053424	10775997	29.0	turq	AMH-dwn, KGF-dwn	solute carrier family 4
	Slc7a5	NM_017353	10811531	15.6	yellow	PDGF-up	solute carrier family 7
	Slc29a1	NM_031684	10921833	15.4	yellow	PDGF-up	solute carrier family 29
	Eno1	NM_012554	10874152	9.0	purple	PDGF-up	enolase 1, (alpha)
**Receptors**	Axl	NM_031794	10719900	15.4	yellow	BMP4-dwn	Axl receptor tyrosine kinase
	Ednrb	NM_017333	10785724	20.7	brown	KL-dwn	endothelin receptor type B
	Fgfr2	NM_012712	10726172	30.8	turq	AMH-dwn, KGF-dwn	fibroblueast growth factor receptor 2
	Itgb3bp	NM_001013213	10878272	11.0	blue	GDNF-dwn	integrin beta 3 binding protein
	Plxna4a	NM_001107852	10861678	30.9	turq	AMH-dwn, KGF-dwn	plexin A4, A
	Tmem151a	NM_001107570	10727725	26.2	turq	AMH-dwn, KGF-dwn	transmembrane protein 151A
**Signaling**	Nrgn	NM_024140	10916228	16.1	yellow	AMH, KGF-dwn, PDGF-up	neurogranin
	Dusp4	NM_022199	10792035	17.3	yellow	AMH-dwn	dual specificity phosphatase 4
	Dusp6	NM_053883	10895144	16.6	yellow	BMP4-dwn	dual specificity phosphatase 6
	Efna5	NM_053903	10930204	15.0	yellow	AMH, GDNF-dwn	ephrin A5
	Map3k1	NM_053887	10821276	26.8	turq	KGF-dwn	mitogen activated protein kinase
	Pde7b	NM_080894	10717069	17.8	brown	KL-dwn	phosphodiesterase 7B
	Rem1	NM_001025753	10840861	14.5	yellow	PDGF-up	RAS (RAD and GEM)-like
	Shc4	NC_005102	10849423	17.0	yellow	AMH-dwn	SHC family, member 4
	Ubash3b	AC_000076	10916476	16.9	yellow	PDGF-up	ubiquitin associated
**Transcription**	Btg4	NM_001013176	10909937	7.4	red	KL-dwn	B-cell translocation gene 4
	Etv5	NM_001107082	10752034	14.4	yellow	AMH-dwn	ets variant 5
	Fbxo15	NM_001108436	10803025	11.9	red	KL-dwn	F-box protein 15
**Misc. & Unknown**	Depdc2	NM_001107899	10875023	29.5	brown	KL-dwn	DEP domain containing 2
	Fam154a	AC_000073	10877890	11.4	red	KL-dwn	similarity 154, member A
	LOC686725	AC_000076	10915208	25.9	turq	AMH-dwn	hypothetical protein LOC686725
	RGD1306186	BC090317	10881318	9.2	red	KL-dwn	similar to RIKEN cDNA 4930569K13
	RGD1306622	XM_001074493	10728647	32.6	turq	AMH-dwn, KGF-dwn	similar to KIAA0954 protein
	RGD1308023	XR_006437	10850490	17.6	brown	KL-dwn, LIF-dwn	similar to CG5521-PA
	RGD1566021	AC_000086	10800122	19.6	brown	KL-dwn	similar to KIAA1772

Short list of 55 genes that are the most connected genes with known functions in the modules of interest.

Selected from the top 10% most connected genes in each module (except blue module for which considered top 20% as many hubs are not annotated. Abbreviations used: dwn - down-regulated; up - up-regulated; (*)- k in. is connectivity coefficient obtained/calculated in network analysis.

**Figure 3:**
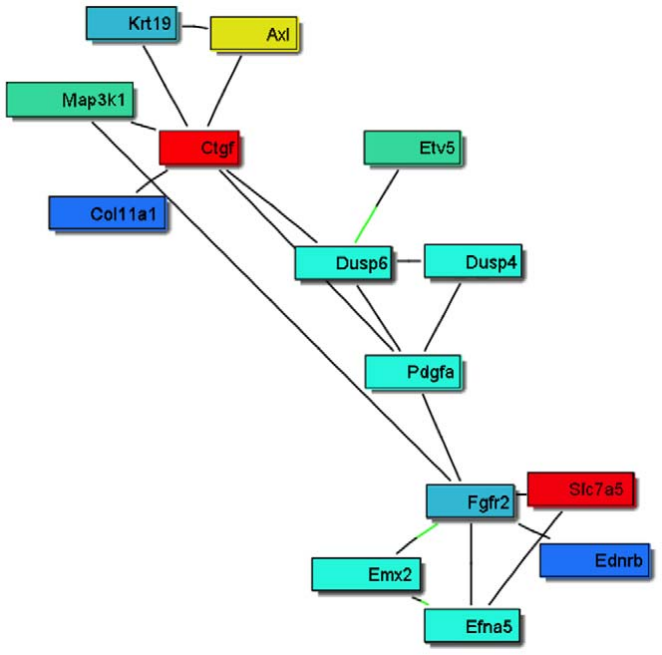
Sub-network connection scheme for the most highly connected 55 candidate genes obtained by global literature analysis. Only 15 connected genes from the list of 55 are shown, while the rest are not connected and not shown. Red and yellow colors represent up-regulated genes, blue and turquoise colors – down-regulated genes.

An automated unbiased analysis of published scientific literature was applied to the lists of differentially expressed genes described above using Genomatix/BiblioSphere software, as described in the Methods. Figure 3 shows a small integrated gene network among the short list of the 55 candidate regulators (Table 2). These relationships in literature raise the possibility that physiological interactions exist between these genes. The gene Ctgf (Connective tissue growth factor) was seen to relate to several other genes with high connectivity, Figure 3. Interestingly, two of the identified genes were previously shown to influence primordial follicle development, Pdgfa [29] and Fgfr2 [19], [20], [21].

The entire set of 1540 transcripts differentially expressed with growth factor treatment was also subjected to analysis using BiblioSphere. Only 632 were recognized by BiblioSphere and 613 were connected. A diagram of literature relationships between these genes is presented in Figure S1. Five major gene clusters were identified as associated with Nfkb1, Vegfa, Gadd45a, Esr1 and Egfr1. This analysis is useful to compare with the expression network analysis, but is biased toward the literature and finding relationships among more heavily studied factors.

### Analysis of CTGF Actions

**Figure 4:**
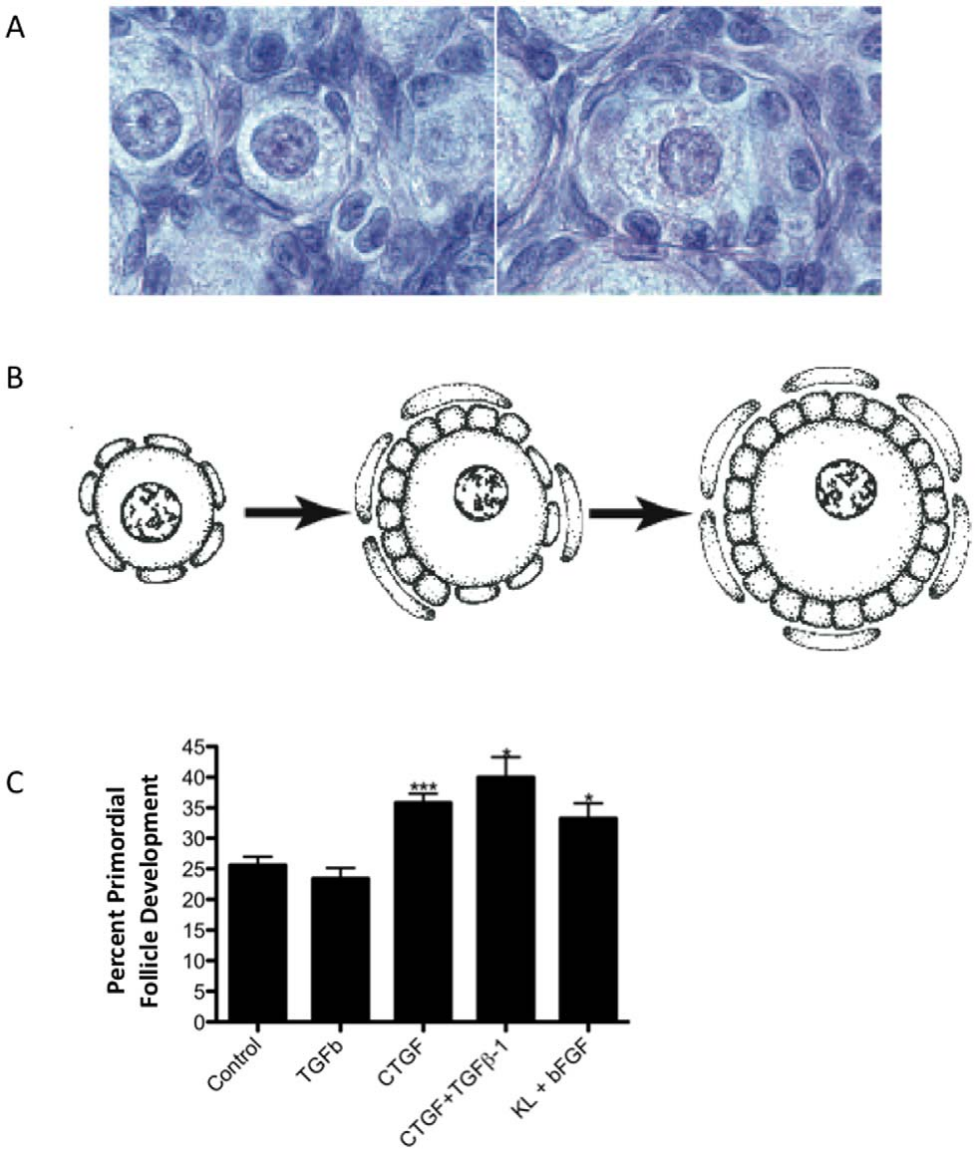
Analysis of the role of CTGF in primordial follicle transition. **A**) Hematoxylin-eosin stained ovary sections showing a representative arrested primordial follicle (left), and a developing primary follicle after having undergone primordial to primary follicle transition (right). **B**) Graphic representation of primordial and primary follicles. **C**) Effect of CTGF on cultured ovaries. Ovaries were cultured for 10 days with the treatments indicated. Ovarian histological analysis determined the percentage of primordial versus developing follicles for each ovary. Bars are the mean percent developing follicles ± SEM. N = 5–7 per treatment from four different replicate experiments. Asterisks indicate a significant (*p<0.05 or ***p<0.01) difference from control by Dunnet's post-hoc test after a significant result of ANOVA.

Critical regulatory candidates for primordial follicle development were selected due to their being differentially expressed in response to treatment with growth factors, having a high connectivity score, and being related in literature to other highly connected genes. For the purpose of the current study, candidate regulatory genes that were also extracellular growth factors were considered. Therefore, CTGF was selected based on all these criteria for further analysis. Experiments were performed to see if CTGF could regulate primordial to primary follicle transition. Ovaries from four-day old rats were treated with 50ng/ml CTGF protein for ten days in an organ culture system as described in the Methods (Figure 4A and 4B). Transforming growth factor beta 1 (TGFB1), which is known to interact with CTGF [32], [33], was also tested. Untreated cultured ovaries were used as a negative control, and ovaries treated with 50ng/ml each of Kit ligand (KITL) and Fibroblast growth factor 2 (FGF2) were used as a positive control. CTGF treatment resulted in a significant (p<0.05) increase in developing follicles compared to untreated controls, as did treatment with the combination of KITL and FGF2 (Figure 4C). TGFB1 had no effect, either alone or in combination with CTGF.

RNA was collected from CTGF and control cultured ovaries as described in the Methods from three replicate experiments. The RNA was used for microarray analysis using the same criteria as for the other growth factors used in the network analysis. One hundred fifty-five transcripts were differentially expressed in CTGF-treated ovaries, Table S1. As was seen for the other growth factors used in the network analysis, there was little overlap of these changed genes with the genes showing changed expression in response to any other growth factor treatment, Figure 1. However, as seen among the other growth factors, there was a high degree of overlap between the pathways impacted by CTGF treatment and treatment with other growth factors (Figure 1). Therefore, a critical regulatory gene predicted from the network analysis was confirmed to regulate primordial follicle development.

## Discussion

A systems biology approach was used to elucidate the changes in gene expression that are important for ovarian primordial to primary follicle transition. A gene network analysis was performed on the ovarian transcriptomes following treatment with 8 different growth factors. The rat ovary was used as a model system to test the utility of this approach in investigating a normal developmental process. This is one of the first applications of network analysis to a normal developmental process. The objective was to identify critical regulatory factors and pathways in primordial follicle development following a bionetwork analysis.

**Figure 5:**
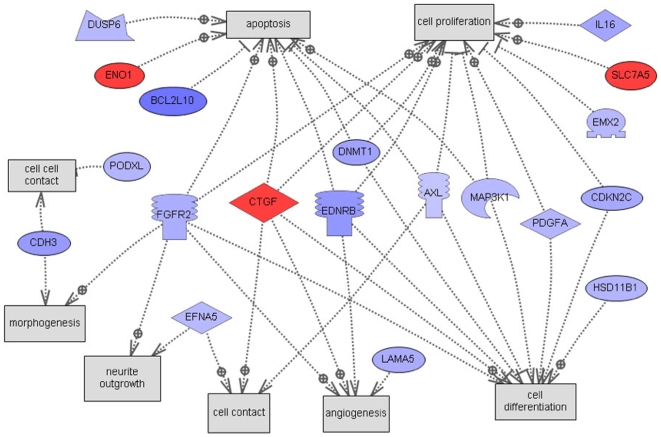
Scheme of direct connections to cellular processes for the 55 candidate regulatory genes obtained by global literature analysis. Only 19 connected genes from the list of 55 are shown, the rest are not connected and not shown. Node shapes code: oval and circle – protein; crescent – protein kinase and kinase; diamond – ligand; irregular polygon – phosphatase; circle/oval on tripod platform – transcription factor; ice cream cone – receptor. Red color represents up-regulated genes, blue color – down-regulated genes, grey rectangles represent cell processes; arrows with plus sign show positive regulation/activation, arrows with minus sign – negative regulation/inhibition.

Microarray analysis determined the alterations in the ovarian transcriptome that occurred in response to treatment of ovaries with AMH, FGF2, BMP4, GDNF, FGF7, KITL, LIF, and PDGFB. All of these have previously been shown to effect follicle transition [17], [18], [19], [20], [21], [22], [23], [24], [25], [26], [27], [28], [29], [34]. All these factors stimulate primordial follicle development except AMH that inhibits follicle development. The presence of both positive and negative factors provides a wider diversity of gene regulation to facilitate the network analysis. As expected the AMH regulated gene set is more distinct from the others. Surprisingly, there were few altered genes in common between all these growth factors and there were no genes that significantly changed in expression level in response to more than three of the eight growth factors. In contrast, the physiological processes impacted by these altered genes were found to have a higher level of overlap. Since a pathway includes groups of genes, it is expected that the overlap of pathways between growth factor treatments will be higher. The overlap of pathways was markedly high (70% to 82%) and statistically different, suggesting pathway associations provide a predicted capacity to identify regulatory factors. Certain pathways were significantly over-represented in the pool of genes with changed expression. This suggests that there are selected physiological pathways that are influenced by all the different growth factors (Figure 5), but that each growth factor affects different genes at different points in these pathways (Table 1). Multiple input points into these physiological pathways could allow for more precise regulation and more effective compensation between the growth factors. Since many growth factors are acting in parallel to regulate these pathways, any one pathway system is robust and maintains function if one growth factor becomes inoperative. Since primordial follicle development is essential for female reproduction, a complex network of regulatory factors influencing different aspects of critical signaling pathways has evolved.

For the eight growth factors evaluated the cellular processes affected in common (Figure 5) included changes in cell contact, morphogenesis, and cell proliferation and differentiation. These are processes that are necessary for the morphological changes that occur with primordial to primary follicle transition. During follicle transition granulosa cells change from squamous to cuboidal and the oocyte starts to grow in diameter (Figure 4B). Unexpectedly, what was also seen as an important affected cellular process was regulation of several key components of the complement and coagulation cascades (Figure S2). These genes are not known for having roles in ovary or follicle development, and merit further investigation.

Gene networks provide a convenient framework for exploring the context within which single genes operate. For gene networks associated with biological systems, the nodes in the network typically represent genes, and edges (links) between any two nodes indicate a relationship between the two corresponding genes. An important end product from the gene co-expression network analysis is a set of gene modules which member genes are more highly correlated with each other than with genes outside a module. It has been demonstrate that these types of modules are enriched for known biological pathways for genes that associate with disease traits and for genes that are linked to common genetic loci [6], [35].

The current study employed a weighted gene co-expression network approach that has been extensively used for uncovering biologically meaningful gene modules [7], [13], [15] to explore novel pathways involved in primordial follicle development. An unsupervised and unbiased approach was used to nominate potential regulatory candidates for these modules based on gene network connectivity. The connectivity score shows how well under different treatments the changes in gene expression for a gene are correlated with the changes in expression for every other gene. In the current study, the gene co-expression network analysis helped select 55 highly connected genes for further functional analysis. An automated literature search of these 55 genes revealed a sub-network relationship among them as presented in Figure 3. This sub-network suggested regulatory roles for Pdgfa and Fgfr2 (the receptor) for Fgf2 and Fgf7 (KGF). PDGF, KGF/FGF7 and FGF2 proteins have previously been shown to regulate primordial to primary follicle transition [29], [34]. Therefore, the bionetwork predicted to be involved in the regulation of primordial follicle development identified two previously known regulatory factors which validated the utility of the network analysis for identifying candidate regulatory genes, consistent with previous network studies [13], [15]. This sub-network also identified connective tissue growth factor (Ctgf) [16], [36] as a putative regulator of primordial follicle development. An ovarian organ culture experiment confirmed that CTGF promotes primordial to primary follicle transition. Therefore, a regulatory factor predicted to be important for primordial follicle development was confirmed to be involved which further validated the bionetwork approach. A microarray analysis of CTGF-treated ovaries showed an altered gene set similar to those of the other growth factors known to regulate follicle transition. These observations validate the network-based systems biology approach to elucidate the regulation of a complex developmental process.

Consideration of the 55 intra-module hub genes from critical regulatory modules revealed a number of signaling and cellular processes were influenced, Figure 5 and Figure S3. In the growth factor/chemokine family Pdgfa and Ctgf were confirmed to be involved. The IL16 identified is currently being investigated as a potential regulatory candidate. The specific genes identified in Table 2 and associated regulatory processes provide potential therapeutic targets to regulate primordial follicle development. The ability to inhibit or stimulate primordial follicle development with a therapeutic treatment has a number of clinical applications. A delay in primordial follicle development and maintenance of the primordial pool could delay the onset of menopause and extend the reproductive life span of a female. In addition, the ability to therapeutically inhibit primordial follicle development would provide a treatment for premature ovarian failure, a disease when the primordial pool is lost early in life causing female infertility. In contrast, the therapeutic stimulation of primordial follicle development could treat forms of female infertility [4]. The induction of primordial follicle development also could promote the loss of the primordial pool and induce female sterility. The bionetwork identified in the current study produced a number of potential therapeutic targets to manipulate primordial follicle development and female reproductive capacity.

The systems biology approach taken with this network analysis of primordial follicle development identified clusters and modules of genes involved in this critical development process. A number of the growth factors previously shown to be involved (e.g. PDGF and bFGF) were identified, but other factors known to be important for ovarian development were not identified. Often a reductionist approach such as a knockout mouse model can identify a factor as being important for the maintenance of tissue development or function, but this does not mean the factor is regulated during the process. In addition, critical developmental processes such as primordial follicle development often have a set of compensatory factors that have evolved such that loss of any one will still allow the process to proceed. Therefore, knockout models often do not have phenotypes for these factors. This does not mean the factor is not important, but instead that the developmental process is essential and thus multiple factors compensate to assure the developmental process occurs. The current study takes a systems biology approach to identify networks of genes involved in the process without the bias of a reductionist model. Therefore, novel groups of factors and cellular processes were identified that now require further investigation.

The integrative analysis revealed a gene sub-network involved in primordial follicle development to elucidate the basic developmental biology of this process and provide potential therapeutic targets for ovarian disease and function. This sub-network was validated by the presence of two genes previously identified as being important. A new gene identified, Ctgf, was tested and found to regulate primordial follicle development. Therefore, the network based systems biology approach was partially validated for a normal developmental process. This type of approach will likely be invaluable to study development on a systems biology level in the future.

## Materials and Methods

### Ovarian organ culture

Four-day old female Sprague-Dawley rats (Harlan Laboratories, Inc., USA) were euthanized according to the laboratories Washington State University IACUC approved (#02568-014) protocols and the ovaries removed and cultured whole as described previously [24]. Four-day old rat ovaries contain almost exclusively primordial follicles. For ovary culture experiments in which ovarian RNA was collected, 2–3 ovaries per well were cultured with media changes every 24 hours for two days in the absence (controls) or presence (treated) of either AMH (human Anti-Mülerian hormone)(50ng/mL, R&D Systems Inc., USA), FGF2 (rat Fibroblast growth factor 2)(50ng/mL, R&D Systems Inc., USA), BMP4 (human Bone morphogenetic protein 4)(50ng/mL, R&D Systems Inc., USA), GDNF (rat Glial derived neurotrophic factor)(50ng/mL, Calbiochem, USA), FGF7 (human fibroblast growth factor 7/keratinocyte growth factor)(50ng/mL, R&D Systems Inc., USA), KITLG (mouse Kit ligand)(50ng/mL, R&D Systems Inc., USA), LIF (rat leukemia inhibitory factor)(50ng/mL, Chemicon/Millipore, USA), PDGF-AB (rat platelet derived growth factor AB heterodimer)(50ng/mL, R&D Systems Inc., USA), TGFβ1 (human transforming growth factor beta 1)(50ng/mL, R&D Systems Inc., USA) or CTGF (human connective tissue growth factor)(50ng/mL, PeproTech Inc., NJ USA). After only two days of culture there are no morphological differences between control and growth factor-treated ovaries, so measurements of whole-ovary gene expression reflect differences in RNA transcription, rather than differing proportions of cell types due to differing cell proliferation between treatments.

In order to determine the effect of CTGF on primordial to primary follicle development, ovaries were cultured as above for ten days in the absence or presence of CTGF (50ng/mL), alone or in combination with TGF-beta1 (50ng/mL). After culture ovaries were fixed with Bouin's solution, paraffin embedded, sectioned onto microscope slides and stained with hematoxylin and eosin as described previously [24].

### Morphometric Analysis

The number of follicles at each developmental stage was counted and averaged in two serial sections from the largest cross-section through the center of the ovary. Total follicle number has not been found to change between treatment groups. Rather, only the percentage of follicles at each developmental stage changes with treatment [28], [34]. KL was used as a positive control for the organ culture experiments. Follicles in ovarian cross sections were classified as primordial (stage 0), or developing (stages 1–4: early primary, primary, transitional and preantral) as previously described [37]. Primordial follicles consist of an oocyte arrested in prophase I of meiosis that is partially or completely encapsulated by flattened squamous pregranulosa cells. Early transition primary follicles have initiated development (i.e., undergone primordial to primary follicle transition) and contain at least two cuboidal granulosa cells. Primary and preantral follicles exhibit one or more complete layers of cuboidal granulosa cells. Four-day old ovaries contain predominately primordial follicles [26], [38]. Hematoxylin/eosin stained ovarian sections were analyzed at 400× magnification using light microscopy. Follicles containing degenerating red eosin-stained oocytes were not counted.

### RNA preparation

RNA was isolated from whole rat ovaries after homogenization in one ml Trizol™ reagent (Sigma-Aldritch, USA), according to manufacturer's instructions. Two or three ovaries from the same culture well (from different rat pups out of the same litter) and receiving the same treatment were pooled and homogenized together. On any given day a culture experiment was performed, the treatment groups included untreated control ovaries and one to three different growth factor treatments. Homogenized samples were stored at −70 C until the time of RNA isolation. After isolation from Trizol, RNA was further purified using RNeasy MinElute Cleanup Kits (Qiagen, USA) and stored in aqueous solution at −70 C.

### Microarray Analysis

The microarray analysis was performed by the Genomics Core Laboratory, Center for Reproductive Biology, Washington State University, Pullman, WA using standard Affymetrix reagents and protocol. Briefly, mRNA was transcribed into cDNA with random primers, cRNA was transcribed, and single-stranded sense DNA was synthesized which was fragmented and labeled with biotin. Biotin-labeled ssDNA was then hybridized to the Rat Gene 1.0 ST microarrays containing 27,342 transcripts (Affymetrix, Santa Clara, CA, USA). Hybridized chips were scanned on Affymetrix Scanner 3000. CEL files containing raw data were then pre-processed and analyzed with Partek Genomic Suite 6.3 software (Partek Incorporated, St. Louis, MO) using an RMA GC-content adjusted algorithm. Raw data pre-processing was performed in 2 groups. The first group containing 38 samples CEL files were pre-processed in Partek all together as one experiment. Comparison of all array histogram graphs demonstrated the data for all 38 chips were similar and appropriate for further analysis. The second group of samples for microarray analysis consisting of 3 CTGF-treated and 3 corresponding control ovaries was run, pre-processed and analyzed *post factum*, separately from the rest of the samples as a result of a discovery from network analysis. Partek pre-processing algorithm for these 6 CEL files used the same criteria as used for the first group.

The microarray quantitative data involves over 10 different oligonucleotides arrayed for each gene and the hybridization must be consistent to allow a statistically significant quantitative measure of gene expression and regulation. In contrast, a quantitative PCR procedure only uses two oligonucleotides and primer bias is a major factor in this type of analysis. Therefore, we did not attempt to use PCR based approaches as we feel the microarray analysis is more accurate and reproducible without primer bias such as PCR based approaches.

All microarray CEL files (MIAME compliant raw data) from this study have been deposited with the NCBI gene expression and hybridization array data repository (GEO, http://www.ncbi.nlm.nih.gov/geo) (GEO Accession number: GSE20324), all arrays combined with one accession number, and can be also accessed through www.skinner.wsu.edu. For gene annotation, Affymetrix annotation file RaGene1_0stv1.na30.rn4.transcript.csv was used unless otherwise specified.

### Network analysis

The network analysis was restricted to genes differentially expressed between the control and the treatment groups based on previously established criteria: (1) fold change of group means ≥1.2 or ≤0.83; (2) T test p-value ≤0.05; and (3) absolute difference of group means ≥10. The union of the differentially expressed genes from the different treatments resulted in 1,540 genes being identified and used for constructing a weighted gene co-expression network [7], [8]. Unlike traditional un-weighted gene co-expression networks in which two genes (nodes) are either connected or disconnected, the weighted gene co-expression network analysis assigns a connection weight to each gene pair using soft-thresholding and thus is robust to parameter selection. The weighted network analysis begins with a matrix of the Pearson correlations between all gene pairs, then converts the correlation matrix into an adjacency matrix using a power function *f(x) = x^β^*. The parameter *β* of the power function is determined in such a way that the resulting adjacency matrix (i.e., the weighted co-expression network), is approximately scale-free. To measure how well a network satisfies a scale-free topology, we use the fitting index proposed by Zhang & Horvath [7] (i.e., the model fitting index *R^2^* of the linear model that regresses *log(p(k))* on *log(k)* where k is connectivity and *p(k)* is the frequency distribution of connectivity). The fitting index of a perfect scale-free network is 1. For this dataset, we select the smallest *β ( = 7)* which leads to an approximately scale-free network with the truncated scale-free fitting index *R^2^* greater than 0.75. The distribution *p(k)* of the resulting network approximates a power law: *p(k)∼k* ^−1.29^.

To explore the modular structures of the co-expression network, the adjacency matrix is further transformed into a topological overlap matrix [30]. As the topological overlap between two genes reflects not only their direct interaction, but also their indirect interactions through all the other genes in the network. Previous studies [7], [30] have shown that topological overlap leads to more cohesive and biologically meaningful modules. To identify modules of highly co-regulated genes, we used average linkage hierarchical clustering to group genes based on the topological overlap of their connectivity, followed by a dynamic cut-tree algorithm to dynamically cut clustering dendrogram branches into gene modules [39]. A total of sixteen modules were identified and the module size was observed to range from 20 to 194 genes.

To distinguish between modules, each module was assigned a unique color identifier, with the remaining, poorly connected genes colored grey. The hierarchical clustering over the topological overlap matrix (TOM) and the identified modules is shown. In this type of map, the rows and the columns represent genes in a symmetric fashion, and the color intensity represents the interaction strength between genes. This connectivity map highlights that genes in the ovary transcriptional network fall into distinct network modules, where genes within a given module are more interconnected with each other (blocks along the diagonal of the matrix) than with genes in other modules. There are a couple of network connectivity measures, but one particularly important one is the within module connectivity (k.in). The k.in of a gene was determined by taking the sum of its connection strengths (co-expression similarity) with all other genes in the module which the gene belonged.

### Gene Co-expression Network Analysis Clarification

Gene networks provide a convenient framework for exploring the context within which single genes operate. Networks are simply graphical models comprised of nodes and edges. For gene co-expression networks, an edge between two genes may indicate that the corresponding expression traits are correlated in a given population of interest. Depending on whether the interaction strength of two genes is considered, there are two different approaches for analyzing gene co-expression networks: 1) an unweighted network analysis that involves setting hard thresholds on the significance of the interactions, and 2) a weighted approach that avoids hard thresholds. Weighted gene co-expression networks preserve the continuous nature of gene-gene interactions at the transcriptional level and are robust to parameter selection. An important end product from the gene co-expression network analysis is a set of gene modules in which member genes are more highly correlated with each other than with genes outside a module. Most gene co-expression modules are enriched for GO functional annotations and are informative for identifying the functional components of the network that are associated with disease [6].

This gene co-expression network analysis (GCENA) has been increasingly used to identify gene sub-networks for prioritizing gene targets associated with a variety of common human diseases such as cancer and obesity [11], [12], [13], [14], [15]. One important end product of GCENA is the construction of gene modules comprised of highly interconnected genes. A number of studies have demonstrated that co-expression network modules are generally enriched for known biological pathways, for genes that are linked to common genetic loci and for genes associated with disease [6], [7], [11], [13], [14], [15], [40], [41], [42]. In this way, one can identify key groups of genes that are perturbed by genetic loci that lead to disease, and that define at the molecular level disease states. Furthermore, these studies have also shown the importance of the hub genes in the modules associated with various phenotypes. For example, GCENA identified ASPM, a hub gene in the cell cycle module, as a molecular target of glioblastoma [15] and MGC4504, a hub gene in the unfolded protein response module, as a target potentially involved in susceptibility to atherosclerosis [13].

### Pathway Analysis

Resulting lists of differentially expressed genes for each growth factor treatment as well as for each module generated in the network analysis were analyzed for KEGG (Kyoto Encyclopedia for Genes and Genome, Kyoto University, Japan) pathway enrichment using Pathway-Express, a web-based tool freely available as part of the Onto-Tools (http://vortex.cs.wayne.edu) [43]. Global literature analysis of various gene lists was performed using BiblioSphere PathwayEdition (Genomatix Software GmbH, Munchen, Federal Republic of Germany) software which performs pathway and interaction analysis and labels genes which belong to certain known metabolic and signal transduction pathways. A program based on literature analysis Pathway Studio (Ariadne, Genomics Inc. Rockville MD) was used to evaluate cellular processes connected to differentially expressed genes.

## Supporting Information

Figure S1 Network scheme for 1540 differentially expressed genes obtained by global literature analysis using BiblioSphere Pathway Edition Software (Genomatix Software GmbH, Munchen, Federal Republic of Germany). Different node colors represent different modules. A - the whole scheme clearly indicates 5 distinguished groups of genes (each group is shown separately on pp. 2–6) connected to 5 central genes: Nfkb1 (B, page 2), Vegfa (C, page 3), Egfr (D, page 4), and Gadd45a (F, page 6). Only connected genes are shown.
(2.07 MB PDF)

Figure S2 KEGG Pathway “Complement and Coagulation Cascades” enriched by regulated genes from 1,540 gene list. Red nodes represent up-regulated genes, blue - down-regulated, green - not affected genes.
(0.07 MB PDF)

Figure S3 Scheme of shortest connections to cellular processes for 55 candidate regulatory genes, as obtained by global literature analysis using Pathway Studio 7.0 (Ariadne Genomics, Inc., Rockville, MD; trial version). Only 22 connected genes from the list out of 55 are shown, the rest from the list are not connected and not shown. Node shapes code: oval and circle - protein; crescent - protein kinase and kinase; diamond - ligand; irregular polygon - phosphatase; circle/oval on tripod platform - transcription factor; ice cream cone - receptor. Red color represents up-regulated genes, blue color - down regulated genes, grey nodes represent genes closely connected (next neighbor) to the list genes; grey rectangles represent cell processes; arrows color: grey solid or dotted - regulation, blue - expression, green - promoter binding; arrows with plus sign show positive regulation/activation, arrows with minus sign - negative regulation/inhibition.
(0.35 MB PDF)

Table S1 Rat Genes Expressed Differentially After Growth Factor Treatment of Ovary. Legends: * - absolute value of difference between means of Control and GF Treatment expression values ** - abbreviations used for modules' color: trq -turquoise; brw - brown; blu- blue; ylw- yellow; prp - purple; gr - grey; grn - green; grlw - green-yellow; blc- black; mbl - midnight-blue; slm - salmon; lcn - light cyan; ***- k in. is connectivity coefficient determined in network analysis.
(1.37 MB PDF)
