# Supplementary figures and images for: Gene Bionetwork Analysis of Ovarian Primordial Follicle Development
Source: PLoS One. 2010 Jul 16;5(7):e11637. doi: 10.1371/journal.pone.0011637 (PMC2905436; doi:10.1371/journal.pone.0011637)

Figure S1(A)

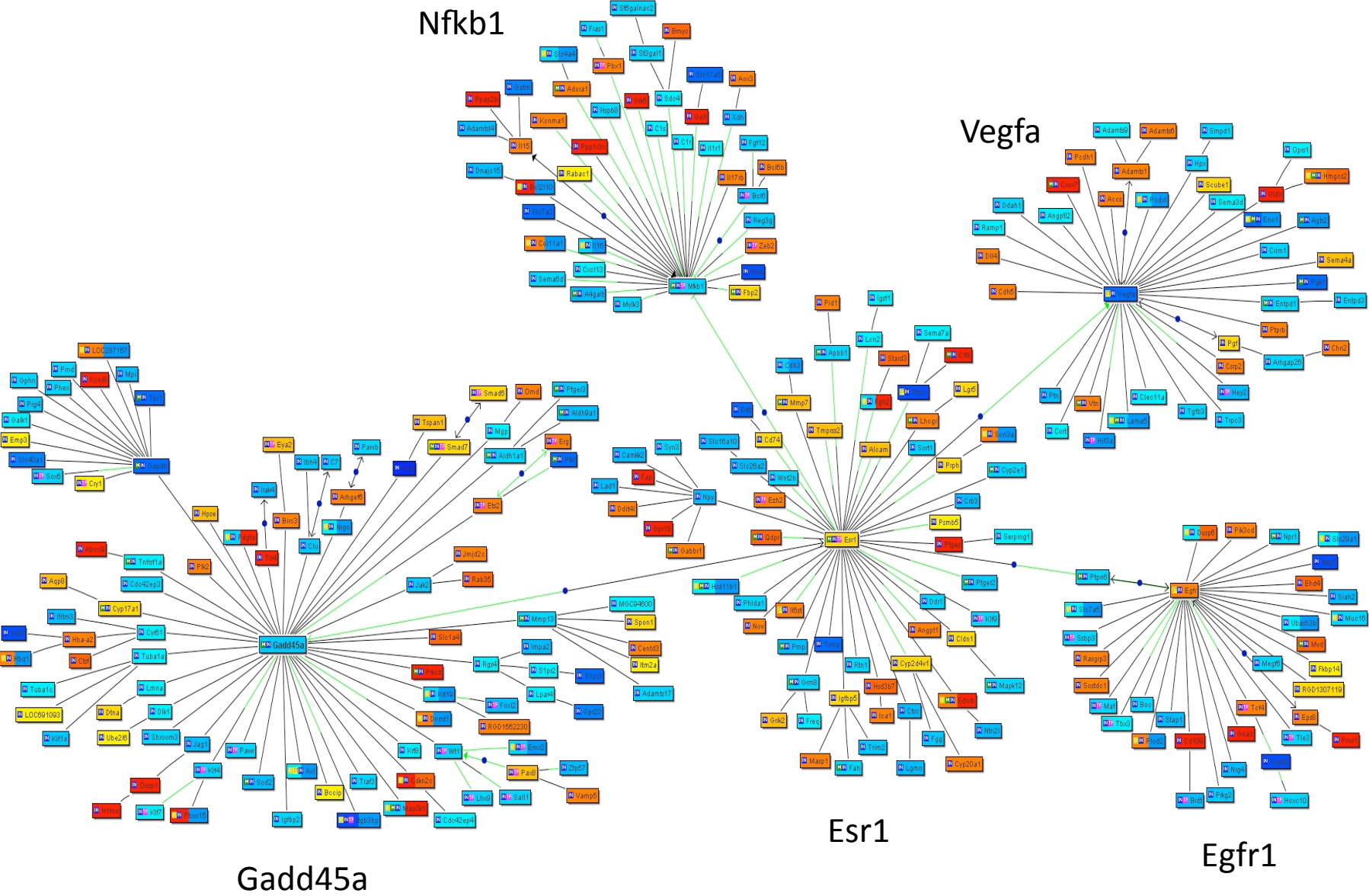

Figure S1(B)

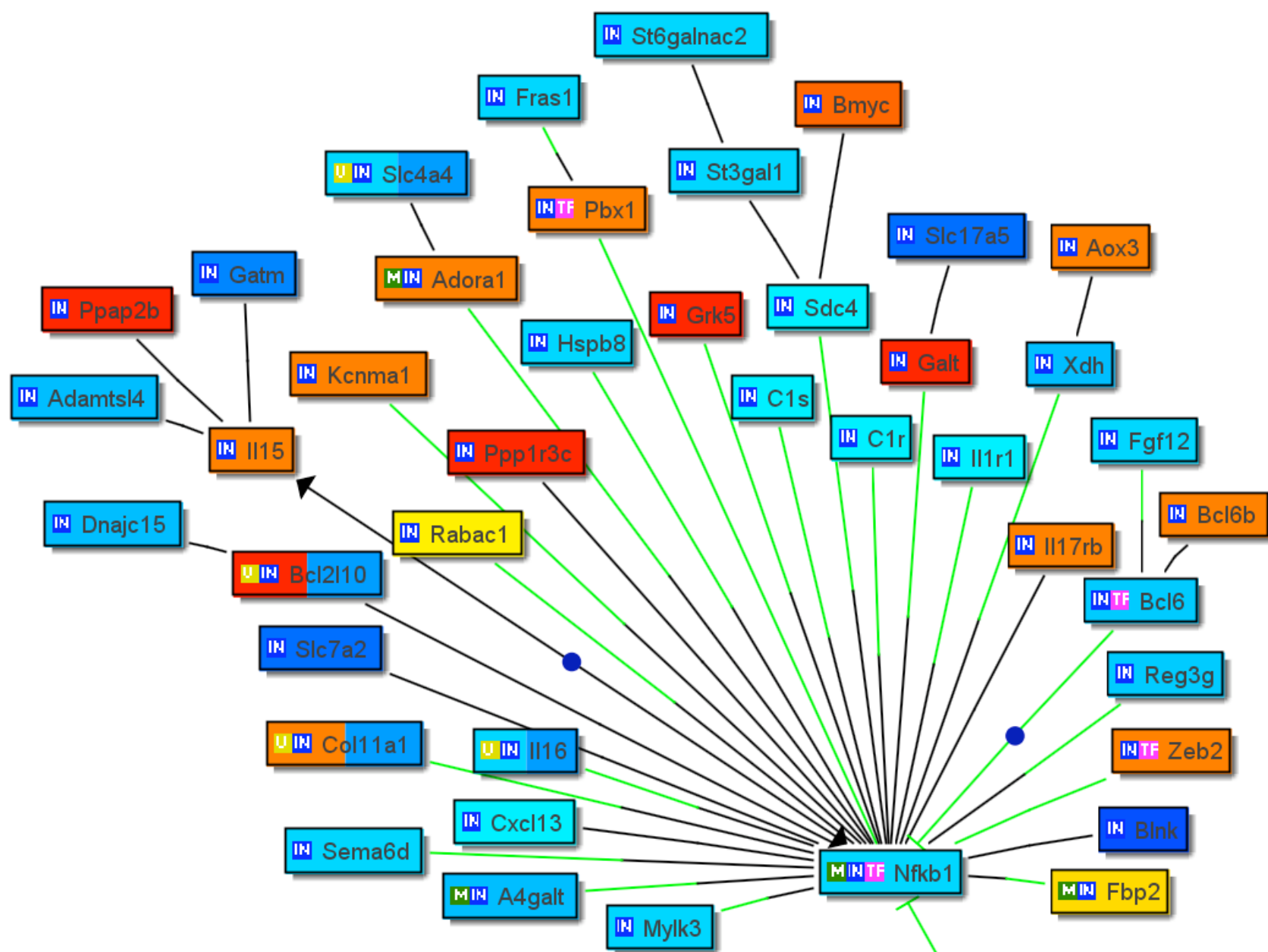

Figure S1(C)

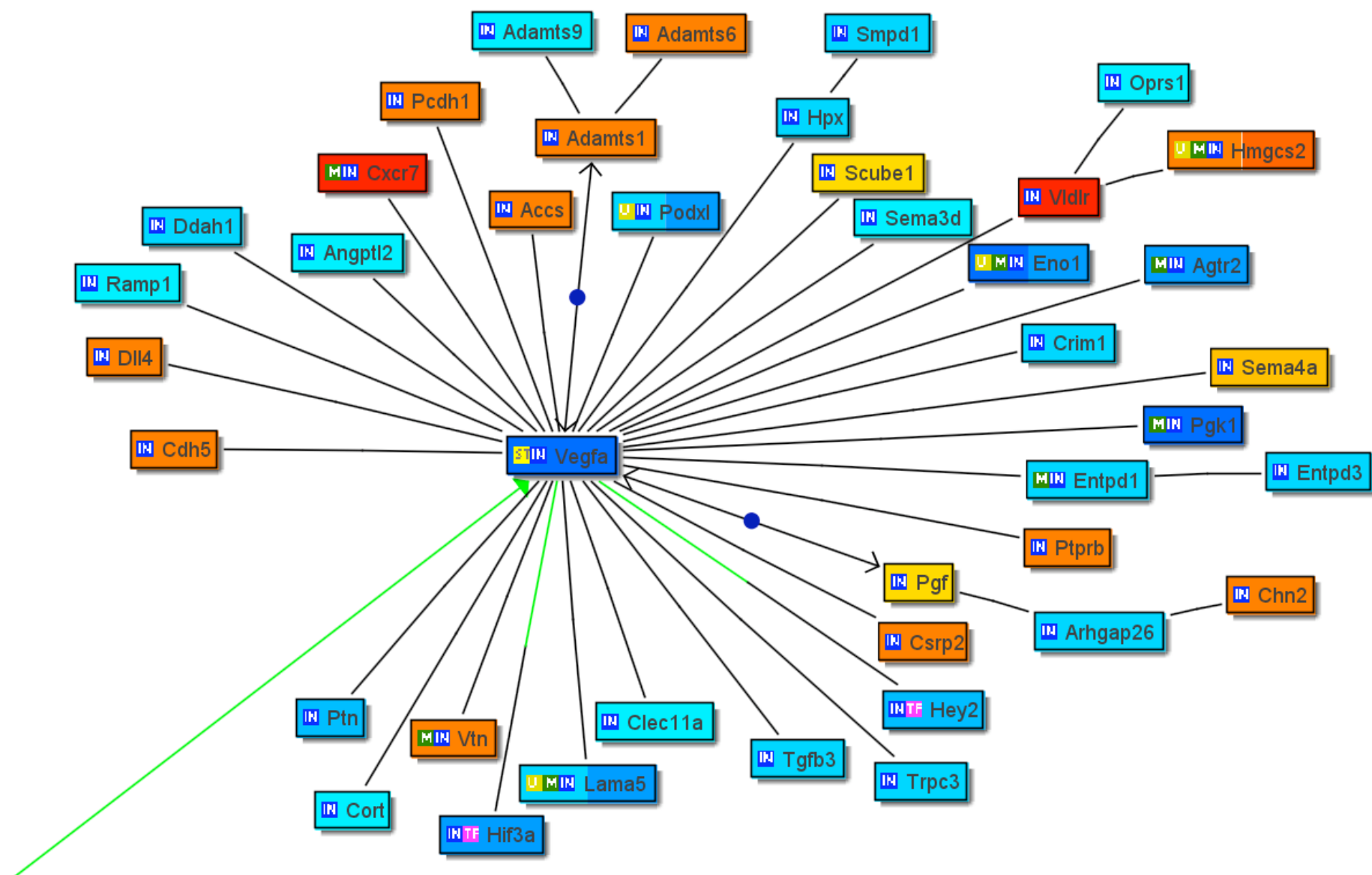

Figure S1(D)

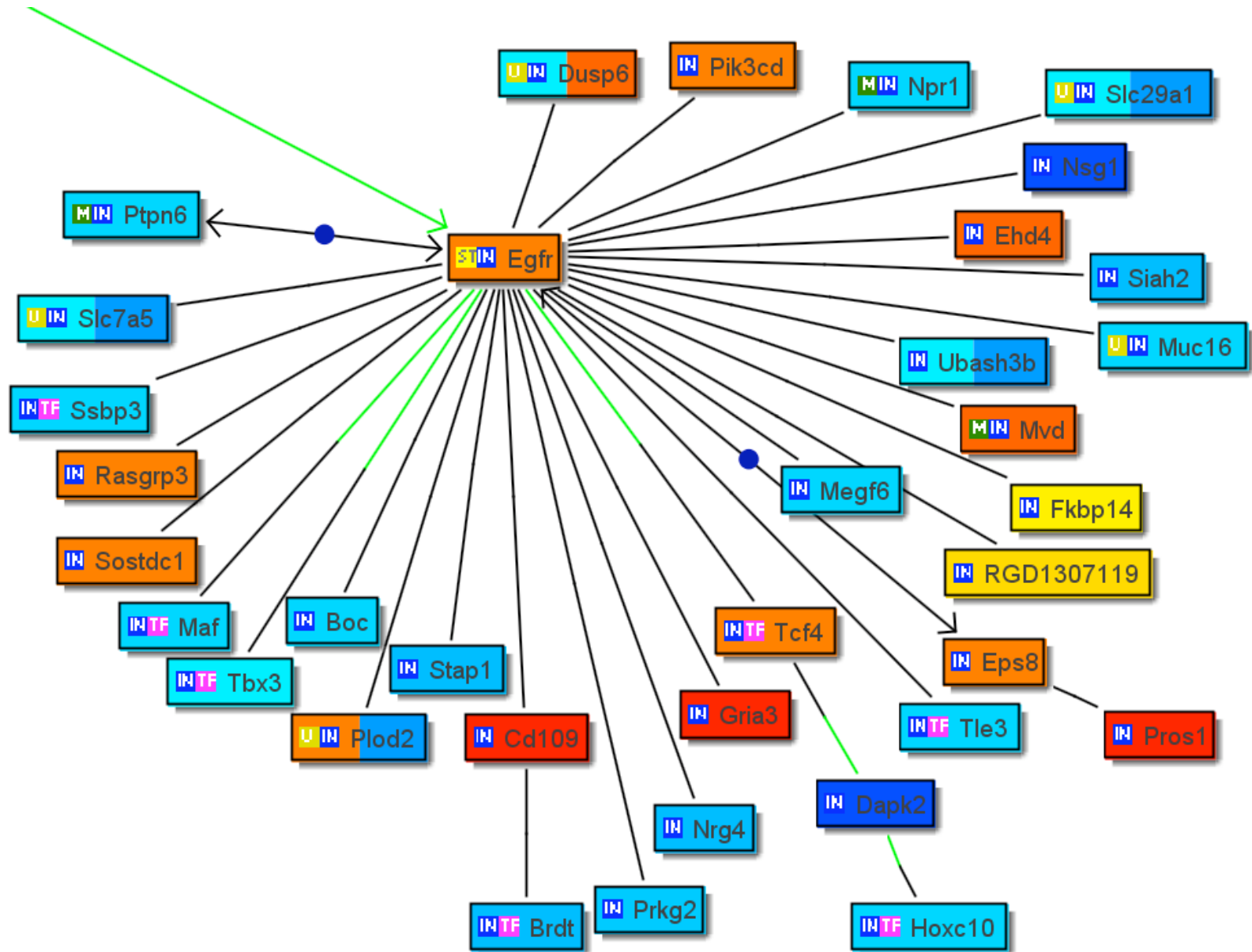

Figure S1(E)

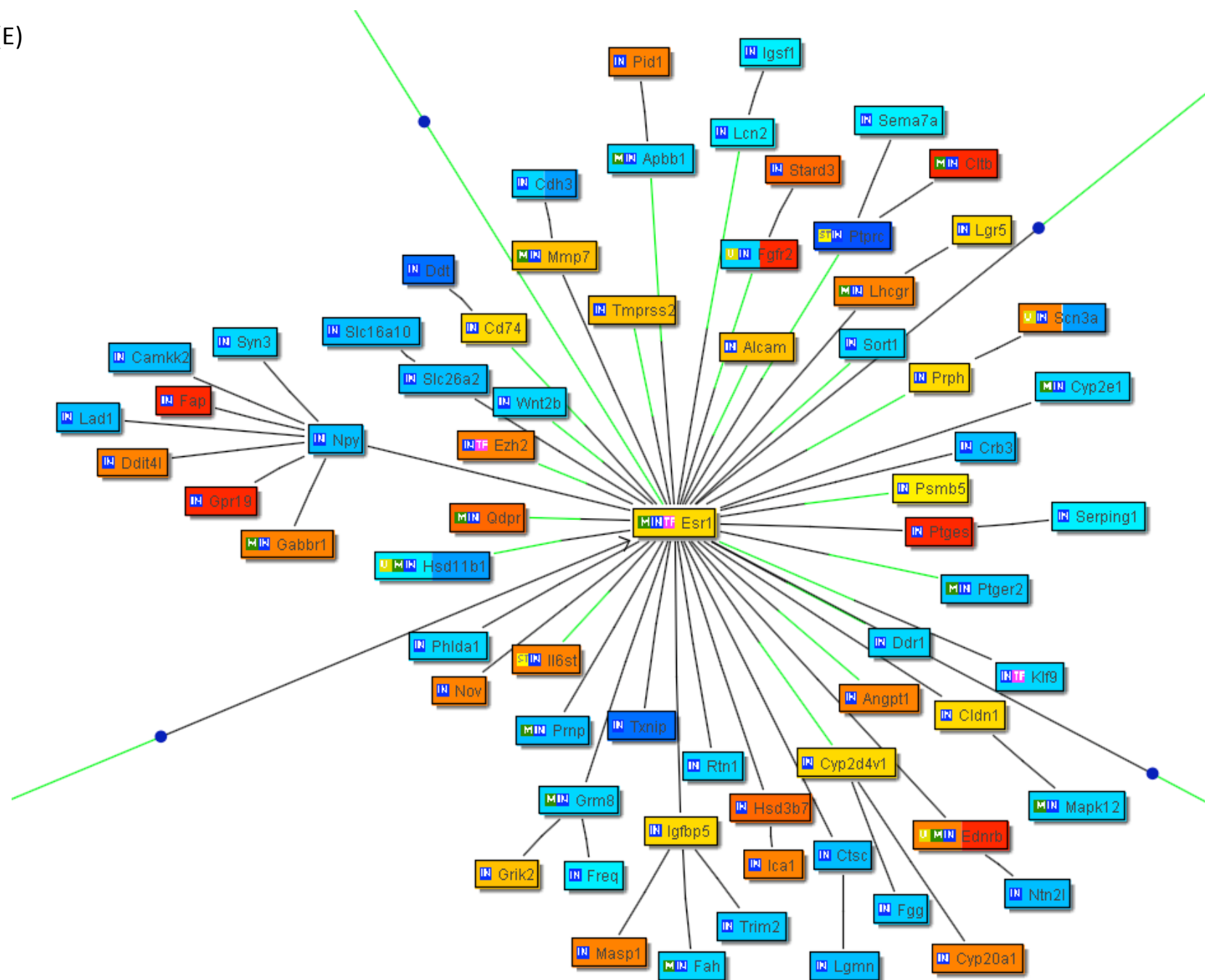

Figure S1(F)

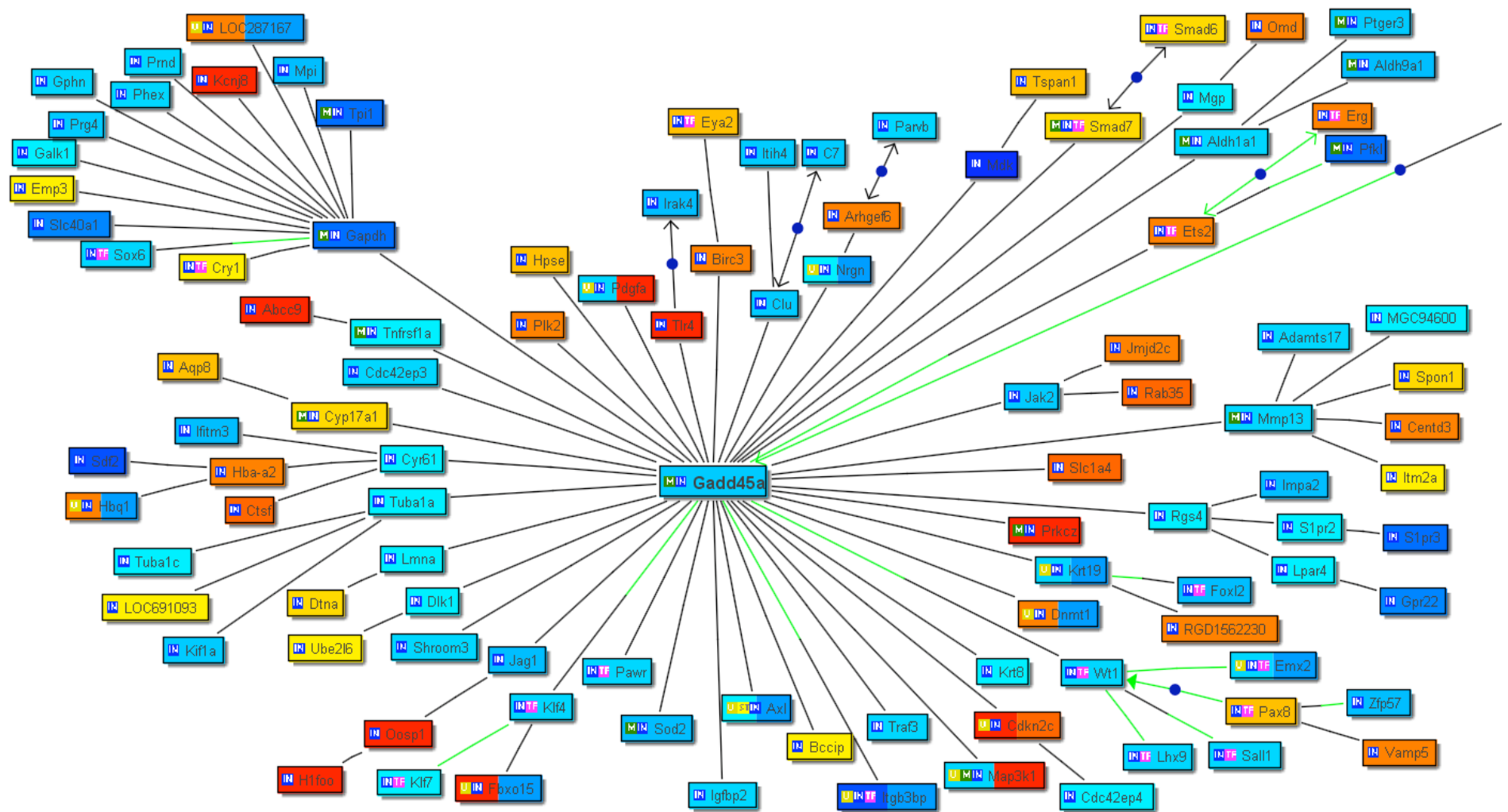

Supplement: Figure S1 — Network scheme for 1540 differentially expressed genes obtained by global literature analysis using BiblioSphere Pathway Edition Software (Genomatix Software GmbH, Munchen, Federal Republic of Germany). Different node colors represent different modules. A - the whole scheme clearly indicates 5 distinguished groups of genes (each group is shown separately on pp. 2–6) connected to 5 central genes: Nfkb1 (B, page 2), Vegfa (C, page 3), Egfr (D, page 4), and Gadd45a (F, page 6). Only connected genes are shown. (2.07 MB PDF) [file pone.0011637.s001.pdf]

# COMPLEMENT AND COAGULATION CASCADES

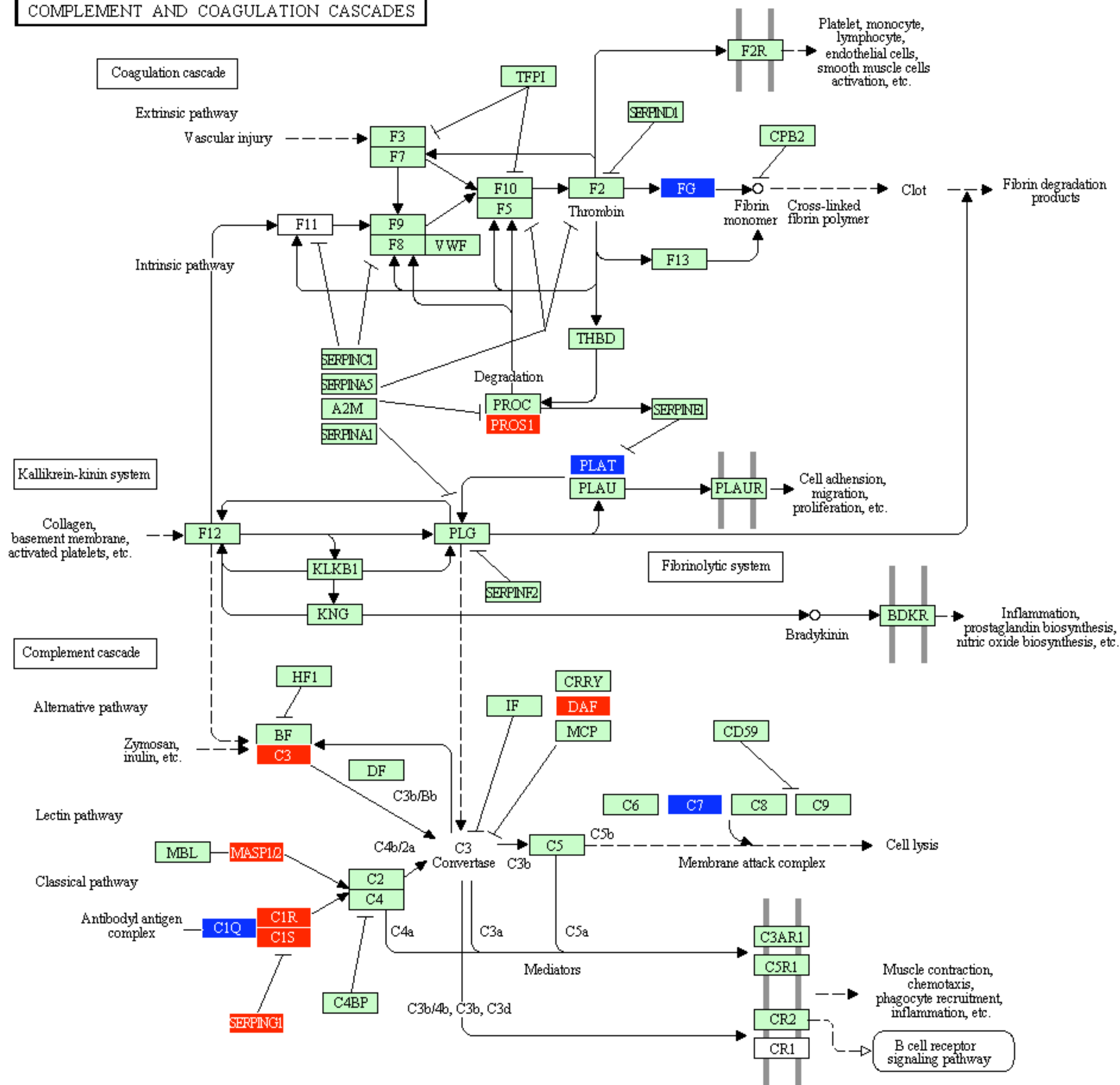

Supplement: Figure S2 — KEGG Pathway “Complement and Coagulation Cascades” enriched by regulated genes from 1,540 gene list. Red nodes represent up-regulated genes, blue - down-regulated, green - not affected genes. (0.07 MB PDF) [file pone.0011637.s002.pdf]

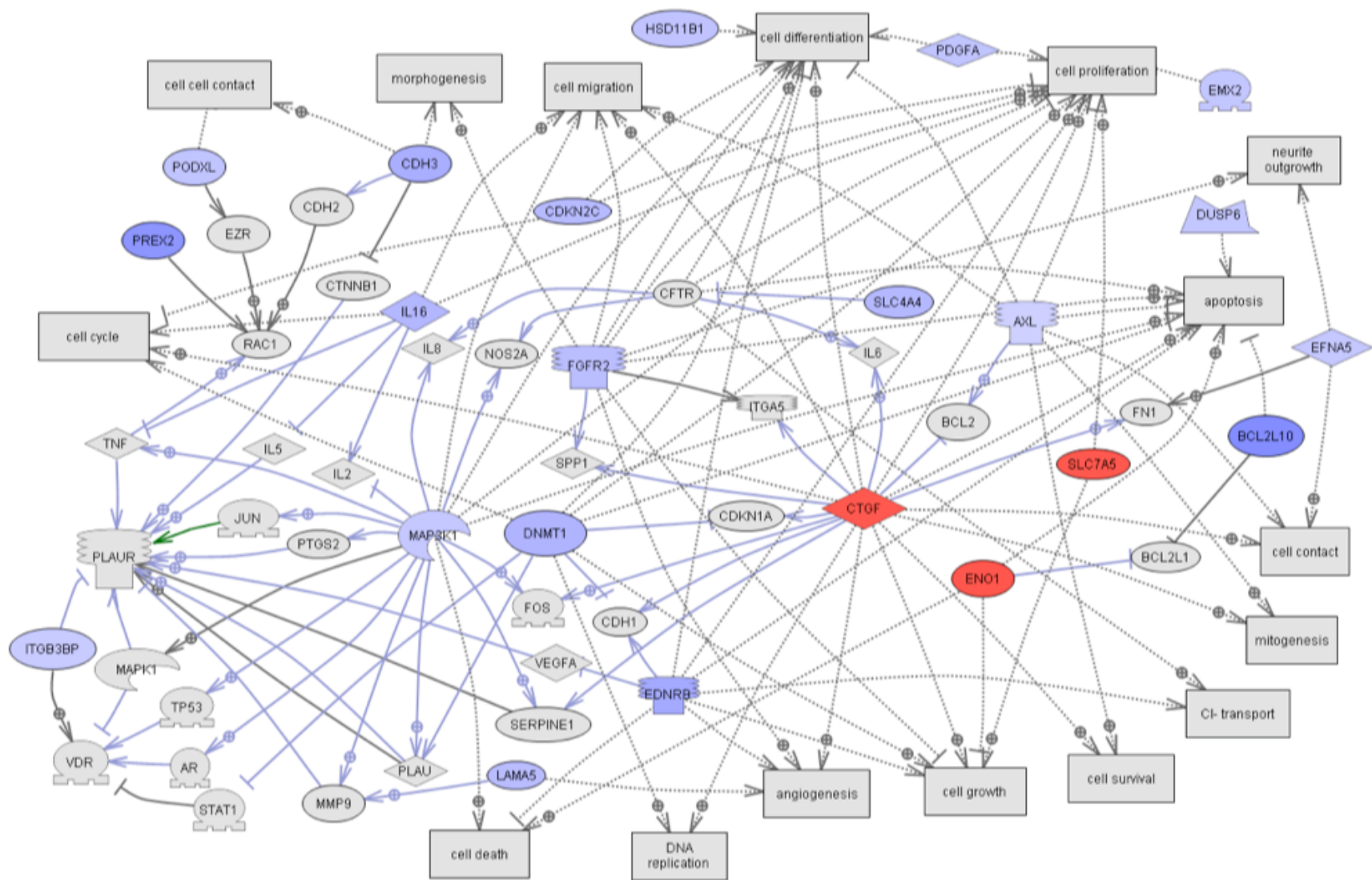

Supplement: Figure S3 — Scheme of shortest connections to cellular processes for 55 candidate regulatory genes, as obtained by global literature analysis using Pathway Studio 7.0 (Ariadne Genomics, Inc., Rockville, MD; trial version). Only 22 connected genes from the list out of 55 are shown, the rest from the list are not connected and not shown. Node shapes code: oval and circle - protein; crescent - protein kinase and kinase; diamond - ligand; irregular polygon - phosphatase; circle/oval on tripod platform - transcription factor; ice cream cone - receptor. Red color represents up-regulated genes, blue color - down regulated genes, grey nodes represent genes closely connected (next neighbor) to the list genes; grey rectangles represent cell processes; arrows color: grey solid or dotted - regulation, blue - expression, green - promoter binding; arrows with plus sign show positive regulation/activation, arrows with minus sign - negative regulation/inhibition. (0.35 MB PDF) [file pone.0011637.s003.pdf]
