# Supplementary material for: Gene Bionetwork Analysis of Ovarian Primordial Follicle Development
Source: PLoS One. 2010 Jul 16;5(7):e11637. doi: 10.1371/journal.pone.0011637 (PMC2905436; doi:10.1371/journal.pone.0011637)
Supplement: Table S1 — Rat Genes Expressed Differentially After Growth Factor Treatment of Ovary. Legends: * - absolute value of difference between means of Control and GF Treatment expression values ** - abbreviations used for modules' color: trq -turquoise; brw - brown; blu- blue; ylw- yellow; prp - purple; gr - grey; grn - green; grlw - green-yellow; blc- black; mbl - midnight-blue; slm - salmon; lcn - light cyan; ***- k in. is connectivity coefficient determined in network analysis. (1.37 MB PDF) [file pone.0011637.s004.pdf]

Table S1. Rat Genes Expressed Differentially After Growth Factor Treatment of Ovary

A. Genes influenced by treatment with Anti-Mullerian Hormone (AMH) - 268 probe sets

Gene Symbol	GF/Con Ratio	mean_ diff*	t-test p-value	Short 55 list	Module **	k.in ***	GenBank/ Transcript ID	Probe Set ID	Gene Title/Description
**Apoptosis**									
Dapk2	0.78	17	0.012		blu	5.32	NM_001013109	10911048	death-associated kinase 2
Moap1-ps1	1.25	33	0.026		trq	0.84	XM_001073080	10795986	modulator of apoptosis 1, pseudogene 1
Pawr	0.80	70	0.048		trq	21.19	NM_033485	10895337	PRKC, apoptosis, WT1, regulator
**Cell Cycle**									
Cdkl1	1.70	29	0.001		tan	3.02	NM_001025121	10890354	cyclin-dependent kinase-like 1 (CDC2-related kinase)
Cenpk	0.81	71	0.004		gr	0.00	NM_001106407	10812823	centromere protein K
Egr1	0.76	47	0.026		ylw	11.02	NM_012551	10800919	early growth response 1
**Cytoskeleton & ECM**									
Cdh1	1.30	51	0.011		mgt	0.49	NM_031334	10807542	cadherin 1
Cdh3	0.62	130	0.019	+	trq	28.03	XM_226426	10807525	cadherin 3, type 1, P-cadherin (placental)
Ceacam1	1.47	17	0.006		brw	6.10	NM_001033860	10719847	carcinoembryonic antigen-related cell adhesion molecule 1 (biliary glycoprotein)
Des	0.82	51	0.040		ylw	7.70	NM_022531	10924507	desmin
Esam	1.26	43	0.029		blu	3.01	NM_001004245	10909072	endothelial cell adhesion molecule
Krt19	0.72	721	0.004	+	trq	25.18	NM_199498	10747262	keratin 19
Kif26b	0.75	11	0.019		blu	2.65	NM_001109079	10766080	kinesin family member 26B
Lama5	0.71	82	0.042	+	trq	30.93	NC_005102	10852270	laminin, alpha 5
Muc16	0.59	272	0.009	+	trq	21.54	XM_001075437	10915239	mucin 16, cell surface associated
Myof	0.74	210	0.034		ylw	13.75	XM_220031	10729913	myoferlin
Myo5c	1.54	19	0.005		tan	2.35	NM_001108167	10911635	myosin VC
Rpa3	0.82	56	0.004		gr	0.00	NM_001106584	10860996	replication protein A3
Scin	1.32	24	0.033		brw	0.52	NM_198748	10889731	scinderin
Shroom3	0.68	73	0.048		trq	15.52	BC091437	10775782	shroom family member 3
RGD1563615	1.49	85	0.001		trq	5.71	XM_001064593	10793429	similar to Contactin associated protein-like 3 precursor (Cell recognition molecule Caspr3)
RGD1563615	1.44	67	0.021		trq	5.65	XM_001064593	10793431	similar to Contactin associated protein-like 3 precursor (Cell recognition molecule Caspr3)
LOC290704	0.73	79	0.006		trq	8.27	XM_001053156	10787921	similar to palladin
LOC290704	0.67	220	0.018		trq	10.43	XM_001053156	10787925	similar to palladin
LOC290704	0.74	59	0.020		trq	10.76	XM_001053156	10787918	similar to palladin
Tspan1	2.30	43	0.045		tan	6.06	NM_001004236	10878836	tetraspanin 1
Vcan	1.29	171	0.016		gr	0.00	AF072892	10820282	versican
**Development**									
Bnc1	0.73	121	0.045		trq	19.80	NM_001108916	10723339	basonuclin 1
Bnc2	0.82	114	0.020	+	trq	26.50	NM_001106666	10877880	basonuclin 2
Boll	0.83	31	0.029		trq	1.36	NM_001113370	10928177	bol, boule-like (Drosophila)
Crb2	0.82	11	0.033		trq	7.69	NM_001135761	10835944	crumbs homolog 2 (Drosophila)
Crim1	0.77	118	0.048		trq	22.01	XM_233798	10882399	cysteine rich transmembrane BMP regulator 1 (chordin like)
Egln3	0.79	45	0.002		prp	1.24	NM_019371	10889923	EGL nine homolog 3 (C. elegans)
Eya2	1.42	11	0.042		tan	3.44	NM_130427	10842298	eyes absent homolog 2 (Drosophila)
Fras1	0.59	122	0.044		trq	21.15	XM_214004	10775647	Fraser syndrome 1 homolog (human)
Hoxc5	0.78	54	0.044		trq	14.70	NM_001108116	10899609	homeo box C5
Sema4a	1.64	31	0.031		tan	3.97	NM_001012078	10824305	sema domain, immunoglobulin domain (Ig), transmembrane domain (TM) and short cytoplasmic

									domain, (semaphorin) 4A
Sema6d	0.74	168	0.028		trq	21.98	NM_001107768	10839327	sema domain, transmembrane domain (TM), and cytoplasmic domain, (semaphorin) 6D
RGD1307524	0.75	80	0.003		trq	2.85	BC166504	10729397	similar to Friedreich ataxia region gene X123
LOC683626	0.76	256	0.013		blu	9.76	NM_001129880	10888608	similar to limb-bud and heart
RGD1562629	1.24	53	0.042		red	0.39	XM_001059612	10823276	similar to neurobeachin
RGD1560686	0.75	32	0.027		trq	9.07	XM_001073292	10760559	similar to sidekick 1
RGD1560686	0.75	27	0.035		grlw	0.54	XM_001073292	10760516	similar to sidekick 1
Spata2L	0.81	11	0.044		blc	0.60	NM_001109133	10811747	spermatogenesis associated 2-like
Sox6	0.77	22	0.010		trq	12.17	NM_001024751	10725060	SRY (sex determining region Y)-box 6
Syn3	0.79	12	0.042		trq	4.42	NM_017109	10894465	synapsin III
Usmg5	0.78	151	0.008	+	blu	13.50	NM_133544	10730633	up-regulated during skeletal muscle growth 5 homolog (mouse)
Usmg5	0.80	125	0.018	+	blu	12.56	NM_133544	10747614	up-regulated during skeletal muscle growth 5 homolog (mouse)
Zpbp	0.65	93	0.003		trq	15.49	NM_001025139	10778472	zona pellucida binding protein
**Electron Transport**									
Cyct	0.71	34	0.018		gr	0.00	NM_012840	10846324	cytochrome c, testis
Cyp2c12	0.81	22	0.023		ylw	0.32	J03786	10930775	cytochrome P450, family 2, subfamily c, polypeptide 12
Cyp2e1	0.72	50	0.018		trq	5.51	NM_031543	10712090	cytochrome P450, family 2, subfamily e, polypeptide 1
Cyp1b1	0.61	86	0.045		trq	12.48	NM_012940	10882490	cytochrome P450, family 1, subfamily b, polypeptide 1
**Epigenetics**									
Cited4	1.37	40	0.002		brw	2.24	NM_053699	10871650	Cbp/p300-interacting transactivator, with Glu/Asp-rich carboxy-terminal domain, 4
Apeg3	0.71	56	0.044		trq	6.43	NM_001034160	10718666	antisense paternally expressed gene 3
LOC680312	0.75	31	0.003		blu	4.05	NM_001109400	10798475	similar to H2B histone family, member T
**Golgi Apparatus**									
Glt8d4	0.59	75	0.007		trq	21.10	XM_001066466	10857460	glycosyltransferase 8 domain containing 4
Man1a1	0.76	98	0.042		trq	18.24	NM_001033656	10833394	mannosidase, alpha, class 1A, member 1
Mfng	0.72	24	0.018		ylw	7.15	NM_199110	10905326	MFNG O-fucosylpeptide 3-beta-N-acetylglucosaminyltransferase
Rgp1	0.81	18	0.001		trq	1.38	XM_233379	10876416	RGP1 retrograde golgi transport homolog (S. cerevisiae)
LOC685402	0.73	38	0.020		trq	15.69	XM_001063652	10925678	similar to galactose-3-O-sulfotransferase 2
RGD1561381	1.30	58	0.005		gr	0.00	XM_573571	10771881	similar to microsomal glutathione S-transferase 3
St6galnac2	0.73	93	0.022		trq	3.17	NM_001031652	10749330	ST6 (alpha-N-acetyl-neuraminyl-2,3-beta-galactosyl-1,3)-N-acetylgalactosaminide alpha-2,6-sialyltransferase 2
B4galt6	0.82	53	0.048	+	blu	11.67	NM_031740	10803394	UDP-Gal:betaGlcNAc beta 1,4-galactosyltransferase, polypeptide 6
**Growth Factors, Cyto-, and Chemokines**									
Creg1	1.42	156	0.027		blu	2.44	NM_001105966	10765335	cellular repressor of E1A-stimulated genes 1
Gas2	0.78	20	0.007		trq	2.13	NM_001127504	10707338	growth arrest-specific 2
Il16	0.68	164	0.024	+	trq	31.93	NM_001105749	10723351	interleukin 16
Megf6	0.71	56	0.007		trq	20.73	NM_022955	10874458	multiple EGF-like-domains

									6
Pdgfa	0.75	41	0.036	+	trq	27.00	NM_012801	10757129	platelet-derived growth factor alpha polypeptide
Wnt2b	0.63	77	0.010		trq	11.60	AF204873	10825696	wingless-type MMTV integration site family, member 2B
**Immune Response**									
Hmcn1	0.79	116	0.033		ylw	3.74	XM_222716	10768426	hemicentin 1
Igsf10	1.25	62	0.003		gr	0.00	NM_198768	10823368	immunoglobulin superfamily, member 10
Ly6g6e	1.26	97	0.002		trq	10.07	NM_001001972	10831229	lymphocyte antigen 6 complex, locus G6E
RT1-CE16	0.79	20	0.011		blu	0.30	NM_001008839	10833969	RT1 class I, CE16
Ny-sar-48	1.23	23	0.009		red	0.59	NM_001024971	10790739	sarcoma antigen NY-SAR-48
Thy1	1.32	24	0.040		ylw	1.75	NM_012673	10909407	Thy-1 cell surface antigen
**Metabolism & Transport**									
Oplah	0.78	24	0.011		ylw	12.94	NM_053904	10904880	5-oxoprolinase (ATP-hydrolysing)
Aldh1a1	1.29	192	0.018		trq	4.37	NM_022407	10714323	aldehyde dehydrogenase 1 family, member A1
Akr1c19	1.36	97	0.034		blu	2.88	NM_001100576	10796050	aldo-keto reductase family 1, member C19
Aqp8	2.10	197	0.035		tan	2.66	NM_019158	10710752	aquaporin 8
B3galtl	0.70	71	0.020		gr	0.00	XM_001071932	10759941	beta 1,3-galactosyltransferase-like
Car8	1.30	18	0.013		gr	0.00	NM_001009662	10875375	carbonic anhydrase 8
Catsper3	1.35	10	0.020		brw	2.24	NM_001106101	10797173	cation channel, sperm associated 3
Cp	1.21	98	0.001		gr	0.00	NM_012532	10814430	ceruloplasmin
Clic2	0.81	18	0.028		ylw	10.76	NM_001009651	10827592	chloride intracellular channel 2
Ddah1	0.83	74	0.035		trq	3.96	NM_022297	10819644	dimethylarginine dimethylaminohydrolase 1
Entpd1	1.26	66	0.031		trq	16.42	NM_022587	10715258	ectonucleoside triphosphate diphosphohydrolase 1
Ftl	0.81	161	0.045		blu	3.00	NM_022500	10706836	ferritin, light polypeptide
Fah	0.81	21	0.025		trq	8.45	NM_017181	10723441	fumarylacetoacetate hydrolase
Hpx	0.72	19	0.039		trq	0.81	NM_053318	10724483	hemopexin
Hpse	1.40	33	0.000		tan	2.25	NM_022605	10771389	heparanase
Idi1	1.47	137	0.029		tan	1.24	NM_053539	10799241	isopentenyl-diphosphate delta isomerase 1
Kirrel	0.68	121	0.013	+	ylw	14.13	NM_207606	10824123	kin of IRRE like (Drosophila)
Ldhc	1.30	28	0.007		red	0.99	NM_017266	10707142	lactate dehydrogenase C
Mgll	1.25	18	0.002		tan	0.57	NM_138502	10857130	monoglyceride lipase
Nos2	1.31	13	0.000		brw	2.37	NM_012611	10736312	nitric oxide synthase 2, inducible
Podxl	0.75	251	0.036	+	trq	26.60	NM_138848	10861662	podocalyxin-like
Kcna3	1.24	26	0.001		blu	2.67	NM_019270	10818239	potassium voltage-gated channel, shaker-related subfamily, member 3
Prnd	1.27	77	0.032		trq	10.50	NM_001102431	10840080	prion protein 2 (dublet)
Ptges	1.25	42	0.025		red	2.88	NM_021583	10844223	prostaglandin E synthase
Rtn1	0.62	60	0.006		trq	20.33	NM_053865	10890522	reticulon 1
RGD1562033	0.83	71	0.018		gr	0.00	XR_008445	10880562	similar to peptidylprolyl isomerase A (cyclophilin A))
Slc16a14	1.49	19	0.032		tan	3.19	NM_001108229	10929536	solute carrier family 16, member 14 (monocarboxylic acid transporter 14)
Slc28a3	0.83	14	0.015		gr	0.00	NM_080908	10793799	solute carrier family 28 (sodium-coupled nucleoside transporter), member 3
Slc4a4	0.69	198	0.011	+	trq	28.98	NM_053424	10775997	solute carrier family 4 (anion exchanger), member 4
Smpd1	0.71	243	0.011		trq	19.39	NM_001006997	10709575	sphingomyelin phosphodiesterase 1, acid lysosomal

Tmc4	1.77	33	0.016		tan	4.23	NM_001034104	10703723	transmembrane channel-like 4
**Proteolysis**									
Adam33	0.71	51	0.003		ylw	4.61	NM_001107776	10850015	ADAM metallopeptidase domain 33
Adamts17	0.62	73	0.002		trq	10.03	XM_218753	10707862	ADAM metallopeptidase with thrombospondin type 1 motif, 17
Adamts4	1.22	14	0.004		grn	0.83	AB042272	10765534	ADAM metallopeptidase with thrombospondin type 1 motif, 4
Mmp7	5.30	245	0.029		tan	6.39	NM_012864	10907945	matrix metallopeptidase 7
Prss23	0.74	22	0.027		trq	5.61	NM_001007691	10708541	protease, serine, 23
Rnf43	0.76	19	0.017		trq	6.82	NM_001135921	10737252	ring finger protein 43
Spint1	1.36	35	0.027		brw	6.11	NM_001004265	10838792	serine peptidase inhibitor, Kunitz type 1
Tmprss2	1.58	13	0.033		tan	4.17	NM_130424	10753444	transmembrane protease, serine 2
**Receptors & Binding Proteins**									
Apbb1	0.78	25	0.048		trq	19.75	NM_080478	10724467	amyloid beta (A4) precursor protein-binding, family B, member 1 (Fe65)
Crhr2	0.76	11	0.036		gr	0.00	NM_022714	10862677	corticotropin releasing hormone receptor 2
Ddr1	0.77	92	0.049		trq	21.77	NM_013137	10827916	discoidin domain receptor tyrosine kinase 1
Ednra	1.23	124	0.033		red	2.21	NM_012550	10807022	endothelin receptor type A
Fgfr2	0.72	401	0.045	+	trq	30.76	NM_012712	10726172	fibroblast growth factor receptor 2
Gphn	0.78	124	0.006		trq	5.17	NM_022865	10885484	gephyrin
Igfbp2	0.65	121	0.040		trq	14.34	NM_013122	10924223	insulin-like growth factor binding protein 2
Il1r1	0.80	97	0.049		ylw	13.96	NM_013123	10922826	interleukin 1 receptor, type I
Npr1	0.70	58	0.013		trq	22.42	NM_012613	10824655	natriuretic peptide receptor A/guanylate cyclase A (atrionatriuretic peptide receptor A)
Nrp1	1.21	71	0.001		brw	1.84	NM_145098	10808959	neuropilin 1
Olr1666	0.76	18	0.006		mbl	0.78	NM_001000108	10809866	olfactory receptor 1666
Pdlim7	0.79	39	0.024		ylw	10.94	NM_173125	10794016	PDZ and LIM domain 7
Plxna4a	0.66	173	0.011	+	trq	30.89	NM_001107852	10861678	plexin A4, A
Sort1	0.76	76	0.039		trq	24.43	NM_031767	10818326	sortilin 1
Tmem151a	0.71	62	0.026	+	trq	26.21	NM_001107570	10727725	transmembrane protein 151A
Vom2r16	0.71	28	0.040		trq	5.96	NM_001099655	10703593	vomeronasal 2 receptor, 16
**Signaling**									
Alcam	1.37	284	0.004		tan	1.63	NM_031753	10750878	activated leukocyte cell adhesion molecule
Cdc42ep1	0.69	43	0.028		trq	22.47	NM_001079700	10897480	CDC42 effector protein (Rho GTPase binding) 1
Dusp4	0.76	19	0.040	+	ylw	17.30	NM_022199	10792035	dual specificity phosphatase 4
Entpd3	0.77	28	0.024		trq	15.81	NM_178106	10914354	ectonucleoside triphosphate diphosphohydrolase 3
Efna5	0.77	23	0.011	+	ylw	15.03	NM_053903	10930204	ephrin A5
Grik2	1.42	11	0.023		tan	3.15	NM_019309	10833910	glutamate receptor, ionotropic, kainate 2
Gnb4	1.22	84	0.047		trq	11.92	NM_001013910	10822717	guanine nucleotide binding protein (G protein), beta polypeptide 4
Hspb8	0.71	24	0.026		trq	8.23	NM_053612	10758930	heat shock protein 8
Mapk12	0.74	26	0.041		trq	10.07	NM_021746	10906142	mitogen-activated protein kinase 12
Nrgn	0.76	16	0.017	+	ylw	16.11	NM_024140	10916228	neurogranin
Pak6	0.65	20	0.009		ylw	11.45	NM_001106498	10838670	p21 protein (Cdc42/Rac)-activated kinase 6
Pde11a	0.66	26	0.045		trq	16.91	NM_001127481	10846301	phosphodiesterase 11A
Pde1b	0.61	194	0.042		trq	14.12	NM_022710	10899676	phosphodiesterase 1B, calmodulin-dependent
Phlda1	0.76	105	0.040		trq	6.91	NM_017180	10895406	pleckstrin homology-like

									domain, family A, member 1
Plk2	1.27	231	0.037		brw	8.63	NM_031821	10812954	polo-like kinase 2 (Drosophila)
Ptpn6	0.82	25	0.037		trq	8.55	NM_053908	10865463	protein tyrosine phosphatase, non-receptor type 6
Rab6b	0.61	39	0.024		trq	16.66	NM_001108775	10912631	RAB6B, member RAS oncogene family
Ror2	0.73	38	0.014		ylw	7.77	NM_001107339	10797499	receptor tyrosine kinase-like orphan receptor 2
Arhgap26	0.69	99	0.035		trq	19.87	NM_001107389	10801308	Rho GTPase activating protein 26
Shc4	0.77	15	0.028	+	ylw	16.97	XM_001054695	10849423	SHC (Src homology 2 domain containing) family, member 4
Smad7	0.78	25	0.033		mgt	4.73	NM_030858	10802734	SMAD family member 7
S1pr3	1.21	68	0.010		prp	0.80	XM_225216	10797566	sphingosine-1-phosphate receptor 3
Samd4a	0.75	30	0.019		trq	10.16	NM_001107254	10779654	sterile alpha motif domain containing 4A
Susd2	1.53	47	0.044		tan	3.89	NM_001106381	10832496	sushi domain containing 2
Tbc1d2b	0.72	144	0.034		trq	23.16	NM_001108175	10919283	TBC1 domain family, member 2B
Tns4	0.76	15	0.005		mgt	4.05	NM_001024881	10747011	tensin 4
**Transcription**									
Ccdc46	0.82	90	0.029		trq	11.44	NM_001105849	10739277	coiled-coil domain containing 46
Dpf3	1.30	40	0.020		trq	8.39	XM_238462	10891013	D4, zinc and double PHD fingers, family 3
Ebf1	1.20	14	0.030		brw	2.05	NM_053820	10732941	early B-cell factor 1
Etv4	0.80	19	0.048		ylw	13.65	NM_001108299	10747665	ets variant 4
Etv5	0.78	15	0.011	+	ylw	14.36	NM_001107082	10752034	ets variant 5
Hoxc10	0.74	11	0.006		trq	7.49	XM_235698	10899594	homeo box C10
Jund	0.76	14	0.020		blu	3.72	NM_138875	10787505	Jun D proto-oncogene
Klf4	0.75	43	0.011		trq	14.99	NM_053713	10876838	Kruppel-like factor 4 (gut)
Klf9	0.75	12	0.000		trq	9.13	NM_057211	10714413	Kruppel-like factor 9
Lhx9	0.65	115	0.027		trq	20.54	NM_181367	10768194	LIM homeobox 9
Npas3	0.82	11	0.030		trq	2.32	NM_001106727	10884599	neuronal PAS domain protein 3
Nfkb1	0.82	88	0.041		trq	18.73	XM_342346	10826918	nuclear factor of kappa light polypeptide gene enhancer in B-cells 1
Pax8	1.34	18	0.016		tan	0.11	NM_031141	10843216	paired box 8
Rfc4	0.78	191	0.003		gr	0.00	NM_001105869	10751973	replication factor C (activator 1) 4
Sall1	0.71	31	0.031		trq	8.16	NM_001107415	10806314	sal-like 1 (Drosophila)
Sertad1	0.73	20	0.049		gr	0.00	NM_001007735	10705364	SERTA domain containing 1
LOC690012	0.82	30	0.050		blu	7.76	XM_001072919	10825094	similar to High mobility group protein 2 (HMG-2)
Sgef	0.83	18	0.050		trq	1.82	XM_227201	10815663	Src homology 3 domain-containing guanine nucleotide exchange factor
Tle3	0.80	33	0.015		trq	18.79	NM_053400	10910641	transducin-like enhancer of split 3 (E(sp1) homolog, Drosophila)
Zc3h15	0.73	37	0.042		gr	0.00	NM_001010963	10764523	zinc finger CCCH-type containing 15
Zfp57	1.21	269	0.003		grn	5.15	NM_213565	10830888	zinc finger protein 57
**Translation & Protein Modification**									
Lsm5	0.78	28	0.024		blu	2.90	NM_001107289	10862728	LSM5 homolog, U6 small nuclear RNA associated (S. cerevisiae)
Mrps18b	1.24	49	0.024		pink	0.75	NM_212534	10827870	mitochondrial ribosomal protein S18B
Padi2	1.65	32	0.020		tan	3.48	NM_017226	10873580	peptidyl arginine deiminase, type II
LOC365287	0.75	29	0.005		blu	5.62	XR_006064	10722816	similar to 60S ribosomal protein L26
LOC680988	0.80	21	0.024		gr	0.00	XM_001057986	10858275	similar to ribosomal protein S12

Ssbp3	0.82	154	0.035		trq	14.57	NM_053358	10870609	single stranded DNA binding protein 3
Sumo1	0.78	48	0.001		gr	0.00	NM_001009672	10928423	SMT3 suppressor of mif two 3 homolog 1 (S. cerevisiae)
**Miscellaneous & Unknown**									
LOC686725	0.56	171	0.005	+	trq	25.88	XM_001075418	10915208	hypothetical protein LOC686725
Lrrn4	0.60	246	0.006		trq	15.66	XM_230550	10850170	leucine rich repeat neuronal 4
Rwdd2a	0.82	20	0.005		grn	1.54	NM_001108773	10912048	RWD domain containing 2A
RGD1559695	0.75	13	0.038		gr	0.00	XM_576290	10904460	similar to FLJ43860 protein
RGD1308489	0.81	313	0.007		mgt	3.44	XR_007342	10869772	similar to hypothetical protein
RGD1561503	0.76	44	0.025		gr	0.00	XM_574857	10821623	similar to hypothetical protein AN1443.2
LOC500300	1.20	19	0.009		gr	0.00	AY931022	10858160	similar to hypothetical protein MGC6835
RGD1306622	0.57	155	0.005	+	trq	32.56	XM_001074493	10728647	similar to KIAA0954 protein
RGD1309696	0.67	23	0.036		ylw	13.27	XM_234572	10892330	similar to KIAA2019 protein
RGD1566029	0.81	14	0.015		blc	0.23	XM_576321	10905909	similar to mKIAA1644 protein
LOC681383	0.82	20	0.001		trq	7.52	NM_001109441	10782199	similar to Protein C10orf11 homolog
**EST's**									
LOC363060	2.90	312	0.002		tan	5.24	NM_001014209	10909866	similar to RIKEN cDNA 1600029D21
LOC497860	0.80	117	0.010		blu	4.64	BC103490	10740496	similar to RIKEN cDNA 4930517K11
MGC105649	0.72	188	0.027		trq	22.31	NM_001008518	10839307	hypothetical LOC302884
RGD1309360	0.79	11	0.034		trq	12.77	NM_001012347	10821184	hypothetical LOC294715
RGD1563680	0.78	63	0.050		trq	5.47	XM_575206	10838559	similar to CDNA sequence BC052040
RGD1565514	0.78	13	0.030		trq	5.23	XM_001058342	10939842	similar to RIKEN cDNA 4933424A10 gene
	0.83	36	0.004		pink	0.97	6683748	10748234	
	0.80	29	0.045		trq	3.55	21595273	10877888	
	0.63	42	0.000		trq	2.21	37727288	10760298	
	0.78	14	0.013		pink	1.01	41152527	10701814	
	1.26	104	0.004		gr	0.00	AF090348	10919712	
	0.75	10	0.015		trq	3.43	ENSRNOT0000 0017435	10838240	
	0.74	12	0.036		trq	16.42	ENSRNOT0000 0023109	10935854	
	1.26	15	0.025		gr	0.00	ENSRNOT0000 0028945	10701697	
	0.61	135	0.020		trq	22.60	ENSRNOT0000 0031367	10765364	
	0.71	129	0.041		trq	22.62	ENSRNOT0000 0035129	10764376	
	0.80	139	0.040		pink	1.83	ENSRNOT0000 0040943	10813353	
	0.74	18	0.017		blu	4.85	ENSRNOT0000 0047723	10927610	
	0.79	20	0.022		gr	0.00	ENSRNOT0000 0050822	10852195	
	0.73	40	0.007		blc	1.13	ENSRNOT0000 0051386	10878386	
	1.27	16	0.005		blc	0.52	ENSRNOT0000 0052091	10824721	
	0.61	284	0.001		blu	15.72	ENSRNOT0000 0052442	10709825	
	0.62	21	0.018		blu	2.35	ENSRNOT0000 0052447	10844062	
	0.80	10	0.001		trq	1.14	ENSRNOT0000 0052452	10793367	
	0.67	31	0.030		blu	8.77	ENSRNOT0000 0052558	10778080	
	0.74	71	0.022		blu	5.88	ENSRNOT0000 0052577	10878961	
	0.73	107	0.021		blu	9.12	ENSRNOT0000	10828077	

							0052620		
	0.46	101	0.009		blu	11.84	ENSRNOT0000 0052710	10838282	
	0.46	101	0.009		blu	11.84	ENSRNOT0000 0052710	10924172	
	0.76	15	0.016		trq	2.02	ENSRNOT0000 0052740	10880880	
	0.76	13	0.014		blu	11.57	ENSRNOT0000 0052749	10779724	
	0.73	58	0.003		blu	6.80	ENSRNOT0000 0052882	10759461	
	0.62	63	0.045		blu	15.32	ENSRNOT0000 0052964	10801135	
	0.76	77	0.000		blu	6.08	ENSRNOT0000 0053015	10919224	
	0.69	21	0.031		blu	11.36	ENSRNOT0000 0053029	10774265	
	0.80	20	0.013		blc	0.15	ENSRNOT0000 0053042	10937568	
	0.82	11	0.001		blu	4.12	ENSRNOT0000 0053201	10819998	
	0.81	13	0.030		blu	2.22	ENSRNOT0000 0053250	10766287	
	0.50	276	0.024		blu	6.93	ENSRNOT0000 0053279	10708691	
	0.77	25	0.029		blu	7.53	ENSRNOT0000 0053296	10805849	
	0.54	275	0.026		blu	19.11	ENSRNOT0000 0053328	10855946	
	0.58	365	0.017		blu	18.42	ENSRNOT0000 0053328	10862359	
	0.80	17	0.018		blu	7.28	ENSRNOT0000 0053338	10791520	
	0.57	22	0.004		blu	3.46	ENSRNOT0000 0053441	10798025	
	0.62	105	0.019		blu	11.53	ENSRNOT0000 0053476	10702293	
	1.44	95	0.018		gr	0.00	ENSRNOT0000 0053496	10903998	
	0.74	66	0.040		blu	8.71	ENSRNOT0000 0053821	10805744	
	0.66	52	0.015		blu	15.40	ENSRNOT0000 0053951	10703224	
	1.23	21	0.017		grn	8.02	ENSRNOT0000 0054280	10886846	
	0.73	23	0.000		blu	1.21	ENSRNOT0000 0054339	10851378	
	0.57	56	0.015		blu	13.19	ENSRNOT0000 0054517	10797013	
	0.57	56	0.015		blu	13.19	ENSRNOT0000 0054517	10932228	
	0.78	180	0.038		blu	3.56	ENSRNOT0000 0054649	10930226	
	0.83	38	0.024		pink	0.98	ENSRNOT0000 0054734	10715250	
	0.58	33	0.001		red	0.10	ENSRNOT0000 0055721	10866408	
	0.69	94	0.042		pink	3.72	ENSRNOT0000 0056162	10872699	
	0.67	55	0.004		gr	0.00	ENSRNOT0000 0057306	10815996	
	0.81	27	0.001		gr	0.00	ENSRNOT0000 0057420	10833416	
	0.69	19	0.025		blu	1.15	ENSRNOT0000 0058916	10773002	
	0.81	27	0.001		gr	0.00	ENSRNOT0000 0059477	10791602	
	1.34	24	0.031		tan	2.67	ENSRNOT0000 0059557	10732939	
	0.72	20	0.005		trq	14.01	ENSRNOT0000 0059999	10889627	
	0.76	14	0.018		blc	0.29	ENSRNOT0000	10894247	

							0060163		
	0.65	30	0.002		pink	0.81	ENSRNOT0000 0060601	10861211	
	0.81	40	0.031		blu	10.15	ENSRNOT0000 0062230	10892067	
	0.80	25	0.016		blu	4.26	ENSRNOT0000 0062811	10739864	
	1.55	31	0.000		tan	2.56	GENSCAN0000 0000428	10797966	
	0.48	56	0.004		blu	11.39	J01884	10830454	
	0.74	29	0.017		blc	0.36	NM_001111127	10798473	
	1.20	14	0.001		red	0.44	rno-mir-103-2	10840061	MI0000887 Rattus norvegicus miR-103-2 stem-loop
	0.81	14	0.007		trq	2.16	rno-mir-320	10781353	MI0000972 Rattus norvegicus miR-320 stem-loop
	0.82	16	0.045		mbl	2.22	rno-mir-483	10727006	MI0003485 Rattus norvegicus miR-483 stem-loop

B. Genes influenced by treatment with fibroblast growth factor 2 (FGF2) - 248 probe sets									
**Apoptosis**									
**Gene Symbol**	**GF/Con Ratio**	**mean_ diff***	**t-test p-value**	**Short 55 list**	**Module ****	**k.in *****	**GenBank/ Transcript ID**	**Probe Set ID**	**Gene Title/Description**
Tnfsf10	0.76	39	0.029		blc	1.00	NM_145681	10814528	tumor necrosis factor (ligand) superfamily, member 10
**Cell Cycle**									
Cdca7l	0.77	75	0.022		brw	11.84	NM_001034953	10887802	cell division cycle associated 7 like
Cdkl3	0.80	43	0.005		pink	0.50	NM_021772	10733401	cyclin-dependent kinase-like 3
LOC367746	0.78	77	0.001		pink	1.79	BC086373	10937220	similar to Spindlin-like protein 2 (SPIN-2)
Ypel4	1.30	39	0.032		ylw	0.95	NM_001024369	10837337	yippee-like 4 (Drosophila)
**Cytoskeleton & ECM**									
Blnk	0.76	21	0.019		blu	0.43	NM_001025767	10730131	B-cell linker
Cdh6	0.81	11	0.021		tan	4.24	NM_012927	10822043	cadherin 6
Cdon	0.78	94	0.033		trq	8.56	NM_017358	10908968	Cdon homolog (mouse)
Cldn1	0.51	50	0.029		mgt	1.48	NM_031699	10751896	claudin 1
Cntn3	0.61	91	0.006		blu	1.50	NM_019329	10864481	contactin 3 (plasmacytoma associated)
Clec14a	1.51	59	0.000		red	2.07	NM_001014077	10890111	C-type lectin domain family 14, member a
Dtna	0.65	130	0.000		mgt	2.14	XM_001054487	10800471	dystrobrevin alpha
Emilin3	0.83	30	0.013		red	1.42	NM_001109901	10851402	elastin microfibril interfacer 3
Emp1	1.84	413	0.034		ylw	5.11	NM_012843	10859296	epithelial membrane protein 1
Emp3	1.31	558	0.043		pink	7.23	NM_030847	10721962	epithelial membrane protein 3
Fgl2	1.24	52	0.013		prp	0.56	NM_053455	10860231	fibrinogen-like 2
Frem2	0.64	115	0.010		trq	11.80	XM_227142	10823127	Fras1 related extracellular matrix protein 2
Gsn	1.69	173	0.048		ylw	3.89	NM_001004080	10835757	gelsolin
Ggta1	1.22	20	0.026		red	1.50	NM_145674	10844695	glycoprotein, alpha-galactosyltransferase 1
Prph	0.58	111	0.023		mgt	3.71	NM_012633	10899061	peripherin
Plp2	1.32	463	0.004		pink	5.43	NM_207601	10932534	proteolipid protein 2 (colonic epithelium-enriched)
LOC686753	0.69	36	0.033		trq	1.15	XM_001075443	10826866	similar to nephronectin isoform a
Sdc4	1.58	810	0.028		ylw	4.58	NM_012649	10851599	syndecan 4
Tnxa	0.76	12	0.001		mgt	0.38	NR_024118	10831384	tenascin XA
Tspan2	1.21	58	0.022		red	1.34	NM_022589	10817898	tetraspanin 2
Tmem204	1.55	91	0.000		ylw	1.13	NM_001009620	10741330	transmembrane protein 204
Tppp3	0.80	10	0.016		tan	0.44	NM_001009639	10810631	tubulin polymerization-promoting protein family member 3
**Development**									
Aamp	1.21	55	0.000		slm	2.09	NM_001106920	10928890	angio-associated, migratory cell protein
Bmper	0.70	27	0.017		grn	6.37	NM_001135799	10908645	BMP-binding endothelial regulator
Lrrn1	0.73	54	0.004		gr	0.00	NM_001037363	10857541	leucine rich repeat neuronal 1
Mpzl2	0.83	31	0.046		trq	3.73	NM_001106818	10909590	myelin protein zero-like 2
Rcan1	0.74	52	0.010		trq	7.11	NM_153724	10753214	regulator of calcineurin 1
Rai2	0.67	137	0.011		blu	0.83	NM_001109316	10937834	retinoic acid induced 2
Sectm1b	0.82	11	0.029		grn	2.69	NM_199082	10749762	secreted and transmembrane 1B
LOC691093	1.29	31	0.027		pink	2.83	NM_001109621	10900831	similar to gene trap ROSA b-geo 22
Tex101	0.72	41	0.004		gr	0.00	NM_139037	10719674	testis expressed 101
Trpc3	0.60	81	0.003		trq	2.70	NM_021771	10822888	transient receptor potential

									cation channel, subfamily C, member 3
**Electron Transport**									
Cyb561d2	1.31	29	0.013		slm	0.96	NM_001007753	10920078	cytochrome b-561 domain containing 2
Cyp17a1	0.73	11	0.036		mgt	0.77	NM_012753	10730599	cytochrome P450, family 17, subfamily a, polypeptide 1
Cyp2d4v1	1.29	17	0.019		mgt	1.12	NM_138515	10905721	cytochrome P450, family 2, subfamily d, polypeptide 4
**Epigenetics**									
Cav1	1.29	215	0.009		trq	1.99	NM_031556	10853816	caveolin 1, caveolae protein
H19	0.83	393	0.030		mgt	2.23	NR_027324	10726991	H19 fetal liver mRNA
Tmf1	0.81	73	0.024		gr	0.00	NM_053671	10864351	TATA element modulatory factor 1
Tbx1	0.79	15	0.020		gr	0.00	NM_001108322	10752295	T-box 1
**Golgi Apparatus**									
LOC685079	1.25	53	0.010		pink	3.33	XM_001062200	10842117	similar to Protein SYS1 homolog
**Growth Factors, Cyto-, and Chemokines**									
Ccl5	0.65	53	0.025		blu	1.05	NM_031116	10745631	chemokine (C-C motif) ligand 5
Cmklr1	1.35	33	0.006		blu	0.76	NM_022218	10759198	chemokine-like receptor 1
Pgf	0.67	81	0.001		mgt	1.15	NM_053595	10891240	placental growth factor
Sept4	1.32	83	0.006		red	1.15	NM_001011893	10737205	septin 4
Spon1	0.73	144	0.012		mgt	5.52	NM_172067	10710067	spondin 1, extracellular matrix protein
Wnt2b	0.44	117	0.001		trq	11.60	AF204873	10825696	wingless-type MMTV integration site family, member 2B
**Immune Response**									
Cd38	2.23	134	0.000		ylw	4.20	NM_013127	10777232	CD38 molecule
Cd74	0.43	205	0.011		mgt	2.74	NM_013069	10802013	Cd74 molecule, major histocompatibility complex, class II invariant chain
C3	0.58	292	0.028		trq	10.67	NM_016994	10931717	complement component 3
C4-2	0.65	154	0.007		grn	1.48	NM_001002805	10828229	complement component 4, gene 2
LOC300024	1.48	42	0.023		trq	1.99	XM_216959	10904587	similar to Ly6-B antigen gene
Ighg	0.73	19	0.043		blc	7.31	NM_001013945	10887657	Immunoglobulin heavy chain (gamma polypeptide)
**Metabolism & Transport**									
Aass	1.21	10	0.018		gr	0.00	NM_001100963	10861242	aminoadipate-semialdehyde synthase
Apobec1	0.75	17	0.045		mgt	2.22	NM_012907	10865329	apolipoprotein B mRNA editing enzyme, catalytic polypeptide 1
Atp5g2	1.22	225	0.011		pink	6.21	NM_133556	10907634	ATP synthase, H+ transporting, mitochondrial F0 complex, subunit C2 (subunit 9)
Atp1b2	0.81	30	0.007		grlw	0.88	NM_012507	10744098	ATPase, Na+/K+ transporting, beta 2 polypeptide
Ccbl1	0.72	56	0.035		gr	0.00	NM_001013164	10844149	cysteine conjugate-beta lyase, cytoplasmic
Doc2b	0.77	60	0.029		mgt	0.82	NM_031142	10744970	double C2-like domains, beta
Fbp2	0.64	43	0.008		mgt	5.30	NM_053716	10793589	fructose-1,6-bisphosphatase 2
Gatm	1.40	213	0.001		lcn	0.53	NM_031031	10849290	glycine amidinotransferase (L-arginine:glycine amidinotransferase)
Hsd3b7	1.24	40	0.001		slm	1.12	NM_139329	10711210	hydroxy-delta-5-steroid dehydrogenase, 3 beta- and steroid delta-isomerase 7
Hsd17b2	0.50	103	0.047		trq	5.91	NM_024391	10811341	hydroxysteroid (17-beta) dehydrogenase 2
Manba	1.38	123	0.003		ylw	2.37	NM_001031655	10819245	mannosidase, beta A, lysosomal

Mthfd1l	1.26	37	0.013		prp	2.91	NM_001108462	10702626	methylenetetrahydrofolate dehydrogenase (NADP+ dependent) 1-like
Mvd	1.28	36	0.007		slm	0.95	NM_031062	10811560	mevalonate (diphospho) decarboxylase
Pigs	1.20	47	0.000		slm	1.44	NM_001006602	10745081	phosphatidylinositol glycan anchor biosynthesis, class S
Pck2	1.22	29	0.009		slm	1.26	NM_001108377	10780360	phosphoenolpyruvate carboxykinase 2 (mitochondrial)
Ppa1	0.70	116	0.004		gr	0.00	NM_001100834	10830038	pyrophosphatase (inorganic) 1
Qdpr	1.21	77	0.006		slm	1.09	NM_022390	10773080	quinoid dihydropteridine reductase
Rpgrip1l	0.80	32	0.049		gr	0.00	NM_001107414	10806257	Rpgrip1-like
Scamp4	1.29	85	0.035		pink	4.87	NM_031725	10900478	secretory carrier membrane protein 4
Sh3bgrl2	1.29	53	0.047		trq	2.43	NM_001137647	10911936	SH3 domain binding glutamic acid-rich protein like 2
RGD1561661	0.67	17	0.042		mgt	1.96	NM_001106954	10932341	similar to Ferritin light chain (Ferritin L subunit)
Smpx	0.60	44	0.000		gr	0.00	NM_053395	10938060	small muscle protein, X-linked
Slc1a4	1.31	33	0.037		slm	0.60	NM_198763	10778620	solute carrier family 1 (glutamate/neutral amino acid transporter), member 4
Slc40a1	1.27	153	0.000		lcn	0.39	NM_133315	10927780	solute carrier family 39 (iron-regulated transporter), member 1
Slc43a3	1.23	29	0.029		tan	0.18	NM_001107743	10837366	solute carrier family 43, member 3
Slco2b1	0.81	23	0.012		brw	2.69	NM_080786	10723902	solute carrier organic anion transporter family, member 2b1
St6gal2	0.77	23	0.038		grn	2.59	AJ627626	10930711	ST6 beta-galactosamide alpha-2,6-sialyltranferase 2
Stard13	1.22	28	0.000		red	1.93	NM_001109060	10756289	StAR-related lipid transfer (START) domain containing 13
Stard3	1.24	45	0.000		slm	2.42	NM_001014229	10737957	StAR-related lipid transfer (START) domain containing 3
Sulf1	0.78	183	0.004		grlw	3.06	NM_134378	10874981	sulfatase 1
Sult1a1	0.78	35	0.003		trq	4.84	NM_031834	10725782	sulfotransferase family, cytosolic, 1A, phenol-preferring, member 1
Tgm2	0.67	150	0.003		grn	1.72	NM_019386	10851350	transglutaminase 2, C polypeptide
LOC500959	1.26	177	0.006		prp	3.43	NM_001033072	10915837	triosephosphate isomerase
Tpi1	1.33	396	0.000		prp	6.43	NM_022922	10798021	triosephosphate isomerase 1
Tpi1	1.27	221	0.039		prp	4.77	NM_022922	10865527	triosephosphate isomerase 1
B3galt1	0.42	39	0.022		gr	0.00	NM_001108954	10836515	UDP-Gal:betaGlcNAc beta 1,3-galactosyltransferase, polypeptide 1
Ust	1.21	52	0.009		red	0.72	NM_001108458	10716667	uronyl-2-sulfotransferase
Vnn1	1.36	70	0.016		ylw	2.46	NM_001025623	10717295	vanin 1
Vat1	1.21	114	0.048		slm	0.86	NM_001033683	10747633	vesicle amine transport protein 1 homolog (T californica)
**Proteolysis**									
Adamts17	0.56	85	0.001		trq	10.03	XM_218753	10707862	ADAM metallopeptidase with thrombospondin type 1 motif, 17
Ctsf	1.38	97	0.003		slm	1.76	NM_001034110	10712935	cathepsin F
Ermp1	0.81	148	0.002		gr	0.00	NM_184050	10729610	endoplasmic reticulum metallopeptidase 1
Naaladl2	0.71	14	0.046		cyan	0.28	XM_574908	10822530	N-acetylated alpha-linked acidic dipeptidase-like 2
Pi15	0.50	79	0.001		trq	1.06	NM_001106917	10874903	peptidase inhibitor 15

Plat	1.31	358	0.017		ylw	6.12	NM_013151	10792421	plasminogen activator, tissue
Psmb5	1.23	137	0.002		pink	8.49	NM_001105727	10783599	proteasome (prosome, macropain) subunit, beta type 5
Rnf213	0.74	54	0.005		cyan	4.55	XM_001081768	10739998	ring finger protein 213
Rnf213	0.65	171	0.028		cyan	9.53	XM_001081768	10739988	ring finger protein 213
Rnf213	0.61	134	0.029		cyan	9.22	XM_001081768	10739994	ring finger protein 213
Rnf213	0.68	110	0.032		cyan	8.74	XM_001081768	10739996	ring finger protein 213
Rnf213	0.68	264	0.044		cyan	9.52	XM_001081768	10739982	ring finger protein 213
Rnf213	0.56	140	0.045		cyan	9.49	XM_001081768	10739992	ring finger protein 213
Serpini1	1.58	44	0.012		gr	0.00	NM_053779	10815962	serine (or cysteine) peptidase inhibitor, clade I, member 1
Snrpb	1.24	107	0.005		pink	8.27	NM_134358	10849863	small nuclear ribonucleoprotein polypeptides B and B1
Ube2l6	1.32	67	0.004		pink	3.80	NM_001024755	10837342	ubiquitin-conjugating enzyme E2L 6
Ufc1	1.39	223	0.003		pink	9.12	NM_001003709	10769862	ubiquitin-fold modifier conjugating enzyme 1
Vps13a	0.80	132	0.046		gr	0.00	NM_001100975	10729124	vacuolar protein sorting 13 homolog A (S. cerevisiae)
**Receptors & Binding Proteins**									
Htr1b	0.66	105	0.047		gr	0.00	NM_022225	10918979	5-hydroxytryptamine (serotonin) receptor 1B
Agtr2	0.68	80	0.035		cyan	0.52	NM_012494	10931616	angiotensin II receptor, type 2
Crhr2	0.62	18	0.037		gr	0.00	NM_022714	10862677	corticotropin releasing hormone receptor 2
Esr1	0.77	35	0.015		mgt	2.67	NM_012689	10702689	estrogen receptor 1
Esr1	0.77	72	0.042		mgt	2.94	NM_012689	10702695	estrogen receptor 1
Grm8	0.68	37	0.003		trq	1.11	NM_022202	10861383	glutamate receptor, metabotropic 8
Il13ra2	0.73	273	0.025		blc	1.72	NM_133538	10937292	interleukin 13 receptor, alpha 2
Pgr	0.58	78	0.045		brw	11.18	NM_022847	10907976	progesterone receptor
Procr	1.49	205	0.026		ylw	2.79	NM_001025733	10841416	protein C receptor, endothelial
P2rx2	0.76	15	0.031		trq	7.10	NM_053656	10759383	purinergic receptor P2X, ligand-gated ion channel, 2
Sorl1	0.74	41	0.030		grlw	0.39	XM_217115	10916493	sortilin-related receptor, LDLR class A repeats-containing
Wipf3	0.75	24	0.038		grn	1.24	NM_147211	10855637	WAS/WASL interacting protein family, member 3
Wbp4	0.83	19	0.024		gr	0.00	NM_053766	10785468	WW domain binding protein 4 (formin binding protein 21)
**Signaling**									
Anxa5	1.40	593	0.000		ylw	4.22	NM_013132	10822837	annexin A5
Bccip	0.80	106	0.031		pink	0.94	NM_001108505	10711769	BRCA2 and CDKN1A interacting protein
Dkk3	1.37	125	0.000		prp	0.54	NM_138519	10724967	dickkopf homolog 3 (Xenopus laevis)
Dab1	0.51	41	0.000		trq	6.13	NM_153621	10870429	disabled homolog 1 (Drosophila)
Dok6	0.69	44	0.021		blu	1.33	XM_001063746	10805605	docking protein 6
Ehd3	1.34	43	0.001		ylw	1.50	NM_138890	10888596	EH-domain containing 3
Ehd4	1.25	111	0.000		slm	0.78	NM_139324	10848733	EH-domain containing 4
Gpr22	0.59	77	0.005		lcn	0.26	NM_001106722	10889575	G protein-coupled receptor 22
Gtpbp6	1.21	15	0.000		slm	0.73	NM_001135840	10759416	GTP binding protein 6 (putative)
Itga2	0.78	38	0.031		trq	2.09	AB067445	10821415	integrin, alpha 2
Lgr5	0.66	35	0.022		mgt	0.92	NM_001106784	10902420	leucine rich repeat containing G protein coupled receptor 5
Ppp1r9a	0.75	39	0.015		gr	0.00	NM_053473	10853614	protein phosphatase 1, regulatory (inhibitor) subunit 9A
Rabac1	1.32	191	0.012		pink	9.12	NM_031774	10719722	Rab acceptor 1 (prenylated)

Rab35	1.25	70	0.014		slm	1.38	NM_001013046	10762593	RAB35, member RAS oncogene family
Stk11ip	1.20	30	0.000		slm	1.77	NM_001106922	10924596	serine/threonine kinase 11 interacting protein
Sipa1l3	0.81	24	0.017		trq	6.82	NM_001013066	10720489	signal-induced proliferation-associated 1 like 3
Scube1	0.56	104	0.004		mgt	5.12	NM_001134884	10905843	signal peptide, CUB domain, EGF-like 1
RGD1562220	0.62	141	0.002		mgt	1.36	XM_001068657	10771492	similar to GPI-gamma 4; GPIgamma4
Smoc2	0.77	58	0.012		brw	1.71	NM_001106215	10703310	SPARC related modular calcium binding 2
Stra6	0.52	77	0.024		mgt	4.42	NM_001029924	10910431	stimulated by retinoic acid gene 6
Trib1	1.35	49	0.000		ylw	3.14	NM_023985	10896793	tribbles homolog 1 (Drosophila)
**Transcription**									
Bmyc	1.20	42	0.033		slm	0.83	NM_001013163	10834360	brain expressed myelocytomatosis oncogene
Cry1	0.81	49	0.034		pink	3.92	NM_198750	10894525	cryptochrome 1 (photolyase-like)
Ehf	0.70	25	0.009		tan	1.68	NM_001106493	10847806	ets homologous factor
Etv1	1.51	102	0.049		ylw	2.04	NM_001108709	10884339	ets variant 1
Fam84b	0.80	58	0.000		gr	0.00	XM_235349	10903979	family with sequence similarity 84, member B
Fam84b	0.78	109	0.026		gr	0.00	XM_001065527	10903977	family with sequence similarity 84, member B
Gtf2h4	1.21	21	0.025		slm	1.59	NM_212501	10827936	general transcription factor II H, polypeptide 4
Gli1	0.80	34	0.003		brw	5.26	AB073717	10903035	GLI-Kruppel family member GLI1
Klf12	0.76	50	0.033		trq	6.13	NM_001107281	10785590	Kruppel-like factor 12
Nrip1	0.83	100	0.012		ylw	1.17	XM_221724	10752754	nuclear receptor interacting protein 1
Sgef	0.80	22	0.047		trq	1.82	XM_227201	10815663	Src homology 3 domain-containing guanine nucleotide exchange factor
Myocd	0.71	31	0.030		blu	1.15	NM_182667	10743652	transcription factor myocardin
Zc3h10	1.21	23	0.010		slm	0.20	XM_001065797	10899741	zinc finger CCCH type containing 10
Zfp192	0.80	18	0.022		gr	0.00	XM_225349	10795313	zinc finger protein 192
Zfp280b	0.79	103	0.001		pink	2.52	NM_001106384	10832460	zinc finger protein 280b
Zmym3	0.81	77	0.006		gr	0.00	NM_001040155	10938671	zinc finger, MYM-type 3
**Translation & Protein Modification**									
Imp3	1.20	169	0.006		pink	3.11	NM_001108152	10910249	IMP3, U3 small nucleolar ribonucleoprotein, homolog (yeast)
LOC690096	0.82	42	0.015		lcn	0.27	XM_001073240	10774432	similar to ribosomal protein L28
**Miscellaneous & Unknown**									
Ccdc123	0.71	36	0.006		gr	0.00	NM_001127599	10706162	coiled-coil domain containing 123
Crip2	1.39	57	0.003		ylw	1.79	NM_022501	10887615	cysteine-rich protein 2
Fam132a	0.38	302	0.021		mgt	5.91	NM_001108000	10874811	family with sequence similarity 132, member A
Fam96a	1.32	195	0.003		pink	9.33	NM_001008327	10911042	family with sequence similarity 96, member A
RGD1307119	0.72	27	0.017		mgt	3.49	NM_001013975	10749728	hypothetical LOC303743
Plbd1	0.73	29	0.015		brw	4.27	NM_001013927	10866459	phospholipase B domain containing 1
Rftn1	1.52	49	0.032		ylw	2.13	NM_001135011	10921343	raftlin lipid raft linker 1
Sdccag1	0.83	77	0.028		pink	1.62	BC089999	10890274	serologically defined colon cancer antigen 1
MGC93975	1.31	98	0.019		slm	1.69	BC079041	10721350	similar to 2310044H10Rik protein
RGD1308489	0.77	390	0.030		mgt	3.44	XR_007342	10869772	similar to hypothetical protein
RGD1308616	0.76	16	0.003		pink	2.09	XM_342892	10879219	similar to KIAA0467 protein
RGD1308616	0.82	37	0.009		pink	1.87	XM_342892	10879225	similar to KIAA0467 protein
RGD1310448	0.82	85	0.033		blu	4.29	XM_230493	10839058	similar to mKIAA1300

									protein
RGD1559695	0.73	14	0.018		gr	0.00	XM_576290	10904460	similar to FLJ43860 protein
RGD1560155	0.79	33	0.023		blc	0.52	NM_001107360	10799187	similar to mKIAA0934 protein
RGD1560224	0.60	33	0.039		mgt	3.35	XM_580090	10882514	RGD1560224
RGD1563065	0.60	34	0.001		gr	0.00	XM_223426	10772788	similar to 3110047P20Rik protein
RGD1563065	0.79	24	0.017		gr	0.00	XM_001078520	10772784	similar to 3110047P20Rik protein
RGD1564943	0.81	33	0.044		gr	0.00	NM_001134628	10803505	similar to 4930429A08Rik protein
RGD1565493	0.71	14	0.043		gr	0.00	XR_007761	10903288	similar to DKFZP434I092 protein
Veph1	0.78	12	0.000		gr	0.00	NM_001014171	10823524	ventricular zone expressed PH domain homolog 1 (zebrafish)
Vgll3	1.21	14	0.041		mgt	0.72	XM_573239	10749839	vestigial like 3 (Drosophila)
Wfikkn2	0.81	11	0.007		cyan	0.09	XM_220855	10746283	WAP, follistatin/kazal, immunoglobulin, kunitz and netrin domain containing 2
Wwc2	0.78	91	0.045		mgt	4.83	NM_001109111	10788101	WW and C2 domain containing 2
**EST's**									
RGD1306682	1.25	34	0.002		pink	3.31	NM_001108296	10746901	similar to RIKEN cDNA 1810046J19
RGD1310660	1.27	146	0.011		pink	3.75	BC086948	10842964	similar to RIKEN cDNA 2700038C09
MGC94207	1.21	25	0.003		blc	1.15	BC081992	10905124	similar to RIKEN cDNA C030006K11
RGD1307220	0.59	39	0.019		gr	0.00	XM_236595	10912819	similar to RIKEN cDNA E330026B02
	1.30	90	0.000		grn	1.31	33314147	10866986	
	0.72	46	0.016		gr	0.00	71043739, 67678358	10931243	
	0.80	41	0.030		grn	1.57	BC166431	10806514	Rattus norvegicus cDNA clone IMAGE:7132976.
	0.82	59	0.013		cyan	0.37	ENSRNOT00000006877	10883445	
	0.83	10	0.016		gr	0.00	ENSRNOT00000011676	10835339	
	0.70	12	0.026		trq	3.43	ENSRNOT00000017435	10838240	
	1.29	13	0.016		lcn	0.54	ENSRNOT00000028053	10747550	
	0.78	29	0.014		red	0.70	ENSRNOT00000028844	10839970	
	0.81	59	0.016		blc	0.53	ENSRNOT00000031724	10872098	
	0.83	22	0.000		gr	0.00	ENSRNOT00000033315	10776960	
	0.79	60	0.021		pink	1.88	ENSRNOT00000033372	10804772	
	0.80	12	0.021		trq	2.14	ENSRNOT00000034326	10856011	
	0.82	21	0.043		trq	8.33	ENSRNOT00000038929	10720516	
	0.78	10	0.017		grn	1.15	ENSRNOT00000039667	10788037	
	0.70	169	0.001		slm	0.58	ENSRNOT00000042139	10710152	
	0.78	18	0.019		pink	0.41	ENSRNOT00000044065	10775318	
	0.47	59	0.034		blc	1.71	ENSRNOT00000045731	10787803	
	0.60	60	0.001		blc	1.13	ENSRNOT00000051386	10878386	
	0.77	30	0.030		lcn	0.45	ENSRNOT00000051623	10708649	
	1.59	51	0.017		ylw	1.58	ENSRNOT00000051834	10716288	
	0.77	11	0.034		slm	0.54	ENSRNOT0000	10880359	

							0053343		
	0.78	24	0.041		grn	0.59	ENSRNOT0000 0053925	10755670	
	0.72	13	0.009		gr	0.00	ENSRNOT0000 0053940	10723464	
	0.82	114	0.047		gr	0.00	ENSRNOT0000 0053987	10755127	
	0.70	67	0.000		pink	0.98	ENSRNOT0000 0054734	10715250	
	0.68	587	0.027		pink	3.41	ENSRNOT0000 0054799	10714204	
	0.81	27	0.042		blc	6.55	ENSRNOT0000 0055033	10725567	
	0.58	18	0.006		tan	0.22	ENSRNOT0000 0055218	10724341	
	0.81	11	0.010		mgt	2.70	ENSRNOT0000 0055292	10907657	
	0.77	15	0.003		gr	0.00	ENSRNOT0000 0055695	10873875	
	0.67	180	0.009		pink	2.77	ENSRNOT0000 0056466	10824788	
	1.28	22	0.026		blc	1.58	ENSRNOT0000 0056643	10939888	
	0.59	74	0.019		pink	1.06	ENSRNOT0000 0056786	10939699	
	0.80	26	0.025		ylw	0.70	ENSRNOT0000 0057318	10934792	
	0.76	63	0.039		brw	12.51	ENSRNOT0000 0058046	10863213	
	1.29	14	0.012		blc	2.59	ENSRNOT0000 0058123	10902762	
	0.70	128	0.005		pink	1.47	ENSRNOT0000 0059259	10802189	
	0.69	26	0.033		pink	0.81	ENSRNOT0000 0060601	10861211	
	0.64	183	0.012		pink	3.52	ENSRNOT0000 0061843	10921268	
	0.64	364	0.036		cyan	9.05	GENSCAN0000 0011985	10739986	
	0.75	36	0.007		gr	0.00	GENSCAN0000 0040452	10718433	
	0.55	671	0.012		lcn	4.99	NC_001665	10930555	
	0.70	268	0.004		lcn	3.19	NC_001665	10930569	
	0.64	265	0.032		lcn	1.74	NC_001665	10930588	
	0.67	1577	0.000		gr	0.00	NC_001665	10930593	
	0.67	1359	0.004		lcn	3.08	NC_001665	10930595	
	0.53	522	0.006		lcn	3.46	NC_001665	10930602	
	0.65	392	0.012		lcn	5.08	NC_001665	10930604	
	0.61	264	0.009		lcn	5.00	NC_001665	10930606	
	0.68	313	0.000		lcn	3.66	NC_001665	10930608	
	0.58	68	0.002		lcn	0.25	NC_001665	10930610	
	0.59	325	0.001		lcn	4.37	NC_001665	10930612	
	0.76	1017	0.028		lcn	4.52	NC_001665	10930614	
	0.56	922	0.026		lcn	4.41	NC_001665	10930620	

C. Genes enfluenced by treatment with fibroblast growth factor 7 (FGF7/KGF) - 123 probe sets									
**Apoptosis**									
**Gene Symbol**	**GF/Con Ratio**	**mean_ diff***	**t-test p-value**	**Short 55 list**	**Module ****	**k.in *****	**GenBank/ Transcript ID**	**Probe Set ID**	**Gene Title/Description**
Casp4	0.83	44	0.022		mgt	0.90	NM_053736	10907825	caspase 4, apoptosis-related cysteine peptidase
Traf3	0.81	28	0.017		trq	17.00	NM_001108724	10887270	Tnf receptor-associated factor 3
**Cell Cycle**									
Ccnf	1.22	34	0.000		slm	0.58	NM_001100474	10741028	cyclin F
Cdt1	1.29	42	0.025		slm	1.22	NM_001106192	10808536	chromatin licensing and DNA replication factor 1
**Cytoskeleton & ECM**									
Cntnap5b	0.66	15	0.025		mgt	2.12	NM_001047873	10763203	contactin associated protein-like 5B
Clec2d	1.24	101	0.009		brw	2.29	NM_130402	10859080	C-type lectin domain family 2, member d
Emp2	1.31	142	0.016		brw	3.58	NM_001007721	10731531	epithelial membrane protein 2
Ecm1	1.25	118	0.004		slm	1.21	NM_053882	10825022	extracellular matrix protein 1
Mylk3	0.76	17	0.008		trq	4.90	NM_001110810	10806461	myosin light chain kinase 3
Parvb	0.79	43	0.020		trq	16.50	NM_001134780	10898229	parvin, beta
Prph	0.69	81	0.035		mgt	3.71	NM_012633	10899061	peripherin
Pcdh7	1.20	25	0.001		blu	2.11	NM_001004087	10776978	protocadherin 7
RGD1563615	1.47	71	0.000		trq	5.65	XM_001064593	10793431	similar to Contactin associated protein-like 3 precursor (Cell recognition molecule Caspr3)
RGD1563615	1.27	60	0.026		trq	6.49	XM_001064593	10793444	similar to Contactin associated protein-like 3 precursor (Cell recognition molecule Caspr3)
RGD1563615	1.26	49	0.029		blu	4.88	XM_001064593	10793433	similar to Contactin associated protein-like 3 precursor (Cell recognition molecule Caspr3)
LOC290704	0.78	65	0.016		trq	8.27	XM_001053156	10787921	similar to palladin
LOC290704	0.75	168	0.029		trq	10.43	XM_001053156	10787925	similar to palladin
Tnnc1	0.83	57	0.040		red	2.28	NM_001034105	10786640	troponin C type 1 (slow)
**Development**									
Amot	0.81	32	0.005		ylw	13.11	XM_235733	10932704	angiomotin
Btbd3	1.27	202	0.006		blu	4.86	NM_001107782	10840262	BTB (POZ) domain containing 3
Emx2	0.77	42	0.035	+	trq	27.47	NM_001109169	10716454	empty spiracles homeobox 2
Hormad1	0.82	86	0.039		red	2.12	NM_001108949	10817438	HORMA domain containing 1
Mmd	0.82	86	0.009		ylw	13.12	NM_001007673	10737429	monocyte to macrophage differentiation-associated
Phex	1.25	92	0.028		trq	6.24	NM_013004	10933664	phosphate regulating endopeptidase homolog, X-linked
Sspn	1.28	41	0.000		prp	1.05	NM_001109255	10859641	sarcospan
Sdk2	0.81	21	0.018		red	2.12	NM_001108303	10748802	sidekick homolog 2 (chicken)
Slmo1	1.28	20	0.033		brw	4.81	NM_001109570	10802427	slowmo homolog 1 (Drosophila)
Tex12	0.78	29	0.017		gr	0.00	XM_001074312	10917231	testis expressed 12
Trpc3	0.65	71	0.004		trq	2.70	NM_021771	10822888	transient receptor potential cation channel, subfamily C, member 3
Zpbp	0.76	64	0.026		trq	15.49	NM_001025139	10778472	zona pellucida binding protein
**Electron Transport**									
Cyct	0.77	26	0.040		gr	0.00	NM_012840	10846324	cytochrome c, testis
Gpx3	0.82	17	0.007		gr	0.00	NM_022525	10733680	glutathione peroxidase 3
**Golgi Apparatus**									

Gcnt2	0.72	48	0.048		trq	5.21	NM_001001511	10797929	glucosaminyl (N-acetyl) transferase 2, I-branching enzyme
LOC685402	0.71	41	0.011		trq	15.69	XM_001063652	10925678	similar to galactose-3-O-sulfotransferase 2
B4galt6	0.81	57	0.045	+	blu	11.67	NM_031740	10803394	UDP-Gal:betaGlcNAc beta 1,4-galactosyltransferase, polypeptide 6
**Growth Factors, Cyto-, and Chemokines**									
Ctgf	1.36	86	0.011	+	blu	8.18	NM_022266	10717233	connective tissue growth factor
Fgf12	0.78	15	0.028		trq	13.17	NM_130814	10751871	fibroblast growth factor 12
Il16	0.74	132	0.036	+	trq	31.93	NM_001105749	10723351	interleukin 16
Megf6	0.76	45	0.021		trq	20.73	NM_022955	10874458	multiple EGF-like-domains 6
Pdgfa	0.78	37	0.014	+	trq	27.00	NM_012801	10757129	platelet-derived growth factor alpha polypeptide
Socs2	1.28	89	0.022		blu	3.68	NM_058208	10901996	suppressor of cytokine signaling 2
Tgfb3	0.83	95	0.025		trq	13.23	NM_013174	10891303	transforming growth factor, beta 3
Tgfbi	0.82	132	0.023		ylw	3.00	XM_001067964	10797138	transforming growth factor, beta induced
**Immune Response**									
Cd3g	0.83	33	0.026		gr	0.00	NM_001077646	10916946	CD3 molecule, gamma polypeptide
Hmcn1	0.82	97	0.009		ylw	3.74	XM_222716	10768426	hemicentin 1
RT1-N2	1.21	56	0.046		gr	0.00	NM_001008854	10827782	RT1 class Ib gene, H2-TL-like, grc region(N2)
**Metabolism & Transport**									
Acpp	0.75	16	0.025		ylw	3.70	NM_020072	10919775	acid phosphatase, prostate
Akr1b8	0.64	79	0.037		ylw	2.67	NM_173136	10854406	aldo-keto reductase family 1, member B8
Akr1c19	1.31	85	0.012		blu	2.88	NM_001100576	10796050	aldo-keto reductase family 1, member C19
Atp1b1	0.79	175	0.035		blu	1.70	NM_013113	10769476	ATPase, Na+/K+ transporting, beta 1 polypeptide
Clic5	0.78	36	0.038		ylw	6.22	NM_053603	10926642	chloride intracellular channel 5
Cmc1	0.82	27	0.040		blu	3.19	NM_001135259	10920797	COX assembly mitochondrial protein homolog (S. cerevisiae)
Rdhe2	0.78	33	0.014		trq	5.95	NM_001106634	10875287	epidermal retinal dehydrogenase 2
Lrp1b	0.81	12	0.025		gr	0.00	NM_001107843	10844962	low density lipoprotein-related protein 1B (deleted in tumors)
Pitpnm2	0.80	23	0.001		trq	8.55	NM_001107139	10758302	phosphatidylinositol transfer protein, membrane-associated 2
Podxl	0.78	225	0.024	+	trq	26.60	NM_138848	10861662	podocalyxin-like
Kctd1	1.32	20	0.019		slm	0.25	AF473845	10803294	potassium channel tetramerisation domain containing 1
Slc4a4	0.76	152	0.014	+	trq	28.98	NM_053424	10775997	solute carrier family 4 (anion exchanger), member 4
Slco1a5	0.79	13	0.009		mgt	3.78	NM_030838	10866606	solute carrier organic anion transporter family, member 1a5
St3gal1	0.82	100	0.019		trq	3.04	NM_001013219	10904189	ST3 beta-galactoside alpha-2,3-sialyltransferase 1
Tgm2	1.25	113	0.044		grn	1.72	NM_019386	10851350	transglutaminase 2, C polypeptide
**Proteolysis**									
Adam33	0.76	42	0.045		ylw	4.61	NM_001107776	10850015	ADAM metallopeptidase domain 33
Hecw2	1.24	40	0.021		blu	9.48	NM_001108218	10928001	HECT, C2 and WW domain containing E3 ubiquitin protein ligase 2
Mmp13	0.43	47	0.041		trq	1.56	NM_133530	10907858	matrix metallopeptidase 13

Ubxn2b	1.21	125	0.024		blu	8.99	NM_001107905	10867505	UBX domain protein 2B
**Receptors & Binding Proteins**									
Boc	0.83	100	0.023		trq	10.82	NM_001108317	10751115	biregional cell adhesion molecule-related/down-regulated by oncogenes (Cdon) binding protein
Bmpr1b	1.26	112	0.034		blu	6.09	NM_001024259	10827068	bone morphogenetic protein receptor, type IB
Fgfr2	0.79	296	0.010	+	trq	30.76	NM_012712	10726172	fibroblast growth factor receptor 2
Fshr	1.32	59	0.039		red	3.54	NM_199237	10888354	follicle stimulating hormone receptor
Grm8	0.68	37	0.010		trq	1.11	NM_022202	10861383	glutamate receptor, metabotropic 8
Gnrhr	1.34	12	0.041		brw	6.55	NM_031038	10772066	gonadotropin releasing hormone receptor
Plxna4a	0.75	128	0.040	+	trq	30.89	NM_001107852	10861678	plexin A4, A
Pgr	1.25	48	0.017		brw	11.18	NM_022847	10907976	progesterone receptor
Scarb1	0.82	63	0.028		mgt	3.27	NM_031541	10758137	scavenger receptor class B, member 1
Sobpl	0.78	23	0.031		trq	10.47	NM_001104640	10833823	sine oculis-binding protein homolog-like (Drosophila)
Vom2r16	0.67	32	0.018		trq	5.96	NM_001099655	10703593	vomeronasal 2 receptor, 16
**Signaling**									
Cdc42ep3	0.80	55	0.050		trq	2.28	NM_001048044	10887939	CDC42 effector protein (Rho GTPase binding) 3
Cdgap	1.27	74	0.044		brw	5.84	NM_001105879	10751255	Cdc42 GTPase-activating protein
Dennd1a	0.82	94	0.041		trq	21.96	XM_231184	10844827	DENN/MADD domain containing 1A
Entpd3	0.75	31	0.015		trq	15.81	NM_178106	10914354	ectonucleoside triphosphate diphosphohydrolase 3
Frmd3	1.22	29	0.001		ylw	0.57	NM_001106662	10869574	FERM domain containing 3
Frmd5	1.36	104	0.024		blu	4.74	XM_230513	10849156	FERM domain containing 5
Grid2	0.82	14	0.043		grn	0.33	NM_024379	10855950	glutamate receptor, ionotropic, delta 2
Map3k1	0.77	158	0.024	+	trq	26.84	NM_053887	10821276	mitogen activated protein kinase kinase kinase 1
Mobkl2b	1.22	40	0.044		trq	1.47	NM_001108970	10875954	MOB1, Mps One Binder kinase activator-like 2B (yeast)
Nrgn	0.83	12	0.046	+	ylw	16.11	NM_024140	10916228	neurogranin
Pak6	0.74	15	0.042		ylw	11.45	NM_001106498	10838670	p21 protein (Cdc42/Rac)-activated kinase 6
Rabl4	0.79	65	0.003		gr	0.00	NM_001130495	10905270	RAB, member of RAS oncogene family-like 4
Sh3gl2	0.81	13	0.024		gr	0.00	AF009603	10869728	SH3-domain GRB2-like 2
Spry3	0.83	13	0.024		gr	0.00	NM_001109063	10760805	sprouty homolog 3 (Drosophila)
Tbc1d2b	0.76	126	0.033		trq	23.16	NM_001108175	10919283	TBC1 domain family, member 2B
Tmem151a	0.76	52	0.032	+	trq	26.21	NM_001107570	10727725	transmembrane protein 151A
**Transcription**									
Ahr	0.82	46	0.019		ylw	10.79	NM_013149	10889660	aryl hydrocarbon receptor
Etv4	0.73	26	0.011		ylw	13.65	NM_001108299	10747665	ets variant 4
Gtf2a2	1.20	210	0.027		red	2.16	NM_053345	10911309	general transcription factor IIA, 2
Lhx9	0.73	89	0.019		trq	20.54	NM_181367	10768194	LIM homeobox 9
Lhfp	0.82	22	0.004		ylw	7.56	NM_001109183	10815321	lipoma HMGIC fusion partner
Med21	0.79	148	0.020		blu	3.38	NM_001107895	10859655	mediator complex subunit 21
Rfc4	0.80	174	0.046		gr	0.00	NM_001105869	10751973	replication factor C (activator 1) 4
Scx	0.80	12	0.044		ylw	4.03	NM_001130508	10897285	scleraxis
Sertad1	0.81	15	0.013		gr	0.00	NM_001007735	10705364	SERTA domain containing 1
Maf	0.76	100	0.039		trq	18.61	NM_019318	10811278	v-maf musculoaponeurotic fibrosarcoma oncogene

									homolog (avian)
Wt1	0.82	208	0.006		trq	24.71	NM_031534	10838242	Wilms tumor 1
**Miscellaneous & Unknown**									
Aim1	1.22	35	0.032		gr	0.00	XM_001067657	10833873	absent in melanoma 1
Depdc7	0.77	159	0.012		red	4.11	NM_001029916	10847932	DEP domain containing 7
Ift57	0.78	121	0.042		mgt	2.57	NM_001107093	10753784	intraflagellar transport 57 homolog (Chlamydomonas)
RGD1561503	0.82	32	0.038		gr	0.00	XM_574857	10821623	similar to hypothetical protein AN1443.2
RGD1306622	0.70	107	0.038	+	trq	32.56	XM_001074493	10728647	similar to KIAA0954 protein
**EST's**									
MGC105649	0.75	174	0.041		trq	22.31	NM_001008518	10839307	hypothetical LOC302884
RGD1307218	1.21	22	0.004		trq	4.20	BC089972	10876730	similar to RIKEN cDNA 2810432L12
RGD1310827	1.23	60	0.000		slm	0.45	BC100659	10862605	similar to RIKEN cDNA 1200009O22; EST AI316813
RGD1563349	1.25	23	0.044		brw	17.30	NM_001109345	10860566	similar to RIKEN cDNA 9330182L06
	0.81	28	0.043		trq	3.55	21595273	10877888	
	0.73	24	0.004		trq	1.16	38569472, 116496660, 116497146	10894419	
	0.83	30	0.039		grn	1.60	ENSRNOT00000013725	10866265	
	0.68	110	0.017		trq	22.60	ENSRNOT00000031367	10765364	
	1.21	31	0.002		red	0.74	ENSRNOT00000033742	10873809	
	0.73	118	0.001		trq	22.62	ENSRNOT00000035129	10764376	
	0.79	25	0.004		gr	0.00	ENSRNOT00000038075	10891612	
	0.83	70	0.025		trq	15.61	ENSRNOT00000043907	10764388	
	0.82	42	0.025		trq	6.02	ENSRNOT00000047223	10787828	
	0.79	15	0.025		trq	14.01	ENSRNOT00000059999	10889627	
	0.82	12	0.001		gr	0.00	GENSCAN00000036880	10873872	

D. Genes influenced by treatment with bone morphogenetic protein 4 (BMP4) - 79 probe sets									
**Apoptosis**									
**GeneSymbol**	**GF/Con Ratio**	**mean_ diff***	**t-test p-value**	**Short 55 list**	**module ****	**k.in *****	**GenBank**	**ProbeSet_id**	**GeneTitle**
Cd55	0.83	60	0.030		blu	0.85	NM_022269	10767388	Cd55 molecule
Tnfrsf1a	0.83	47	0.016		ylw	9.03	NM_013091	10858967	tumor necrosis factor receptor superfamily, member 1a
**Cytoskeleton & ECM**									
Cntnap1	1.22	49	0.048		red	0.49	NM_032061	10738313	contactin associated protein 1
Krt8	0.76	101	0.023		ylw	4.80	NM_199370	10907524	keratin 8
Krt8	0.80	59	0.033		ylw	4.93	NM_199370	10751352	keratin 8
Lgals3bp	0.75	129	0.031		blu	2.61	NM_139096	10749495	lectin, galactoside-binding, soluble, 3 binding protein
Lgals9	0.82	39	0.050		gr	0.00	NM_012977	10745292	lectin, galactoside-binding, soluble, 9
Prph	1.56	147	0.017		mgt	3.71	NM_012633	10899061	peripherin
Sdc4	0.76	332	0.048		ylw	4.58	NM_012649	10851599	syndecan 4
Zp1	0.69	109	0.005		blu	3.56	NM_053509	10728817	zona pellucida glycoprotein 1 (sperm receptor)
**Development**									
Mgp	0.78	306	0.000		ylw	10.11	NM_012862	10866512	matrix Gla protein
Nedd9	0.80	40	0.003		gr	0.00	NM_001011922	10794599	neural precursor cell expressed, developmentally down-regulated 9
Sema3d	0.75	20	0.019		ylw	3.02	NM_001104633	10860499	sema domain, immunoglobulin domain (Ig), short basic domain, secreted, (semaphorin) 3D
**Epigenetics**									
Gadd45a	0.80	122	0.013		grlw	0.22	NM_024127	10862867	growth arrest and DNA-damage-inducible, alpha
Hist1h2bp	1.24	26	0.029		blu	1.29	XM_225384	10795282	histone cluster 1, H2bp
Tbx3	1.25	16	0.002		ylw	0.26	NM_181638	10762378	T-box 3
**Golgi Apparatus**									
Pfkfb3	0.82	20	0.004		prp	1.51	NM_057135	10796149	6-phosphofructo-2-kinase/fructose-2,6-biphosphatase 3
Galnt1	0.82	191	0.001		blu	0.23	NM_024373	10800497	UDP-N-acetyl-alpha-D-galactosamine:polypeptide N-acetylgalactosaminyltransferase 1 (GalNAc-T1)
**Growth Factors, Cyto-, and Chemokines**									
Bmp3	0.75	12	0.027		blu	0.48	NM_017105	10775573	bone morphogenetic protein 3
Cxcl10	0.67	34	0.038		gr	0.00	NM_139089	10771655	chemokine (C-X-C motif) ligand 10
Cxcl13	0.71	133	0.004		ylw	14.01	NM_001017496	10775731	chemokine (C-X-C motif) ligand 13
**Immune Response**									
Cd68	1.26	57	0.003		trq	1.91	NM_001031638	10744127	Cd68 molecule
C1r	0.79	301	0.039		ylw	7.83	NM_001134555	10858655	complement component 1, r subcomponent
C1s	0.74	261	0.013		ylw	7.70	NM_138900	10865442	complement component 1, s subcomponent
H2-T23	0.72	30	0.021		gr	0.00	NM_001008886	10827809	histocompatibility 2, T region locus 23
**Metabolism & Transport**									
Abat	0.81	15	0.036		blu	1.50	NM_031003	10740529	4-aminobutyrate aminotransferase
Acss2	0.78	51	0.001		red	0.99	NM_001107793	10841348	acyl-CoA synthetase short-chain family member 2
Ddhd1	0.79	70	0.018		trq	1.75	NM_001033066	10782870	DDHD domain containing 1
Elovl7	0.81	80	0.027		blu	1.02	XM_342192	10812922	ELOVL family member 7, elongation of long chain fatty acids (yeast)

Ero1l	0.80	81	0.010		prp	6.02	NM_138528	10782826	ERO1-like (S. cerevisiae)
Hsd11b1	0.75	119	0.018	+	ylw	17.04	NM_017080	10770795	hydroxysteroid 11-beta dehydrogenase 1
Isyna1	0.81	307	0.035		grlw	1.00	NM_001013880	10790966	inositol-3-phosphate synthase 1
UST4r	1.24	13	0.014		gr	0.00	NM_134379	10728466	integral membrane transport protein UST4r
Nans	0.83	25	0.048		ylw	1.80	NM_001106655	10868864	N-acetylneuraminic acid synthase
Pnpla1	0.81	24	0.002		grn	2.23	XM_342105	10828805	patatin-like phospholipase domain containing 1
Pgk1	0.83	142	0.038		prp	8.61	NM_053291	10934610	phosphoglycerate kinase 1
Pygl	0.76	43	0.046		prp	4.55	NM_022268	10890441	phosphorylase, glycogen, liver
Plod1	0.81	63	0.000		prp	5.93	NM_053827	10881521	procollagen-lysine 1, 2-oxoglutarate 5-dioxygenase 1
Prr16	0.82	10	0.014		ylw	8.61	NM_001108432	10801683	proline rich 16
Retsat	0.83	71	0.022		ylw	1.06	NM_145084	10856424	retinol saturase (all trans retinol 13,14 reductase)
RGD1559856	0.67	32	0.031		gr	0.00	XM_225971	10801336	similar to potassium channel tetramerisation domain containing 16
Slc2a1	0.81	94	0.033		prp	6.19	NM_138827	10871521	solute carrier family 2 (facilitated glucose transporter), member 1
Slc6a9	0.83	67	0.041		blu	0.71	NM_053818	10871444	solute carrier family 6 (neurotransmitter transporter, glycine), member 9
Ust	0.81	47	0.000		red	0.72	NM_001108458	10716667	uronyl-2-sulfotransferase
**Proteolysis**									
Adamts9	0.77	34	0.005		ylw	5.49	NM_001107877	10864185	a disintegrin-like and metalloprotease (reprolysin type) with thrombospondin type 1 motif, 9
Serping1	0.82	221	0.012		ylw	3.21	NM_199093	10846854	serine (or cysteine) peptidase inhibitor, clade G, member 1
Timp1	0.76	373	0.032		grn	8.35	NM_053819	10936482	TIMP metallopeptidase inhibitor 1
Usp18	0.65	41	0.029		gr	0.00	NM_001014058	10858370	ubiquitin specific peptidase 18
**Receptors & Binding Proteins**									
Axl	0.83	112	0.008	+	ylw	15.39	NM_031794	10719900	Axl receptor tyrosine kinase
Gphn	0.82	99	0.020		trq	5.17	NM_022865	10885484	gephyrin
Igfbp5	1.21	1226	0.000		mgt	3.74	NM_012817	10928837	insulin-like growth factor binding protein 5
Il1r1	0.80	97	0.043		ylw	13.96	NM_013123	10922826	interleukin 1 receptor, type I
Il17rd	0.80	68	0.039		blu	1.40	XM_573851	10786324	interleukin 17 receptor D
Ptx3	0.72	30	0.001		brw	3.59	NM_001109536	10815785	pentraxin related gene
Rtp4	0.68	46	0.028		gr	0.00	NM_001108321	10755088	receptor (chemosensory) transporter protein 4
**Signaling**									
Dusp6	0.77	71	0.021	+	ylw	16.58	NM_053883	10895144	dual specificity phosphatase 6
Lcn2	0.71	191	0.041		ylw	3.42	NM_130741	10844331	lipocalin 2
Rassf4	1.24	21	0.003		grn	0.66	NM_001024275	10864874	Ras association (RalGDS/AF-6) domain family member 4
Smad6	1.49	173	0.007		mgt	6.12	NM_001109002	10918186	SMAD family member 6
Smad7	1.38	42	0.045		mgt	4.73	NM_030858	10802734	SMAD family member 7
Pef1	1.26	34	0.028		mgt	0.84	NM_001007651	10901111	penta-EF hand domain containing 1
**Transcription**									
Ehf	0.75	20	0.050		tan	1.68	NM_001106493	10847806	ets homologous factor
Hif3a	1.21	22	0.023		cyan	0.41	NM_022528	10719323	hypoxia inducible factor 3, alpha subunit
Id1	1.23	131	0.001		pink	0.38	NM_012797	10840890	inhibitor of DNA binding 1
RGD1562462	0.72	12	0.017		gr	0.00	XM_222950	10770109	similar to Ifi204 protein
Tbpl2	0.81	18	0.045		grn	1.66	NM_001099361	10843193	TATA box binding protein-

									like 2
**Translation & Protein Modification**									
Oas1b	0.69	13	0.040		gr	0.00	NM_144752	10762247	2-5 oligoadenylate synthetase 1B
Oasl2	0.66	33	0.016		gr	0.00	NM_001009682	10762747	2'-5' oligoadenylate synthetase-like 2
Slfn2	0.79	28	0.005		gr	0.00	NM_001107031	10736795	schlafen 2
**Miscellaneous & Unknown**									
RGD1310271	1.28	39	0.037		mgt	3.95	XM_233727	10882356	similar to hypothetical protein MGC45873
Trim7	0.77	13	0.011		lcn	0.40	XM_220359	10733049	tripartite motif-containing 7
**EST**									
	0.81	14	0.029		gr	0.00	ENSRNOT00000030546	10766918	
	0.83	34	0.032		prp	3.71	ENSRNOT00000038163	10901036	
	0.69	11	0.019		gr	0.00	ENSRNOT00000039932	10923212	
	0.82	10	0.023		mgt	0.46	ENSRNOT00000048364	10900112	
	0.83	90	0.018		gr	0.00	ENSRNOT00000048912	10787048	
	0.79	28	0.012		gr	0.00	ENSRNOT00000052816	10713606	
	0.73	61	0.007		pink	0.98	ENSRNOT00000054734	10715250	
	1.21	82	0.042		trq	3.03	ENSRNOT00000059997	10909377	

E. Genes influenced by treatment with glial cell derived neurotrophic factor (GDNF) - 148 probe sets									
**Apoptosis**									
**Gene Symbol**	**GF/Con Ratio**	**mean_ diff***	**t-test p-value**	**Short 55 list**	**Module ****	**k.in *****	**GenBank/ Transcript ID**	**Probe Set ID**	**Gene Title/Description**
Traf3ip2	0.81	25	0.039		trq	6.56	NM_001044248	10830398	Traf3 interacting protein 2
**Cytoskeleton & ECM**									
Blnk	0.82	16	0.043		blu	0.43	NM_001025767	10730131	B-cell linker
Cntnap5b	0.68	14	0.043		mgt	2.12	NM_001047873	10763203	contactin associated protein-like 5B
LOC501599	1.38	21	0.020		blc	0.49	XR_007687	10938981	similar to galectin 8
Tppp3	0.80	10	0.034		tan	0.44	NM_001009639	10810631	tubulin polymerization-promoting protein family member 3
**Development**									
Crispld1	0.82	22	0.022		blu	3.13	NM_001134963	10874887	cysteine-rich secretory protein LCCL domain containing 1
Nsg1	0.79	19	0.010		blu	0.83	NM_024128	10773247	neuron specific gene family member 1
Ntn2l	0.82	14	0.026		grn	0.46	AY028418	10741009	netrin 2-like (chicken)
RGD1564739	1.24	45	0.004		blu	3.08	XR_008648	10809882	similar to spermatogenesis associated glutamate (E)-rich protein 4d
Sema3e	0.81	11	0.048		trq	13.02	NM_001106579	10860457	sema domain, immunoglobulin domain (Ig), short basic domain, secreted, (semaphorin) 3E
Usmg5	0.81	129	0.023	+	blu	13.50	NM_133544	10730633	up-regulated during skeletal muscle growth 5 homolog (mouse)
Usmg5	0.82	112	0.031	+	blu	12.56	NM_133544	10747614	up-regulated during skeletal muscle growth 5 homolog (mouse)
Zfhx2	1.33	17	0.015		blc	1.52	NM_001098803	10783753	zinc finger homeobox 2
**Electron Transport**									
Cox7b	0.77	64	0.038		blu	5.73	NM_182819	10934579	cytochrome c oxidase subunit VIIb
Cox7c	0.77	321	0.024		blu	5.55	NM_001134705	10820277	cytochrome c oxidase, subunit VIIc
Tmem126a	0.78	25	0.037		gr	0.00	NM_001011557	10723639	transmembrane protein 126A
**Epigenetics**									
LOC680511	0.78	18	0.035		blu	3.41	XM_001061523	10795289	similar to histone 1, H2ai
LOC690102	0.78	51	0.002		blu	1.22	NM_001111341	10817527	similar to histone H2A
**Golgi Apparatus**									
B3galnt1	0.83	24	0.024		blu	2.68	NM_001013158	10823605	beta-1,3-N-acetylgalactosaminyltransferase 1
Man1a1	0.80	82	0.043		trq	18.24	NM_001033656	10833394	mannosidase, alpha, class 1A, member 1
Nans	0.83	25	0.017		ylw	1.80	NM_001106655	10868864	N-acetylneuraminic acid synthase
**Growth Factors, Cyto-, and Chemokines**									
Mdk	0.81	102	0.031		blc	0.36	NM_030859	10847432	midkine
Sdf2	0.82	20	0.031		blu	4.44	NM_001105803	10745185	stromal cell derived factor 2
**Immune Response**									
C7	1.29	155	0.023		grlw	0.46	AF309948	10821666	complement component 7
Hla-dmb	0.70	34	0.033		mgt	3.03	NM_198740	10831620	major histocompatibility complex, class II, DM beta
**Metabolism**									
Atp5i	0.81	60	0.001		gr	0.00	NM_080481	10770996	ATP synthase, H+ transporting, mitochondrial F0 complex, subunit E
Ndufa1	0.82	51	0.037		gr	0.00	NM_001108813	10931991	NADH dehydrogenase (ubiquinone) 1 alpha subcomplex, 1
Pld6	1.20	30	0.001		gr	0.00	XM_220517	10743168	phospholipase D family, member 6

Selm	0.78	41	0.028		red	1.27	NM_001115013	10773716	selenoprotein M
LOC362405	1.28	19	0.006		blc	0.57	XR_007425	10911377	similar to Glyceraldehyde-3-phosphate dehydrogenase (GAPDH)
RGD1564921	0.82	136	0.001		blu	9.99	XM_345576	10871582	similar to peptidyl prolyl isomerase H
Sult5a1	0.80	18	0.007		cyan	0.09	NM_001106194	10811732	sulfotransferase family 5A, member 1
Upp1	1.22	11	0.048		ylw	1.94	NM_001030025	10774171	uridine phosphorylase 1
Uros	0.81	13	0.023		gr	0.00	NM_001012068	10726346	uroporphyrinogen III synthase
**Proteolysis**									
Rkhd1	0.82	20	0.022		trq	5.20	XM_234921	10893858	ring finger (C3HC4 type) and KH domain containing 1
**Receptors & Binding Proteins**									
Fabp3	0.79	20	0.031		gr	0.00	NM_024162	10872473	fatty acid binding protein 3, muscle and heart
Fcrls	0.76	30	0.048		mgt	0.16	NM_001107702	10824140	Fc receptor-like S, scavenger receptor
Itgb3bp	0.79	23	0.034	+	blu	10.96	NM_001013213	10878272	integrin beta 3 binding protein (beta3-endonexin)
Lgtnl1	1.37	17	0.017		blc	0.58	XM_001076874	10705032	ligatin-like 1
Olr1226	1.29	14	0.028		blc	0.50	NM_001000442	10909154	olfactory receptor 1226
Olr1448	1.31	34	0.000		blc	0.58	NM_001000019	10733888	olfactory receptor 1448
Olr666	1.28	11	0.002		gr	0.00	NM_001000924	10847144	olfactory receptor 666
P2rx2	0.74	17	0.039		trq	7.10	NM_053656	10759383	purinergic receptor P2X, ligand-gated ion channel, 2
Vom2r6	0.79	50	0.010		mgt	1.29	NM_001099459	10701654	vomeronasal 2 receptor, 6
**Signaling**									
Calml3	0.73	69	0.027		mgt	6.36	NM_001012054	10796090	calmodulin-like 3
Efna5	0.83	17	0.048	+	ylw	15.03	NM_053903	10930204	ephrin A5
Itgb8	0.77	51	0.040		trq	8.58	NM_001108726	10892835	integrin beta 8
Pex6	0.83	13	0.010		trq	0.90	NM_057125	10921677	peroxisomal biogenesis factor 6
Ptprc	0.80	28	0.027		blu	2.29	NM_138507	10768138	protein tyrosine phosphatase, receptor type, C
Rpesp	0.79	15	0.050		blu	6.26	XM_216317	10867102	RPE-spondin
Smad6	0.77	80	0.043		mgt	6.12	NM_001109002	10918186	SMAD family member 6
Smad7	0.75	28	0.012		mgt	4.73	NM_030858	10802734	SMAD family member 7
**Transcription**									
Ddx60	0.81	127	0.031		mgt	3.73	XP_001070139	10791358	DEAD (Asp-Glu-Ala-Asp) box polypeptide 60
Ddx60	0.81	94	0.040		mgt	3.65	XP_001070139	10787889	DEAD (Asp-Glu-Ala-Asp) box polypeptide 60
Ddx60	0.81	93	0.040		mgt	3.52	XP_001070139	10791394	DEAD (Asp-Glu-Ala-Asp) box polypeptide 60
Higd1a	0.79	26	0.002		prp	0.90	NM_080902	10914415	HIG1 hypoxia inducible domain family, member 1A
Klhl25	0.81	19	0.010		gr	0.00	NM_001039006	10707992	kelch-like 25 (Drosophila)
**Translation & Proein Modification**									
RGD1561871	1.27	19	0.007		mbl	1.26	XM_001081056	10895702	similar to ribosomal protein S10
LOC690732	0.75	74	0.003		blu	5.53	XM_001075416	10829660	similar to 40S ribosomal protein S28
Rps28	0.83	236	0.019		gr	0.00	NM_001105730	10774372	ribosomal protein S28
Taf9	0.82	106	0.047		pink	1.30	NM_184048	10832879	TAF9 RNA polymerase II, TATA box binding protein (TBP)-associated factor
**Miscellaneous & Unknown**									
Fam82a	0.83	171	0.041		trq	5.53	BC100261	10882475	family with sequence similarity 82, member A
LOC681251	1.24	12	0.040		blc	0.59	XM_001060948	10805283	hypothetical protein LOC681251
Ift57	0.74	144	0.017		mgt	2.57	NM_001107093	10753784	intraflagellar transport 57 homolog (Chlamydomonas)
RGD1559695	1.24	25	0.049		blc	1.34	XM_576290	10904466	similar to FLJ43860 protein
LOC679583	0.79	72	0.023		blu	4.47	XM_001066513	10871487	similar to UPF0197 protein C11orf10 homolog
LOC686092	0.79	80	0.026		blu	4.25	XM_001066513	10923334	similar to UPF0197 protein C11orf10 homolog

Tmem200a	0.83	13	0.002		mgt	2.02	NM_001109135	10702189	transmembrane protein 200A
**EST's**									
RGD1566265	1.21	90	0.004		blu	4.06	NM_001134589	10939002	similar to RIKEN cDNA 2610002M06
LOC366502	0.80	11	0.039		gr	0.00	XR_009001	10881645	hypothetical gene supported by NM_022949
	0.79	11	0.049		gr	0.00	AF277175	10854284	
	1.28	67	0.000		pink	1.81	12751097	10811859	
	1.40	13	0.022		blc	2.83	13543996	10909581	
	0.82	27	0.037		trq	3.55	21595273	10877888	
	0.79	40	0.001		blu	0.12	37182531	10864272	
	2.06	39	0.016		blc	2.41	55741660	10868796	
	0.83	38	0.047		gr	0.00	91983636	10900106	
	0.78	25	0.040		gr	0.00	38569472, 116496660, 116497146	10891830	
	1.22	87	0.011		gr	0.00	AF090348	10919712	
	0.74	211	0.027		blu	14.42	AF188753	10855185	
	0.70	91	0.028		blu	11.15	AF272707	10867318	
	0.77	48	0.011		blu	7.56	ENSRNOT00000008028	10752515	
	0.83	58	0.032		ylw	7.37	ENSRNOT00000013475	10785895	
	1.32	16	0.001		blc	1.01	ENSRNOT00000018878	10715068	
	0.83	20	0.011		gr	0.00	ENSRNOT00000020374	10910167	
	0.83	50	0.007		blc	0.95	ENSRNOT00000021284	10859627	
	1.45	17	0.014		blc	1.71	ENSRNOT00000023104	10823348	
	0.58	12	0.018		gr	0.00	ENSRNOT00000032512	10905177	
	1.22	87	0.011		gr	0.00	AF090348	10919712	
	0.80	44	0.000		blc	0.84	ENSRNOT00000032933	10705422	
	1.50	13	0.012		blc	0.19	ENSRNOT00000033178	10709786	
	0.79	93	0.008		trq	22.62	ENSRNOT00000035129	10764376	
	0.82	32	0.010		blc	0.62	ENSRNOT00000037416	10863570	
	0.70	36	0.000		gr	0.00	ENSRNOT00000038075	10891612	
	0.76	12	0.039		gr	0.00	ENSRNOT00000040289	10829828	
	0.83	120	0.009		grn	0.87	ENSRNOT00000045444	10820324	
	1.41	12	0.030		blc	0.93	ENSRNOT00000047460	10767915	
	0.81	13	0.034		blu	4.85	ENSRNOT00000047723	10927610	
	1.29	114	0.046		mbl	4.33	ENSRNOT00000048694	10795344	
	1.41	24	0.040		mbl	1.55	ENSRNOT00000049443	10866332	
	0.79	25	0.014		blu	1.08	ENSRNOT00000049673	10821820	
	0.76	13	0.014		blc	0.12	ENSRNOT00000051642	10847426	
	0.78	25	0.017		mgt	1.02	ENSRNOT00000051790	10852187	
	0.71	16	0.028		blu	2.35	ENSRNOT00000052447	10844062	
	1.30	13	0.017		gr	0.00	ENSRNOT00000052457	10779636	
	0.76	108	0.044		blu	12.95	ENSRNOT00000052472	10728561	
	0.76	108	0.044		blu	12.95	ENSRNOT00000052472	10728563	

	0.76	94	0.035		blu	9.12	ENSRNOT0000 0052620	10828077	
	0.69	15	0.048		blu	4.04	ENSRNOT0000 0052658	10782982	
	1.54	20	0.003		blc	1.65	ENSRNOT0000 0052693	10797939	
	0.83	16	0.004		gr	0.00	ENSRNOT0000 0052760	10906870	
	0.67	141	0.028		blu	8.24	ENSRNOT0000 0052995	10723898	
	0.83	42	0.036		blu	3.95	ENSRNOT0000 0053174	10851382	
	0.79	149	0.017		blu	8.50	ENSRNOT0000 0053268	10909356	
	0.79	309	0.034		blu	16.09	ENSRNOT0000 0053402	10721698	
	0.75	24	0.024		blu	14.54	ENSRNOT0000 0053447	10765495	
	0.72	78	0.045		blu	11.53	ENSRNOT0000 0053476	10702293	
	0.71	80	0.047		blu	11.98	ENSRNOT0000 0053479	10851380	
	0.65	149	0.031		blu	14.49	ENSRNOT0000 0053497	10878965	
	0.74	180	0.030		blu	4.82	ENSRNOT0000 0053552	10713608	
	0.73	99	0.010		blu	9.91	ENSRNOT0000 0053807	10909360	
	1.33	13	0.034		blc	1.47	ENSRNOT0000 0053877	10901954	
	1.24	11	0.013		gr	0.00	ENSRNOT0000 0053884	10804339	
	0.60	170	0.018		blu	14.58	ENSRNOT0000 0053929	10839872	
	0.83	108	0.040		gr	0.00	ENSRNOT0000 0053987	10755127	
	0.77	100	0.024		blu	12.13	ENSRNOT0000 0054102	10723896	
	0.78	64	0.016		blu	1.22	ENSRNOT0000 0054185	10721696	
	0.81	18	0.022		lcn	0.08	ENSRNOT0000 0054464	10759483	
	1.38	299	0.011		blc	0.70	ENSRNOT0000 0054563	10866957	
	1.47	94	0.004		blc	2.36	ENSRNOT0000 0054573	10860325	
	0.76	56	0.015		mgt	1.40	ENSRNOT0000 0054641	10911346	
	0.64	15	0.050		tan	0.22	ENSRNOT0000 0055218	10724341	
	0.70	28	0.049		trq	17.02	ENSRNOT0000 0055743	10873814	
	1.27	34	0.037		ylw	0.70	ENSRNOT0000 0057318	10934792	
	0.81	19	0.014		brw	6.77	ENSRNOT0000 0057883	10837170	
	0.72	13	0.005		mgt	1.03	ENSRNOT0000 0058457	10938160	
	1.54	82	0.025		blc	2.11	ENSRNOT0000 0058693	10781288	
	1.85	54	0.033		blc	2.40	ENSRNOT0000 0059520, ENSRNOT0000 0059519, ENSRNOT0000 0059521	10772514	
	0.82	15	0.011		pink	0.81	ENSRNOT0000 0060601	10861211	
	1.45	26	0.042		blc	2.37	ENSRNOT0000 0060749	10908655	
	0.69	56	0.025		blu	13.55	ENSRNOT0000 0062861	10702192	

	0.69	56	0.025		blu	13.55	ENSRNOT0000 0062861	10739209	
	0.69	56	0.025		blu	13.55	ENSRNOT0000 0062861	10788077	
	0.69	56	0.025		blu	13.55	ENSRNOT0000 0062861	10863402	
	0.69	56	0.025		blu	13.55	ENSRNOT0000 0062861	10885957	
	0.69	56	0.025		blu	13.55	ENSRNOT0000 0062861	10922249	
	0.69	56	0.025		blu	13.55	ENSRNOT0000 0062861	10937321	
	0.65	38	0.042		blu	11.39	J01884	10830454	
	1.27	46	0.045		mbl	0.98	rno-mir-383	10788451	MI0003478 Rattus norvegicus miR-383 stem-loop

F. Genes influenced by treatment with KIT ligand (KITLG) - 271 probe sets									
**Apoptosis**									
**Gene Symbol**	**GF/Con Ratio**	**mean_ diff***	**t-test p-value**	**Short 55 list**	**Module ****	**k.in *****	**GenBank/ Transcript ID**	**Probe Set ID**	**Gene Title/Description**
Bcl2l10	0.44	131	0.008	+	red	10.05	NM_053733	10911690	BCL2-like 10 (apoptosis facilitator)
Birc3	0.74	36	0.002		brw	9.30	NM_023987	10914799	baculoviral IAP repeat-containing 3
Nlrp4a	0.66	31	0.005		brw	10.69	NM_001106220	10718621	NLR family, pyrin domain containing 4A
Nlrp4b	0.49	46	0.031		brw	7.45	XM_344856	10719038	NLR family, pyrin domain containing 4B
Pros1	0.81	102	0.034		red	0.36	NM_031086	10930660	protein S (alpha)
Tnfrsf26	0.79	11	0.028		red	1.98	NM_001108511	10727109	tumor necrosis factor receptor superfamily, member 26
**Cell Cycle**									
Cdca7l	0.67	106	0.000		brw	11.84	NM_001034953	10887802	cell division cycle associated 7 like
Cdkn2c	0.70	67	0.012	+	red	7.59	NM_131902	10878705	cyclin-dependent kinase inhibitor 2C (p18, inhibits CDK4)
Ddit4l	0.66	127	0.000		brw	8.57	NM_080399	10819318	DNA-damage-inducible transcript 4-like
**Cytoskeleton & ECM**									
Ablim3	0.66	26	0.001		brw	8.13	XM_225889	10804750	actin binding LIM protein family, member 3
Cdh5	0.73	34	0.041		brw	12.53	NM_001107407	10809048	cadherin 5
Cltb	1.20	117	0.044		red	0.65	NM_053835	10794159	clathrin, light chain (Lcb)
Col11a1	0.58	113	0.001	+	brw	20.57	AJ005396	10818502	collagen, type XI, alpha 1
Col12a1	0.83	43	0.005		gr	0.00	U57362	10918869	collagen, type XII, alpha 1
Dnm1	0.83	14	0.028		ylw	13.35	NM_080689	10844302	dynamin 1
Dtna	1.27	99	0.023		mgt	2.14	XM_001054487	10800471	dystrobrevin alpha
Esam	0.73	45	0.001		blu	3.01	NM_001004245	10909072	endothelial cell adhesion molecule
Lama2	0.66	200	0.047		brw	14.40	XM_219866	10702096	laminin, alpha 2
Lama4	0.79	96	0.010		brw	4.59	NM_001107635	10830328	laminin, alpha 4
LOC690745	0.75	14	0.034		blu	1.19	BC088229	10770503	MOCO sulphurase C-terminal domain containing-like
Omd	0.67	164	0.007		brw	17.04	NM_031817	10797657	osteomodulin
Pcdh1	0.82	12	0.041		brw	8.75	DQ863133	10804117	protocadherin 1
Pcdh19	0.83	31	0.009		gr	0.00	XM_228429	10939226	protocadherin 19
RGD1562831	0.77	82	0.007		red	1.83	EF100686	10865956	similar to osteoclast inhibitory lectin
RGD1563615	0.69	46	0.006		trq	5.65	XM_001064593	10793431	similar to Contactin associated protein-like 3 precursor (Cell recognition molecule Caspr3)
Rpesp	1.29	21	0.020		blu	6.26	XM_216317	10867102	RPE-spondin
Sostdc1	0.73	19	0.044		brw	11.08	NM_153737	10884274	sclerostin domain containing 1
Tspan12	0.76	37	0.006		trq	2.89	NM_001015026	10861213	tetraspanin 12
Tubal3	0.66	34	0.027		brw	5.43	NM_001106118	10799370	tubulin, alpha-like 3
Vtn	0.77	18	0.044		brw	7.28	NM_019156	10736273	vitronectin
**Development**									
Aard	1.24	185	0.019		brw	2.88	NM_145093	10896503	alanine and arginine rich domain containing protein
Cpne7	0.70	56	0.005		red	0.98	NM_001108454	10808603	copine VII
Cpne8	0.75	52	0.006		grlw	1.66	NM_001108750	10906373	copine VIII
Leprel1	0.66	85	0.014		brw	7.37	NM_001025627	10751903	leprecan-like 1
Nov	0.53	168	0.001		brw	12.52	NM_030868	10896541	nephroblastoma overexpressed gene
Pkd2l2	0.67	46	0.010		red	4.07	NM_001106156	10800865	polycystic kidney disease 2-like 2
Sema7a	0.79	46	0.007		ylw	6.13	NM_001108153	10910406	sema domain, immunoglobulin domain (Ig), and GPI membrane anchor,

									(semaphorin) 7A
LOC689757	0.66	42	0.027		brw	7.84	NM_001101019	10865960	similar to osteoclast inhibitory lectin
Slit2	0.80	33	0.005		ylw	4.98	AF141386	10777137	slit homolog 2 (Drosophila)
Sox18	0.70	17	0.001		blu	2.20	NM_001024781	10852595	SRY (sex determining region Y)-box 18
Sv2a	1.21	14	0.038		ylw	6.94	NM_057210	10817512	synaptic vesicle glycoprotein 2a
Tor3a	0.79	62	0.041		red	4.44	NM_001009683	10768998	torsin family 3, member A
**Electron Transport**									
Cyp20a1	0.77	44	0.000		brw	4.62	NM_199401	10923768	cytochrome P450, family 20, subfamily A, polypeptide 1
LOC679752	0.75	24	0.035		gr	0.00	XP_001054541	10782424	similar to Glutaredoxin-1 (Thioltransferase-1) (TTase-1)
**Epigenetics**									
Cphx	0.70	142	0.030		brw	8.26	XM_577474	10786193	cytoplasmic polyadenylated homeobox
Dnmt1	0.66	561	0.006	+	brw	22.95	NM_053354	10915437	DNA (cytosine-5-)-methyltransferase 1
Ftsjd1	0.81	61	0.034		red	4.63	NM_001106186	10807841	FtsJ methyltransferase domain containing 1
H1foo	0.43	118	0.001		red	8.35	NM_001109351	10858077	H1 histone family, member O, oocyte-specific
Kdm4c	0.82	106	0.000		brw	10.58	BC158850	10869590	lysine (K)-specific demethylase 4C
**Growth Factors, Cyto-, and Chemokines**									
Angpt1	0.55	21	0.003		brw	7.75	NM_053546	10903529	angiopoietin 1
Cxcr4	0.72	120	0.049		blu	0.98	NM_022205	10767373	chemokine (C-X-C motif) receptor 4
Cxcr7	0.83	100	0.038		red	1.35	NM_053352	10925291	chemokine (C-X-C motif) receptor 7
Dll4	0.66	68	0.003		brw	12.31	NM_001107760	10838813	delta-like 4 (Drosophila)
Fap	0.71	46	0.000		red	2.64	NM_138850	10845681	fibroblast activation protein, alpha
Fgf16	0.74	19	0.024		blu	0.76	NM_021867	10934569	fibroblast growth factor 16
Hbegf	0.79	20	0.044		gr	0.00	NM_012945	10803947	heparin-binding EGF-like growth factor
Il15	0.72	31	0.001		brw	12.18	NM_013129	10806899	interleukin 15
Wfdc1	1.25	46	0.023		red	0.43	NM_133581	10808348	WAP four-disulfide core domain 1
**Immune Response**									
Cd109	0.80	11	0.003		red	0.52	NM_001108771	10911818	CD109 molecule
LOC287167	0.36	1108	0.002	+	brw	21.62	NM_001013853	10741765	globin, alpha
Icoslg	0.69	35	0.004		brw	11.67	XM_574731	10832232	inducible T-cell co-stimulator ligand
Ica1	0.80	20	0.034		brw	5.50	NM_030844	10861005	islet cell autoantigen 1
Ly6g6e	0.72	105	0.007		trq	10.07	NM_001001972	10831229	lymphocyte antigen 6 complex, locus G6E
Mill2	0.77	13	0.004		brw	2.76	NM_001017468	10704659	Mill2 gene for MHC class I-like located near the LRC, 2, exon 3, partial cds, strain:LEW.1lm1
**Metabolism & Transport**									
Accs	0.74	32	0.009		brw	5.23	BC083866	10847600	1-aminocyclopropane-1-carboxylate synthase homolog (Arabidopsis)(non-functional)
Hmgcs2	0.59	495	0.000	+	brw	19.48	NM_173094	10817759	3-hydroxy-3-methylglutaryl-Coenzyme A synthase 2 (mitochondrial)
Abat	0.73	20	0.001		blu	1.50	NM_031003	10740529	4-aminobutyrate aminotransferase
Ampd3	0.68	73	0.023		blu	1.72	NM_031544	10709880	adenosine monophosphate deaminase 3
Aox3	0.66	16	0.000		brw	14.59	NM_001008527	10923432	aldehyde oxidase 3
Asns	0.76	15	0.002		ylw	1.95	NM_013079	10860951	asparagine synthetase
Bcat1	0.65	73	0.000		tan	2.90	NM_017253	10866819	branched chain aminotransferase 1, cytosolic

Cacna2d3	0.72	209	0.003	+	brw	19.93	NM_175595	10789819	calcium channel, voltage-dependent, alpha2/delta subunit 3
Clcc1	0.77	345	0.009		red	3.61	NM_133414	10818395	chloride channel CLIC-like 1
Ero1l	0.78	92	0.027		prp	6.02	NM_138528	10782826	ERO1-like (S. cerevisiae)
Accs	0.74	32	0.009		brw	5.23	BC083866	10847600	1-aminocyclopropane-1-carboxylate synthase homolog (Arabidopsis)(non-functional)
Hmgcs2	0.59	495	0.000	+	brw	19.48	NM_173094	10817759	3-hydroxy-3-methylglutaryl-Coenzyme A synthase 2 (mitochondrial)
Galt	0.80	28	0.002		red	0.39	NM_001013089	10868384	galactose-1-phosphate uridylyltransferase
Hp	0.80	28	0.029		blu	2.64	NM_012582	10810923	haptoglobin
Hba-a2	0.69	1202	0.025		brw	16.47	NM_013096	10741756	hemoglobin alpha, adult chain 2
Hba-a2	0.68	1110	0.033		brw	15.99	NM_013096	10741778	hemoglobin alpha, adult chain 2
Hbq1	0.11	487	0.000	+	brw	23.99	XM_001061675	10741761	hemoglobin, theta 1
Hbq1	0.36	158	0.000	+	brw	22.43	XM_347266	10741774	hemoglobin, theta 1
Hbq1	0.41	106	0.001	+	brw	21.32	XM_347266	10741770	hemoglobin, theta 1
Mthfd1l	0.83	25	0.001		prp	2.91	NM_001108462	10702626	methylenetetrahydrofolate dehydrogenase (NADP+ dependent) 1-like
Npr2	0.79	94	0.032		blu	5.53	NM_053838	10868552	natriuretic peptide receptor B/guanylate cyclase B (atrionatriuretic peptide receptor B)
Neil2	0.78	17	0.016		blu	1.00	NM_001107270	10784464	nei like 2 (E. coli)
Ppap2b	0.79	71	0.037		red	2.36	NM_138905	10870481	phosphatidic acid phosphatase type 2B
Pygl	0.69	54	0.043		prp	4.55	NM_022268	10890441	phosphorylase, glycogen, liver
Kcnj8	0.73	36	0.019		red	2.63	NM_017099	10866718	potassium inwardly-rectifying channel, subfamily J, member 8
Kcnma1	0.76	12	0.000		brw	14.19	NM_031828	10778953	potassium large conductance calcium-activated channel, subfamily M, alpha member 1
Plod2	0.62	616	0.000	+	brw	18.31	NM_175869	10912255	procollagen lysine, 2-oxoglutarate 5-dioxygenase 2
P4ha1	0.77	178	0.039		prp	8.59	NM_172062	10931308	procollagen-proline, 2-oxoglutarate 4-dioxygenase (proline 4-hydroxylase), alpha polypeptide I
Ptges	0.67	56	0.002		red	2.88	NM_021583	10844223	prostaglandin E synthase
Sfxn5	1.21	20	0.017		brw	4.59	NM_153298	10863639	sideroflexin 5
LOC365687	0.80	28	0.011		blc	0.51	XR_007344	10813244	similar to glyceraldehyde-3-phosphate dehydrogenase
Scn3a	0.66	18	0.012	+	brw	17.43	NM_013119	10845809	sodium channel, voltage-gated, type III, alpha
Slc16a3	0.70	17	0.006		prp	7.79	NM_030834	10740209	solute carrier family 16, member 3 (monocarboxylic acid transporter 4)
Slc24a4	0.74	18	0.023		brw	15.20	NM_001108051	10886429	solute carrier family 24 (sodium/potassium/calcium exchanger), member 4
Slc24a6	0.79	31	0.034		red	0.41	NM_001017488	10762304	solute carrier family 24 (sodium/potassium/calcium exchanger), member 6
Slc25a30	0.81	74	0.016		trq	4.42	NM_001013187	10785326	solute carrier family 25, member 30
Slc44a3	0.66	36	0.009		brw	8.98	NM_001013914	10826451	solute carrier family 44, member 3
Slc44a4	0.65	47	0.000		brw	12.37	NM_212541	10831308	solute carrier family 44, member 4
Slc9a7	0.79	39	0.002		brw	14.01	NM_001108242	10932139	solute carrier family 9 (sodium/hydrogen

									exchanger), member 7
Vamp5	0.78	53	0.002		brw	11.91	NM_053555	10863215	vesicle-associated membrane protein 5
**Proteolysis**									
LOC494529	0.83	22	0.007		red	0.31	NM_001009974	10906037	92Aa-Protein
Adamts1	0.77	64	0.011		brw	2.47	NM_024400	10752839	ADAM metallopeptidase with thrombospondin type 1 motif, 1
Adamts6	0.74	38	0.049		brw	4.22	NM_001108544	10812834	ADAM metallopeptidase with thrombospondin type 1 motif, 6
Capn6	1.24	107	0.029		ylw	6.77	NM_031808	10932759	calpain 6
Cpa3	1.76	82	0.000		gr	0.00	NM_019300	10822493	carboxypeptidase A3, mast cell
Cma1	1.91	26	0.011		gr	0.00	NM_013092	10783989	chymase 1, mast cell
Masp1	0.64	65	0.036		brw	8.01	NM_022257	10751948	mannan-binding lectin serine peptidase 1
Mcpt10	1.59	15	0.000		gr	0.00	NM_017146	10780585	mast cell protease 10
Mcpt10	1.40	13	0.005		gr	0.00	NM_017146	10784004	mast cell protease 10
Mcpt2	1.43	41	0.025		gr	0.00	NM_172044	10780561	mast cell protease 2
Mmp7	0.59	23	0.044		tan	6.39	NM_012864	10907945	matrix metallopeptidase 7
Map1d	0.82	13	0.014		blu	0.27	NM_001107812	10836829	methionine aminopeptidase 1D
Rnf181	0.79	100	0.012		brw	11.18	NM_001007647	10863187	ring finger protein 181
Rnf208	1.25	17	0.000		red	0.50	NM_001109195	10834157	ring finger protein 208
RGD1565983	0.83	14	0.001		blu	1.53	BC127530	10937512	similar to apurinic/apyrimidinic endonuclease 2
**Receptors & Binding Proteins**									
Abca8	0.74	35	0.005		brw	8.23	XM_001081601	10748564	ATP-binding cassette, sub-family A (ABC1), member 8
Abca8a	0.49	148	0.008		red	3.85	XM_221100	10748601	ATP-binding cassette, sub-family A (ABC1), member 8a
Abcc9	0.73	59	0.000		red	3.85	NM_013040	10866724	ATP-binding cassette, sub-family C (CFTR/MRP), member 9
Abcc9	0.69	110	0.001		red	3.98	NM_013040	10866760	ATP-binding cassette, sub-family C (CFTR/MRP), member 9
Adora1	1.27	14	0.001		brw	6.83	NM_017155	10767771	adenosine A1 receptor
Chrne	0.80	10	0.018		red	0.57	NM_017194	10744472	cholinergic receptor, nicotinic, epsilon
Ednrb	0.60	576	0.012	+	brw	20.70	NM_017333	10785724	endothelin receptor type B
Egfr	0.80	38	0.022		brw	10.81	NM_031507	10774274	epidermal growth factor receptor
Flt1	0.82	12	0.049		blu	1.20	NM_019306	10756418	fms-related tyrosine kinase 1
Gabbr1	0.81	36	0.003		brw	8.65	NM_031028	10830857	gamma-aminobutyric acid (GABA) B receptor 1
Igfbp3	0.71	195	0.025		blu	2.40	NM_012588	10778390	insulin-like growth factor binding protein 3
Il17rb	0.66	30	0.002		brw	13.26	NM_001107290	10789857	interleukin 17 receptor B
Il6st	0.80	338	0.011		brw	4.85	NM_001008725	10813007	interleukin 6 signal transducer
Lhcgr	0.54	33	0.018		brw	14.41	NM_012978	10888343	luteinizing hormone/choriogonadotropi n receptor
S100g	0.48	95	0.041		tan	3.90	NM_012521	10933479	S100 calcium binding protein G
Vldlr	0.63	249	0.000		red	2.13	NM_013155	10714576	very low density lipoprotein receptor
**Signaling**									
Adm	0.73	15	0.026		prp	2.75	NM_012715	10709875	adrenomedullin
Centd3	0.76	16	0.035		brw	7.84	XM_001066167	10804086	centaurin, delta 3
Chn2	0.73	95	0.023		brw	14.95	NM_032084	10855615	chimerin (chimaerin) 2
Csrp2	0.77	27	0.003		brw	10.64	NM_177425	10895359	cysteine and glycine-rich protein 2
Eps8	0.81	54	0.003		brw	1.69	XM_232499	10866544	epidermal growth factor receptor pathway substrate 8

Fxyd5	0.80	18	0.015		ylw	3.97	NM_021909	10720859	FXYD domain-containing ion transport regulator 5
Fgd5	0.81	11	0.037		red	0.69	NM_001108637	10857382	FYVE, RhoGEF and PH domain containing 5
Gpr116	0.67	75	0.020		blu	1.36	NM_139110	10926698	G protein-coupled receptor 116
Gpr19	0.80	16	0.005		red	6.68	NM_080579	10866410	G protein-coupled receptor 19
Grk5	0.79	30	0.004		red	1.47	NM_030829	10716480	G protein-coupled receptor kinase 5
Gria3	0.77	78	0.039		red	1.22	NM_001112742	10936215	glutamate receptor, ionotrophic, AMPA 3
Gem	0.80	59	0.030		grlw	0.58	NM_001106637	10867657	GTP binding protein (gene overexpressed in skeletal muscle)
Oosp1	0.70	303	0.015		red	5.11	XM_001076054	10728954	oocyte secreted protein 1
Pik3cd	0.72	55	0.001		brw	11.29	NM_001108978	10881777	phosphatidylinositol 3-kinase catalytic delta polypeptide
Pde7b	0.63	119	0.031	+	brw	17.78	NM_080894	10717069	phosphodiesterase 7B
Pid1	0.80	20	0.044		brw	6.06	NM_001110493	10929473	phosphotyrosine interaction domain containing 1
Plekha5	0.78	61	0.001		brw	5.36	AF468695	10859438	pleckstrin homology domain containing, family A member 5
Plk2	0.71	254	0.016		brw	8.63	NM_031821	10812954	polo-like kinase 2 (Drosophila)
Pbx1	0.83	120	0.002		brw	15.30	NM_001134862	10769638	pre-B-cell leukemia homeobox 1
Prkcz	1.25	31	0.049		red	1.31	NM_022507	10882126	protein kinase C, zeta
Ppp1r3c	0.74	28	0.002		red	3.25	NM_001012072	10729847	protein phosphatase 1, regulatory (inhibitor) subunit 3C
Ptprb	0.76	18	0.009		brw	8.56	NM_001108095	10895527	protein tyrosine phosphatase, receptor type, B
Pdk1	0.79	98	0.003		prp	1.60	NM_053826	10836877	pyruvate dehydrogenase kinase, isozyme 1
Arhgef6	0.82	67	0.007		brw	6.56	NM_001005565	10939866	Rac/Cdc42 guanine nucleotide exchange factor (GEF) 6
Rassf9	1.22	48	0.001		trq	6.29	NM_022959	10895247	Ras association (RalGDS/AF-6) domain family (N-terminal) member 9
Rasgrp3	0.76	53	0.005		brw	8.36	NM_001108009	10888404	RAS guanyl releasing protein 3 (calcium and DAG-regulated)
Rasip1	0.79	14	0.018		brw	3.37	NM_001106261	10706909	Ras interacting protein 1
Rerg	0.73	30	0.045		blu	0.97	XM_578417	10866535	RAS-like, estrogen-regulated, growth-inhibitor
Ramp1	0.78	24	0.037		ylw	5.20	NM_031645	10925365	receptor (G protein-coupled) activity modifying protein 1
Rhobtb1	0.82	80	0.033		blu	5.21	NM_001107622	10832772	Rho-related BTB domain containing 1
RGD1562230	0.76	13	0.006		brw	14.42	BC166848	10829933	similar to catenin alpha 3
RGD1564641	0.78	26	0.018		brw	6.70	XR_008794	10754592	similar to Neurogenic locus notch homolog protein 2 precursor (Notch 2) (hN2)
Snx16	0.83	33	0.006		grn	1.27	NM_022289	10814244	sorting nexin 16
Tlr4	0.69	30	0.001		red	1.37	NM_019178	10869535	toll-like receptor 4
Tmem146	0.74	20	0.046		blu	8.19	NM_001134984	10931398	transmembrane protein 146
**Transcription**									
Erg	0.82	17	0.037		brw	15.52	NM_133397	10753279	avian erythroblastosis virus E-26 (v-ets) oncogene related
Bcl6b	0.75	18	0.005		brw	9.92	NM_001108279	10744376	B-cell CLL/lymphoma 6, member B (zinc finger protein)
Btg4	0.71	11	0.049	+	red	7.36	NM_001013176	10909937	B-cell translocation gene 4
Cdca7l	0.76	18	0.002		brw	5.80	NM_001034953	10840650	cell division cycle

									associated 7 like
Ccdc109b	0.77	60	0.046		brw	8.97	XM_215707	10826779	coiled-coil domain containing 109B
Ccdc3	0.78	11	0.007		grn	2.22	XM_574081	10799548	coiled-coil domain containing 3
Ccdc69	0.74	39	0.047		brw	5.36	NM_001109031	10742744	coiled-coil domain containing 69
Cish	1.26	22	0.030		trq	14.80	NM_031804	10912908	cytokine inducible SH2-containing protein
Duxbl	0.64	88	0.012		brw	12.60	XM_224643	10786197	double homeobox B-like
Ezh2	0.82	77	0.040		brw	10.01	NM_001134979	10862338	enhancer of zeste homolog 2 (Drosophila)
Fbxo15	0.68	75	0.015	+	red	11.92	NM_001108436	10803025	F-box protein 15
Fbxo36	0.72	37	0.023		red	1.29	NM_001108804	10924798	F-box protein 36
Gtf2a1l	0.80	13	0.027		brw	3.96	NM_001012136	10882864	general transcription factor IIA, 1-like
Klf17	0.64	47	0.006		red	5.61	XM_233437	10879025	Kruppel-like factor 17
Mterf	0.81	47	0.015		red	3.79	NM_053499	10853477	mitochondrial transcription termination factor
Mlf1ip	0.82	17	0.036		red	2.40	NM_001025673	10791661	myeloid leukemia factor 1 interacting protein
Pdzrn3	0.75	47	0.001		brw	15.80	XM_232226	10864459	PDZ domain containing RING finger 3
RGD1304870	0.44	578	0.015		red	9.77	XM_001081215	10737399	similar to Tceb2 protein
RGD1561118	0.43	172	0.007		red	8.17	XM_220759	10746154	similar to transcription elongation factor B (SIII), polypeptide 2
LOC691037	0.81	11	0.005		brw	2.99	XM_001068672	10856263	similar to zinc finger, matrin type 1 isoform 3
Stx3	0.77	29	0.043		pink	0.58	NM_031124	10728960	syntaxin 3
Txnip	0.75	877	0.025		prp	2.45	NM_001008767	10817552	thioredoxin interacting protein
Tcf4	0.79	147	0.034		brw	12.34	NM_053369	10802520	transcription factor 4
Tcfap2c	0.74	12	0.005		grn	4.26	NM_201420	10842551	transcription factor AP-2, gamma
Tp53i11	0.81	30	0.025		brw	4.52	NM_001107749	10838056	tumor protein p53 inducible protein 11
Ets2	0.81	30	0.017		brw	2.96	NM_001107107	10750460	v-ets erythroblastosis virus E26 oncogene homolog 2 (avian)
Zeb2	0.62	104	0.002		brw	12.01	NM_001033701	10845072	zinc finger E-box binding homeobox 2
Zfp423	1.21	26	0.024		gr	0.00	NM_053583	10806341	zinc finger protein 423
Zfyve19	0.83	27	0.012		blu	4.83	NM_001034948	10838779	zinc finger, FYVE domain containing 19
**Translation & Protein Modification**									
Oas1d	0.66	70	0.050		brw	6.27	NM_001009379	10762226	2'-5' oligoadenylate synthetase 1D
Oas1e	0.65	74	0.006		red	5.44	NM_001009492	10758785	2'-5' oligoadenylate synthetase 1E
Cpsf4l	0.75	13	0.006		red	4.41	XM_221075	10748782	cleavage and polyadenylation specific factor 4-like
Eif2b3	0.82	171	0.037		red	5.14	NM_133609	10871369	eukaryotic translation initiation factor 2B, subunit 3 gamma
Larp6	0.71	24	0.000		brw	3.53	NM_001108154	10910612	La ribonucleoprotein domain family, member 6
Npm2	0.69	231	0.016		brw	10.18	NM_203340	10785135	nucleophosmin/nucleoplasmin 2
Rnase1l1	0.77	11	0.002		blu	1.04	NM_001013232	10783188	ribonuclease, RNase A family, 1-like 1 (pancreatic)
Slfn3	0.74	11	0.046		gr	0.00	NM_053687	10736802	schlafen 3
**Miscellaneous & Unknown**									
Cyyr1	0.74	25	0.008		brw	8.57	NM_001013980	10752831	cysteine/tyrosine-rich 1
Depdc2	0.49	225	0.000	+	brw	29.46	NM_001107899	10875023	DEP domain containing 2
Fam154a	0.65	27	0.037	+	red	11.43	XM_001054528	10877890	family with sequence similarity 154, member A
Fam38b	0.49	95	0.000		red	2.52	XM_001066259	10804821	family with sequence similarity 38, member B

LOC680214	0.53	128	0.004		red	7.23	XM_001056138	10937233	hypothetical protein LOC680214
LOC685621	0.73	14	0.006		red	9.72	XM_001064548	10857151	hypothetical protein LOC685621
Plbd1	0.75	26	0.031		brw	4.27	NM_001013927	10866459	phospholipase B domain containing 1
RGD1308023	0.78	15	0.000	+	brw	4.83	XR_006437	10850484	similar to CG5521-PA
RGD1308023	0.79	50	0.032	+	brw	17.64	XR_006437	10850490	similar to CG5521-PA
LOC682889	0.66	82	0.000		red	2.92	XM_001063568	10804834	similar to CG8486-PA, isoform A
RGD1565680	0.74	351	0.021		red	9.66	XM_578505	10873805	similar to Hypothetical protein DJ1198H6.2
LOC500046	0.76	221	0.007		brw	16.36	XM_001061323	10853931	similar to hypothetical protein FLJ21986
RGD1559690	0.78	87	0.038		red	7.16	NM_001126294	10723689	similar to hypothetical protein FLJ25416
RGD1306866	0.62	63	0.000		red	2.51	XM_225880	10804843	similar to Hypothetical protein KIAA0233
RGD1308517	0.82	80	0.017		brw	9.29	BC107470	10788016	similar to KIAA1712 protein
RGD1566021	0.59	138	0.030	+	brw	19.63	XM_001071780	10800122	similar to KIAA1772
RGD1563946	0.81	19	0.034		brw	3.31	XR_008586	10790780	similar to mKIAA1623 protein
Ttc6	0.71	11	0.042		blu	3.96	XM_216713	10884719	tetratricopeptide repeat domain 6
Tmcc2	0.82	15	0.026		grn	3.19	XM_223107	10767597	transmembrane and coiled-coil domain family 2
Trim13	0.79	48	0.046		red	5.65	NM_001012210	10780924	tripartite motif-containing 13
Wdr54	0.80	13	0.046		prp	5.78	NM_001109245	10863502	WD repeat domain 54
Fam171b	0.82	49	0.009		blu	1.09	XM_575160	10837310	family with sequence similarity 171, member B
**ESTs**									
MGC72974	0.79	63	0.002		red	1.03	NM_198772	10859766	hypothetical LOC316976
RGD1305537	0.83	20	0.006		red	0.43	NM_001108822	10740359	similar to RIKEN cDNA 3110001I22
RGD1306186	0.71	133	0.003	+	red	9.20	BC090317	10881318	similar to RIKEN cDNA 4930569K13
RGD1311251	0.80	50	0.014		grn	2.51	BC092578	10910004	similar to RIKEN cDNA 4930550C14
RGD1311589	0.56	28	0.002		brw	16.95	BC127529	10855632	similar to RIKEN cDNA E130201N16
RGD1559731	0.56	45	0.044		brw	12.03	XM_576913	10932166	similar to RIKEN cDNA 4930578C19
RGD1560559	0.69	30	0.050		gr	0.00	XM_221310	10755281	similar to RIKEN cDNA 2310042E22
RGD1562059	1.21	272	0.027		mgt	0.30	NM_001108558	10817215	similar to RIKEN cDNA 1110038F21
RGD1563349	0.67	30	0.001		brw	17.30	NM_001109345	10860566	similar to RIKEN cDNA 9330182L06
RGD1563349	0.67	32	0.000		brw	14.69	NM_001109345	10860531	similar to RIKEN cDNA 9330182L06
RGD1564091	0.60	86	0.001		brw	15.64	XM_001074126	10889291	similar to Kiaa0575
RGD1565616	0.83	64	0.001		brw	7.67	NM_001109206	10849967	RGD1565616
	1.23	28	0.025		trq	5.73	31342474	10805603	
	0.79	65	0.003		ylw	8.79	59896131, 41350416	10769693	
	0.79	89	0.000		brw	1.47	BC099122	10787782	Rattus norvegicus cDNA clone IMAGE:7462850.
	0.80	81	0.017		red	2.77	DQ486884	10771535	
	0.82	29	0.002		brw	1.17	ENSRNOT00000003115	10731326	
	1.26	15	0.027		gr	0.00	ENSRNOT00000006485	10883085	
	0.62	162	0.014		blu	6.79	ENSRNOT00000013418	10839050	
	0.80	15	0.000		brw	2.81	ENSRNOT00000041624	10884408	
	0.49	65	0.012		brw	12.07	ENSRNOT00000043281	10860179	
	0.76	16	0.021		red	2.45	ENSRNOT00000045813	10842669	
	0.82	43	0.012		grn	4.13	ENSRNOT0000	10759427	

							0048419		
	0.82	17	0.034		brw	1.55	ENSRNOT0000 0051655	10937641	
	1.25	11	0.000		blu	1.31	ENSRNOT0000 0052541	10911811	
	0.81	18	0.030		red	0.40	ENSRNOT0000 0052697	10878963	
	0.76	37	0.005		brw	1.67	ENSRNOT0000 0053532	10891371	
	0.80	14	0.034		blc	0.76	ENSRNOT0000 0053924	10716742	
	0.71	33	0.038		brw	3.93	ENSRNOT0000 0054304	10716148	
	0.79	92	0.020		blu	3.21	ENSRNOT0000 0055219	10851303	
	0.82	39	0.013		blu	2.04	ENSRNOT0000 0056248	10926080	
	0.74	37	0.001		gr	0.00	ENSRNOT0000 0057420	10833416	
	0.74	67	0.018		brw	12.51	ENSRNOT0000 0058046	10863213	
	0.66	78	0.001		brw	11.06	ENSRNOT0000 0058915	10802541	
	0.74	37	0.001		gr	0.00	ENSRNOT0000 0059477	10791602	
	0.67	122	0.001		red	2.31	ENSRNOT0000 0059678	10845070	
	0.79	15	0.011		ylw	1.37	ENSRNOT0000 0059904	10801687	
	0.82	19	0.005		brw	3.15	ENSRNOT0000 0060468	10801258	
	0.81	12	0.016		brw	2.13	ENSRNOT0000 0060470	10801256	
	0.77	11	0.013		pink	0.40	ENSRNOT0000 0060844	10861001	
	0.83	83	0.000		brw	4.20	ENSRNOT0000 0061943	10875093	
	0.71	17	0.000		brw	2.85	rno-mir-128-1	10763685	MI0000900 Rattus norvegicus miR-128-1 stem-loop
	0.64	23	0.017		red	0.74	rno-mir-218-1	10777176	MI0000958 Rattus norvegicus miR-218-1 stem-loop
	0.81	13	0.043		red	0.59	rno-mir-374	10938826	MI0003552 Rattus norvegicus miR-374 stem-loop

G. Genes influenced by treatment with leukemia inhibitory factor (LIF) - 349 probe sets									
**Apoptosis**									
**Gene Symbol**	**GF/Con Ratio**	**mean_ diff***	**t-test p-value**	**Short 55 list**	**Module ****	**k.in *****	**GenBank/ Transcript ID**	**Probe Set ID**	**Gene Title/Description**
Bax	1.26	17	0.006		blc	0.27	AF235993	10721834	Bcl2-associated X protein
Casp1	1.32	40	0.023		blu	7.55	NM_012762	10907815	caspase 1
Clu	2.50	960	0.021		grlw	4.98	NM_053021	10781166	clusterin
L1td1	0.79	21	0.021		grn	4.77	XM_001059852	10870092	LINE-1 type transposase domain containing 1
Lsp1	1.42	115	0.014		blc	0.96	NM_001025420	10712477	lymphocyte-specific protein 1
LOC683447	1.39	31	0.000		ylw	6.80	XM_001066154	10727116	similar to Tumor necrosis factor receptor superfamily member 23 precursor (Tumor necrosis factor receptor p60 homolog 1) (TNF receptor family member SOB) (Decoy TRAIL receptor 1) (TNF receptor homolog 1)
Tnip2	1.24	14	0.003		blc	0.40	NM_001024771	10773496	TNFAIP3 interacting protein 2
Tradd	1.21	12	0.007		pink	1.55	AF517017	10810556	TNFRSF1A-associated via death domain
Tnfrsf1a	1.65	182	0.027		ylw	9.03	NM_013091	10858967	tumor necrosis factor receptor superfamily, member 1a
**Cell Cycle**									
Ccno	0.81	577	0.001		red	2.65	NM_001109175	10813087	cyclin O
Nubp2	1.22	82	0.016		slm	0.76	NM_001011891	10741248	nucleotide binding protein 2
**Cytoskeleton & ECM**									
Cdh17	1.42	14	0.011		gr	0.00	NM_053977	10867667	cadherin 17
Cthrc1	1.31	40	0.034		ylw	1.36	NM_172333	10896263	collagen triple helix repeat containing 1
Col20a1	0.83	25	0.007		brw	5.78	XM_230973	10842962	collagen, type XX, alpha 1
Emp2	0.75	111	0.048		brw	3.58	NM_001007721	10731531	epithelial membrane protein 2
Epb4.1l3	1.25	36	0.003		trq	1.43	NM_053927	10925936	erythrocyte protein band 4.1-like 3
Epb4.9	0.76	15	0.026		gr	0.00	NM_001108385	10785116	erythrocyte protein band 4.9
Fgg	2.64	103	0.041		grlw	4.02	NM_012559	10816067	fibrinogen gamma chain
Fgl2	1.29	63	0.000		prp	0.56	NM_053455	10860231	fibrinogen-like 2
Gpm6a	0.76	98	0.010		gr	0.00	NM_178105	10791552	glycoprotein m6a
Kif1a	1.36	13	0.000		grlw	2.89	AM180765	10929937	kinesin family member 1A
Lad1	0.82	31	0.043		grn	4.91	NM_001107942	10869693	ladinin 1
Lgmn	1.32	312	0.019		grn	1.01	NM_022226	10891834	legumain
Lrfn1	0.83	14	0.001		gr	0.00	NM_001127694	10705519	leucine rich repeat and fibronectin type III domain containing 1
Map1lc3b	1.37	59	0.000		mbl	2.27	NM_022867	10792602	microtubule-associated protein 1 light chain 3 beta
Myo6	0.82	62	0.013		blu	2.59	XM_236444	10911882	myosin VI
Myo10	0.80	112	0.009		blu	3.73	NM_001107657	10813894	myosin X
Prg4	1.91	32	0.008		grlw	2.19	NM_001105962	10768412	proteoglycan 4, (megakaryocyte stimulating factor, articular superficial zone protein, camptodactyly, arthropathy, coxa vara, pericarditis syndrome)
Pcdh1	0.74	17	0.009		brw	8.75	DQ863133	10804117	protocadherin 1
RGD1566146	0.76	54	0.044		blc	8.40	XM_577126	10747185	similar to keratin associated protein 4-10
LOC681362	0.78	180	0.011		trq	12.71	XM_001061409	10796679	similar to p130Cas-associated protein (p140Cap) (SNAP-25-interacting protein) (SNIP)

Sync	1.28	34	0.018		gr	0.00	NM_001108683	10872307	syncoilin
Sdc4	1.24	335	0.047		ylw	4.58	NM_012649	10851599	syndecan 4
Tspan33	0.83	21	0.025		gr	0.00	NM_001109227	10854199	tetraspanin 33
Tnnc1	1.43	145	0.042		red	2.28	NM_001034105	10786640	troponin C type 1 (slow)
**Development**									
Auts2l	0.77	84	0.013		pink	1.84	NM_001107136	10761297	autism susceptibility candidate 2-like
Cpne7	0.75	46	0.001		red	0.98	NM_001108454	10808603	copine VII
Cpne8	1.63	179	0.040		grlw	1.78	NM_001108750	10837621	copine VIII
Crb3	0.78	11	0.012		grn	4.07	NM_001025661	10931336	crumbs homolog 3 (Drosophila)
Egln3	0.79	46	0.048		prp	1.24	NM_019371	10889923	EGL nine homolog 3 (C. elegans)
Fhdc1	1.31	22	0.028		ylw	3.79	NM_001106437	10824031	FH2 domain containing 1
Gcom1	0.78	46	0.047		blu	1.75	NM_001014211	10918586	GRINL1A complex locus
Mgp	1.44	614	0.002		ylw	10.11	NM_012862	10866512	matrix Gla protein
Ntn2l	0.80	15	0.012		grn	0.46	AY028418	10741009	netrin 2-like (chicken)
Nptx2	1.28	41	0.023		mgt	2.72	NM_001034199	10760290	neuronal pentraxin 2
Nnat	0.75	47	0.013		gr	0.00	NM_053601	10841637	neuronatin
Odz4	0.70	121	0.001		blu	5.03	XM_218927	10708695	odz, odd Oz/ten-m homolog 4 (Drosophila)
Olfml3	1.42	646	0.016		grn	3.99	NM_001107708	10825575	olfactomedin-like 3
Prrx2	1.26	11	0.022		grn	3.78	NM_001105739	10835257	paired related homeobox 2
Pcp4l1	1.50	58	0.036		mgt	2.00	NM_001126093	10769812	Purkinje cell protein 4-like 1
Reln	0.83	16	0.023		red	0.20	NM_080394	10853020	reelin
Sectm1b	1.82	53	0.000		grn	2.69	NM_199082	10749762	secreted and transmembrane 1B
LOC689800	1.32	31	0.001		ylw	6.93	EU128749	10859090	similar to osteoclast inhibitory lectin
Vwa5a	1.33	322	0.027		grlw	1.46	NM_198755	10909210	von Willebrand factor A domain containing 5A
**Electron Transport**									
Cyp1b1	1.41	570	0.026		trq	6.18	NM_012940	10887947	cytochrome P450, family 1, subfamily b, polypeptide 1
**Epigenetics**									
Brdt	0.83	42	0.025		grn	7.08	NM_001012031	10775204	bromodomain, testis-specific
Gadd45a	1.37	231	0.036		grlw	0.22	NM_024127	10862867	growth arrest and DNA-damage-inducible, alpha
H1foo	0.63	77	0.037		red	8.35	NM_001109351	10858077	H1 histone family, member O, oocyte-specific
H19	1.36	833	0.004		mgt	2.23	NR_027324	10726991	H19 fetal liver mRNA
H19	1.50	597	0.013		mgt	2.24	NR_027324	10726995	H19 fetal liver mRNA
**Golgi Apparatus**									
Cog7	1.22	23	0.046		mgt	0.39	NM_001033889	10725427	component of oligomeric golgi complex 7
Gal3st4	1.21	27	0.001		grn	3.60	NM_001109064	10760891	galactose-3-O-sulfotransferase 4
Glb1l3	1.49	167	0.002		mgt	0.99	NM_001024358	10915864	galactosidase, beta 1-like 3
**Growth Factors, Cyto-, and Chemokines**									
C1qtnf6	1.97	275	0.020		grn	5.20	NM_001034932	10905307	C1q and tumor necrosis factor related protein 6
Ctgf	0.69	73	0.006	+	blu	8.18	NM_022266	10717233	connective tissue growth factor
Jag1	0.83	155	0.018		grn	6.04	NM_019147	10850228	jagged 1
Nrg4	0.82	14	0.031		grn	0.41	XM_001076144	10917617	neuregulin 4
Npy	1.45	55	0.000		grn	1.77	NM_012614	10855506	neuropeptide Y
Pdap1	1.23	65	0.023		blu	0.54	NM_022595	10756560	PDGFA associated protein 1
Pgf	1.28	69	0.011		mgt	1.15	NM_053595	10891240	placental growth factor
**Immune Response**									
Cd200	1.34	293	0.000		blu	1.94	NM_031518	10751091	Cd200 molecule
C1qb	1.24	32	0.000		brw	2.11	NM_019262	10880727	complement component 1, q subcomponent, beta polypeptide
C1s	1.48	477	0.001		ylw	7.70	NM_138900	10865442	complement component 1, s subcomponent
C7	2.41	745	0.038		grlw	0.46	AF309948	10821666	complement component 7
Icoslg	0.70	34	0.049		brw	11.67	XM_574731	10832232	inducible T-cell co-stimulator ligand
Ifi47	1.48	185	0.022		grn	5.48	NM_172019	10733056	interferon gamma inducible

									protein 47
Ifitm1	1.26	293	0.010		ylw	7.00	NM_001106314	10712171	interferon induced transmembrane protein 1
Ifitm3	1.28	819	0.000		grn	7.40	NM_001136124	10726682	interferon induced transmembrane protein 3
Prtg	0.81	15	0.017		grn	2.00	NM_001037651	10911484	protogenin homolog (Gallus gallus)
Reg3g	31.39	1335	0.037		grlw	4.27	NM_173097	10863410	regenerating islet-derived 3 gamma
LOC300024	0.70	26	0.024		trq	1.99	XM_216959	10904587	similar to Ly6-B antigen gene
**Metabolism & Transport**									
Acsl4	1.27	286	0.009		trq	6.41	NM_053623	10932795	acyl-CoA synthetase long-chain family member 4
Aldh9a1	1.21	12	0.002		grn	0.23	BC074019	10765411	aldehyde dehydrogenase 9 family, member A1
A4galt	0.71	41	0.011		grn	4.57	NM_022240	10905819	alpha 1,4-galactosyltransferase
Arsi	0.81	11	0.008		gr	0.00	NM_001047881	10802023	arylsulfatase family, member I
Cacna1d	0.74	38	0.001		cyan	0.59	NM_017298	10789869	calcium channel, voltage-dependent, L type, alpha 1D subunit
Cacna1h	0.76	87	0.025		grn	0.90	NM_153814	10741422	calcium channel, voltage-dependent, T type, alpha 1H subunit
Chst9	1.53	19	0.025		grlw	0.87	XM_226142	10803315	carbohydrate (N-acetylgalactosamine 4-0) sulfotransferase 9
Cp	1.27	128	0.038		gr	0.00	NM_012532	10814430	ceruloplasmin
Clic2	1.33	31	0.008		ylw	10.76	NM_001009651	10827592	chloride intracellular channel 2
Ero1l	0.82	76	0.011		prp	6.02	NM_138528	10782826	ERO1-like (S. cerevisiae)
Faah	0.81	26	0.011		trq	5.61	NM_024132	10871182	fatty acid amide hydrolase
Frrs1	1.51	190	0.001		grn	8.37	XM_227622	10818633	ferric-chelate reductase 1
Fuca1	1.25	175	0.006		tan	0.40	NM_012562	10872940	fucosidase, alpha-L- 1, tissue
Hhat	0.80	42	0.023		red	1.22	XR_008636	10770748	hedgehog acyltransferase
Hsd3b7	1.20	34	0.036		slm	1.12	NM_139329	10711210	hydroxy-delta-5-steroid dehydrogenase, 3 beta- and steroid delta-isomerase 7
Isyna1	1.45	727	0.011		grlw	1.00	NM_001013880	10790966	inositol-3-phosphate synthase 1
Ldha	0.83	574	0.001		prp	6.12	NM_017025	10908861	lactate dehydrogenase A
Ltbp1	0.77	193	0.003		blu	3.21	NM_021587	10888424	latent transforming growth factor beta binding protein 1
Lpl	1.24	27	0.001		ylw	1.44	NM_012598	10791250	lipoprotein lipase
Mpi	1.29	50	0.000		grn	1.15	NM_001004081	10917770	mannose phosphate isomerase (mapped)
Mgst1	1.29	348	0.013		ylw	7.66	NM_134349	10859392	microsomal glutathione S-transferase 1
Nos3	0.79	22	0.007		brw	12.33	NM_021838	10860076	nitric oxide synthase 3, endothelial cell
Ppapdc1a	0.81	11	0.020		red	0.51	XM_219376	10711454	phosphatidic acid phosphatase type 2 domain containing 1A
Pctp	1.21	15	0.003		mgt	0.52	NM_017225	10746201	phosphatidylcholine transfer protein
Pgm2l1	0.82	97	0.014		grn	6.52	NM_001109454	10709020	phosphoglucomutase 2-like 1
Pik3c2b	0.70	23	0.000		gr	0.00	NM_001105951	10763935	phosphoinositide-3-kinase, class 2, beta polypeptide
Plscr1	1.39	32	0.029		ylw	3.35	NM_057194	10912218	phospholipid scramblase 1
Pitpnm3	0.76	26	0.002		ylw	0.44	XM_220629	10744641	PITPNM family member 3
Kctd19	1.57	21	0.040		grlw	1.14	XM_001075666	10810614	potassium channel tetramerisation domain containing 19
Prnp	0.80	141	0.015		grn	2.47	NM_012631	10840076	prion protein
P4ha2	0.80	43	0.004		prp	6.14	NM_001108275	10733564	procollagen-proline, 2-oxoglutarate 4-dioxygenase (proline 4-hydroxylase),

									alpha polypeptide II
Prr16	1.32	19	0.000		ylw	8.61	NM_001108432	10801683	proline rich 16
RGD1561661	1.45	23	0.005		mgt	1.96	NM_001106954	10932341	similar to Ferritin light chain (Ferritin L subunit)
LOC501110	1.22	174	0.022		trq	3.30	BC079271	10926967	similar to Glutathione S-transferase A1 (GTH1) (HA subunit 1) (GST-epsilon) (GSTA1-1) (GST class-alpha)
LOC365687	0.79	30	0.049		blc	0.51	XR_007344	10813244	similar to glyceraldehyde-3-phosphate dehydrogenase
RGD1560402	0.82	12	0.040		blc	1.35	XM_574850	10812969	similar to Phosphoglycerate kinase 1
Scnn1b	1.85	55	0.035		mgt	1.74	NM_012648	10710595	sodium channel, nonvoltage-gated 1 beta
Slc16a10	0.81	64	0.016		grn	1.87	NM_138831	10833635	solute carrier family 16 (monocarboxylic acid transporters), member 10
Slc2a12	0.82	96	0.002		red	3.11	NM_001107451	10717325	solute carrier family 2 (facilitated glucose transporter), member 12
Slc26a2	1.40	87	0.001		grn	14.19	NM_057127	10804672	solute carrier family 26 (sulfate transporter), member 2
Slc39a1	1.30	398	0.039		grn	3.91	NM_001134577	10816959	solute carrier family 39 (zinc transporter), member 1
Slc5a8	0.73	12	0.035		red	8.31	XR_009344	10894778	solute carrier family 5 (iodide transporter), member 8
Sod2	1.30	556	0.040		grn	3.56	NM_017051	10717935	superoxide dismutase 2, mitochondrial
Ugdh	1.27	190	0.000		ylw	1.90	NM_031325	10772705	UDP-glucose dehydrogenase
Xdh	1.65	226	0.050		grn	3.19	NM_017154	10882933	xanthine dehydrogenase
**Proteolysis**									
Adamts5	0.81	45	0.011		brw	14.23	NM_198761	10752852	ADAM metallopeptidase with thrombospondin type 1 motif, 5
Adamtsl4	0.70	21	0.005		grn	1.94	NM_001034012	10833985	ADAMTS-like 4
Ctsc	1.26	220	0.026		grn	3.72	NM_017097	10708521	cathepsin C
Itih4	1.56	10	0.028		grlw	4.21	NM_019369	10786537	inter alpha-trypsin inhibitor, heavy chain 4
Lonrf2	0.71	32	0.003		blu	7.01	XM_237078	10927588	LON peptidase N-terminal domain and ring finger 2
Rnf213	0.68	154	0.008		cyan	9.53	XM_001081768	10739988	ring finger protein 213
Rnf213	0.72	88	0.009		cyan	9.76	XM_001081768	10739958	ring finger protein 213
Rnf213	0.66	109	0.013		cyan	9.49	XM_001081768	10739992	ring finger protein 213
Rnf213	0.71	236	0.013		cyan	9.52	XM_001081768	10739982	ring finger protein 213
Rnf213	0.70	50	0.016		cyan	7.63	XM_001081768	10739990	ring finger protein 213
Rnf213	0.71	279	0.017		cyan	9.64	XM_001081768	10739977	ring finger protein 213
Rnf213	0.72	311	0.020		cyan	9.88	XM_001081768	10739984	ring finger protein 213
Rnf213	0.75	85	0.020		cyan	8.74	XM_001081768	10739996	ring finger protein 213
Rnf213	0.74	113	0.022		cyan	8.78	XM_001081768	10740000	ring finger protein 213
Rnf213	0.71	100	0.044		cyan	9.22	XM_001081768	10739994	ring finger protein 213
Serping1	1.40	497	0.017		ylw	3.21	NM_199093	10846854	serine (or cysteine) peptidase inhibitor, clade G, member 1
**Receptors & Binding Proteins**									
Chrne	0.77	12	0.002		red	0.57	NM_017194	10744472	cholinergic receptor, nicotinic, epsilon
Folr1	1.20	29	0.006		gr	0.00	NM_133527	10724116	folate receptor 1 (adult)
Fzd1	1.30	69	0.002		ylw	4.31	NM_021266	10853469	frizzled homolog 1 (Drosophila)
Gabbr1	0.82	35	0.021		brw	8.65	NM_031028	10830857	gamma-aminobutyric acid (GABA) B receptor 1
Gfra1	1.42	73	0.040		gr	0.00	NM_012959	10731075	GDNF family receptor alpha 1
Gphn	1.25	141	0.037		trq	5.17	NM_022865	10885484	gephyrin
Il1r1	2.16	565	0.002		ylw	13.96	NM_013123	10922826	interleukin 1 receptor, type I
Il10rb	1.23	62	0.000		blc	0.39	NM_001107111	10750193	interleukin 10 receptor, beta
Il13ra1	1.21	363	0.043		trq	10.19	NM_145789	10754983	interleukin 13 receptor,

									alpha 1
Osmr	2.49	310	0.015		ylw	4.63	NM_001005384	10821698	oncostatin M receptor
Osbp2	0.82	10	0.003		grn	0.64	NM_001107232	10778001	oxysterol binding protein 2
Ptch1	0.76	220	0.008		brw	15.54	NM_053566	10793528	patched homolog 1 (Drosophila)
Plxna3	0.81	14	0.002		grlw	0.25	NM_001107581	10940324	plexin A3
Ptger2	1.34	10	0.008		grlw	1.39	NM_031088	10779553	prostaglandin E receptor 2, subtype EP2
Ptger2	1.34	10	0.008		grlw	1.39	NM_031088	10779584	prostaglandin E receptor 2, subtype EP2
Ptger3	2.06	25	0.025		grlw	4.30	NM_012704	10819905	prostaglandin E receptor 3 (subtype EP3)
Synj2bp	1.22	66	0.000		mgt	1.13	NM_022599	10890967	synaptojanin 2 binding protein
Tax1bp3	1.23	180	0.000		ylw	10.84	NM_001025419	10735662	Tax1 (human T-cell leukemia virus type I) binding protein 3
**Signaling**									
Alpk1	1.48	58	0.000		grlw	2.02	XM_227715	10826672	alpha-kinase 1
Alpk1	1.40	49	0.001		ylw	1.78	XM_001076258	10826680	alpha-kinase 1
Axin2	0.83	27	0.048		trq	7.28	NM_024355	10739302	axin2
Cib1	1.27	57	0.008		blu	3.95	NM_031145	10723032	calcium and integrin binding 1 (calmyrin)
Camkk2	0.82	22	0.011		grn	0.44	NM_031338	10758517	calcium/calmodulin-dependent protein kinase kinase 2, beta
Ctnna2	1.24	12	0.010		gr	0.00	NM_001106598	10863378	catenin (cadherin associated protein), alpha 2
Dchs1	0.83	24	0.015		brw	2.76	NM_001107544	10724542	dachsous 1 (Drosophila)
Dock3	0.82	75	0.005		ylw	2.83	NM_001108184	10920002	dedicator of cyto-kinesis 3
Defb25	0.82	14	0.023		mbl	4.19	NM_001037516	10850823	defensin beta 25
Dclk2	0.80	279	0.007		grn	8.23	NM_001009691	10824091	doublecortin-like kinase 2
Efhd1	1.33	23	0.006		gr	0.00	NM_001109310	10925014	EF-hand domain family, member D1
Fastkd1	0.76	52	0.045		grn	0.90	XM_230003	10846061	FAST kinase domains 1
Gria4	0.80	32	0.031		gr	0.00	NM_001113184	10914643	glutamate receptor, ionotropic, AMPA4
Gpcr12	0.74	11	0.011		brw	14.95	NM_001037295	10756515	G-protein coupled receptor 12
Gem	1.38	115	0.001		grlw	0.58	NM_001106637	10867657	GTP binding protein (gene overexpressed in skeletal muscle)
Hspa12b	0.79	31	0.017		red	2.45	NM_001107778	10840007	heat shock protein 12B
Impa2	0.82	48	0.018		grn	3.08	NM_172224	10802407	inositol (myo)-1(or 4)-monophosphatase 2
Irak4	1.26	14	0.000		grn	0.73	NM_001106791	10898862	interleukin-1 receptor-associated kinase 4
Jak2	1.44	430	0.023		grlw	2.68	NM_031514	10714667	Janus kinase 2
Jak3	1.28	19	0.040		ylw	1.19	NM_012855	10787364	Janus kinase 3
Lcn2	1.73	473	0.041		ylw	3.42	NM_130741	10844331	lipocalin 2
Met	1.22	105	0.001		trq	2.35	NM_031517	10853819	met proto-oncogene
Pex13	0.81	180	0.036		red	4.65	NM_001107242	10778759	peroxisomal biogenesis factor 13
Plekhh2	0.79	58	0.027		blu	0.92	XM_233830	10882594	pleckstrin homology domain containing, family H (with MyTH4 domain) member 2
Ptn	1.56	200	0.024		grn	4.66	NM_017066	10861835	pleiotrophin
Prkg2	1.44	18	0.025		grlw	1.35	NM_013012	10771506	protein kinase, cGMP-dependent, type II
Ptpn9	1.29	140	0.045		ylw	5.78	NM_001013040	10910256	protein tyrosine phosphatase, non-receptor type 9
Ptprb	0.78	16	0.020		brw	8.56	NM_001108095	10895527	protein tyrosine phosphatase, receptor type, B
Ptprk	0.79	117	0.003		blu	4.75	NM_001029902	10717118	protein tyrosine phosphatase, receptor type, K, extracellular region
Rab13	1.27	60	0.009		ylw	4.55	NM_031092	10816941	RAB13, member RAS oncogene family
Rab32	1.38	80	0.009		ylw	11.93	NM_001108902	10716785	RAB32, member RAS

									oncogene family
Rasgrp1	0.83	15	0.002		gr	0.00	NM_019211	10848393	RAS guanyl releasing protein 1 (calcium and DAG-regulated)
Rasa3	0.83	52	0.002		red	0.89	NM_031574	10789401	RAS p21 protein activator 3
Rgs11	0.76	13	0.003		prp	1.44	U32438	10732565	regulator of G-protein signaling 11
Rgnef	0.80	45	0.001		blu	1.95	NM_001108542	10820713	Rho-guanine nucleotide exchange factor
Sgk1	1.24	342	0.046		trq	4.21	NM_019232	10717331	serum/glucocorticoid regulated kinase 1
Siah2	0.81	88	0.001		grn	3.82	NM_134457	10823338	seven in absentia 2
Stap1	0.73	17	0.008		grn	4.24	NM_001025115	10776239	signal transducing adaptor family member 1
Sipa1l2	0.83	33	0.010		grn	1.28	NM_001009704	10811956	signal-induced proliferation-associated 1 like 2
RGD1561963	0.83	15	0.008		brw	5.12	XM_001060744	10929321	similar to Dedicator of cytokinesis protein 10 (Protein zizimin 3)
RGD1562220	1.25	92	0.025		mgt	1.36	XM_001068657	10771492	similar to GPI-gamma 4; GPIgamma4
RGD1560603	0.81	52	0.009		grn	5.08	XM_576963	10937932	similar to mitogen-activated protein kinase kinase kinase 15
Snx7	1.21	54	0.015		blu	1.51	NM_001012083	10826403	sorting nexin 7
**Transcription**									
Ankrd41	0.83	20	0.000		grn	0.50	XR_008568	10787275	ankyrin repeat domain 41
Bcl6	1.39	55	0.017		grlw	1.18	NM_001107084	10751931	B-cell CLL/lymphoma 6
Bcl6b	0.82	13	0.020		brw	9.92	NM_001108279	10744376	B-cell CLL/lymphoma 6, member B (zinc finger protein)
Baz2a	0.83	91	0.039		grn	4.63	NM_001107158	10892980	bromodomain adjacent to zinc finger domain, 2A
Ccdc72	1.23	65	0.023		gr	0.00	NM_001126048	10920371	coiled-coil domain containing 72
Dnajc15	1.23	39	0.005		grn	4.68	NM_001106050	10785372	DnaJ (Hsp40) homolog, subfamily C, member 15
Foxl2	0.75	60	0.002		grn	5.09	XM_345975	10912452	forkhead box L2
Hey2	0.82	31	0.042		grn	1.52	AY059382	10702361	hairy/enhancer-of-split related with YRPW motif 2
Hif3a	0.82	19	0.007		cyan	0.41	NM_022528	10719323	hypoxia inducible factor 3, alpha subunit
Nfrkb	0.78	142	0.002		blu	6.61	NM_001108133	10908833	nuclear factor related to kappa B binding protein
Steap4	1.86	99	0.043		ylw	3.31	NM_001044265	10860597	STEAP family member 4
Stxbp5l	0.81	21	0.005		red	2.93	XM_221424	10751362	syntaxin binding protein 5-like
Tcf3	0.79	49	0.010		trq	1.21	NM_001107865	10863275	transcription factor 3
Trerf1	0.77	48	0.001		brw	3.94	NM_001108199	10926351	transcriptional regulating factor 1
Tle1	0.82	44	0.026		blu	8.09	BC169119	10877644	transducin-like enhancer of split 1 (E(sp1) homolog, Drosophila)
Tp53i11	0.83	27	0.008		brw	4.52	NM_001107749	10838056	tumor protein p53 inducible protein 11
Znf296	0.80	35	0.001		grn	5.48	XM_001075949	10812118	zinc finger protein 296
Zfp57	0.81	240	0.022		grn	5.15	NM_213565	10830888	zinc finger protein 57
Znf575	0.81	13	0.004		ylw	6.77	NM_001107489	10719671	zinc finger protein 575
Znf839	0.78	22	0.022		grn	1.93	XM_001054487	10887230	zinc finger protein 839
Zfp862	0.82	15	0.027		brw	6.05	XM_001054093	10855348	zinc finger protein 862
Zdhhc2	0.76	79	0.004		blu	4.47	NM_145096	10791935	zinc finger, DHHC-type containing 2
**Translation & Protein Modification**									
Oas1d	0.59	85	0.006		brw	6.27	NM_001009379	10762226	2'-5' oligoadenylate synthetase 1D
Cpeb2	0.83	33	0.030		grn	0.98	NM_001108361	10777290	cytoplasmic polyadenylation element binding protein 2
Lsm7	1.23	34	0.024		grn	2.29	NM_001108732	10893684	LSM7 homolog, U6 small nuclear RNA associated (S. cerevisiae)

Rpl7a	0.80	103	0.009		blc	3.83	NM_001114391	10753737	ribosomal protein L7a
Rbm38	0.81	41	0.034		grn	4.12	NM_001108965	10842590	RNA binding motif protein 38
Sfrs12ip1	0.83	56	0.032		red	0.81	NM_001008373	10812865	SFRS12-interacting protein 1
RGD1562055	1.34	30	0.043		grn	4.35	XM_212651	10731416	similar to ribosomal protein L31
LOC299214	0.78	16	0.013		mbl	2.29	XM_001061760	10936432	similar to WW domain binding protein 11
Sf3b5	1.23	50	0.037		blu	3.13	NM_001126092	10701797	splicing factor 3b, subunit 5
**Miscellaneous & Unknown**									
Aff3	0.80	39	0.021		pink	2.84	XM_343559	10927562	AF4/FMR2 family, member 3
Fam105a	1.29	43	0.000		grn	1.26	BC107649	10822107	family with sequence similarity 105, member A
Gramd3	1.21	66	0.000		blu	7.01	NM_001014011	10801794	GRAM domain containing 3
LOC680214	0.55	123	0.027		red	7.23	XM_001056138	10937233	hypothetical protein LOC680214
LOC688916	0.68	31	0.008		gr	0.00	XM_001065790	10861658	hypothetical protein LOC688916
LOC689959	1.23	12	0.002		grn	1.71	NM_001109559	10917285	hypothetical protein LOC689959
Pol3s	0.83	14	0.042		blu	4.36	NM_001109156	10726070	polyserase 3
Sh2d4a	1.37	53	0.022		gr	0.00	NM_001012048	10791264	SH2 domain containing 4A
RGD1311307	0.78	38	0.030		grn	5.78	BC099145	10794800	similar to 1300014I06Rik protein
RGD1310110	0.72	87	0.017		grn	7.80	NM_001108374	10783022	similar to 3632451O06Rik protein
RGD1308023	0.77	36	0.000	+	brw	11.63	XR_006437	10850459	similar to CG5521-PA
RGD1308616	0.81	40	0.012		pink	1.87	XM_342892	10879225	similar to KIAA0467 protein
RGD1309285	0.82	72	0.005		pink	2.25	XM_221028	10739003	similar to KIAA1636 protein
Ttc6	0.70	11	0.034		blu	3.96	XM_216713	10884719	tetratricopeptide repeat domain 6
Tmem176a	1.54	344	0.010		grlw	2.40	NM_001039008	10855416	transmembrane protein 176A
Tmem97	0.81	103	0.035		red	3.41	NM_001008334	10745266	transmembrane protein 97
Trim2	1.99	144	0.049		grlw	2.44	NM_001108552	10824017	tripartite motif-containing 2
Vgll3	0.79	14	0.030		mgt	0.72	XM_573239	10749839	vestigial like 3 (Drosophila)
**EST's**									
RGD1565616	0.83	63	0.001		brw	7.67	NM_001109206	10849967	RGD1565616
LOC688296	0.78	151	0.031		cyan	9.54	XM_001081771	10740018	similar to chromosome 17 open reading frame 27
LOC688296	0.74	83	0.044		cyan	8.05	XM_001081771	10740016	similar to chromosome 17 open reading frame 27
RGD1561557	0.77	58	0.039		blc	9.76	NM_001108929	10832273	similar to chromosome 21 open reading frame 29
RGD1306936	1.28	99	0.036		pink	3.46	NM_001106593	10855462	similar to chromosome 7 open reading frame 30
RGD1563365	0.81	357	0.039		red	9.90	XM_001079678	10901403	similar to DNA segment, Chr 10, Wayne State University 102, expressed
RGD1305664	0.82	19	0.015		trq	7.33	BC089950	10743626	similar to KIAA0672 gene product
RGD1563349	0.70	29	0.002		brw	14.69	NM_001109345	10860531	similar to RIKEN cDNA 9330182L06
MGC72627	0.82	14	0.038		gr	0.00	BC061963	10853407	similar to RIKEN cDNA A330021E22
RGD1312026	0.80	139	0.017		grn	0.99	NM_001108149	10917703	similar to RIKEN cDNA C230081A13
	1.43	26	0.032		grlw	0.78	1872478	10739913	
	0.81	154	0.006		pink	1.47	15706446	10851813	
	0.80	37	0.019		blu	0.12	37182531	10864272	
	1.51	294	0.046		grn	26.60	AB014877	10886938	
	1.44	1015	0.041		grn	32.50	AB014877	10886974	
	1.51	295	0.044		grn	26.67	AB014879	10886876	
	1.44	1054	0.038		grn	32.60	AB014879	10886892	
	1.34	815	0.048		grn	25.54	AB014879	10886902	
	1.44	1054	0.038		grn	32.60	AB014879	10886904	
	1.44	1054	0.038		grn	32.60	AB014879	10886910	
	1.44	1054	0.038		grn	32.60	AB014879	10886916	
	1.44	1054	0.038		grn	32.60	AB014879	10886922	

	1.45	985	0.035		grn	32.93	AB014879	10886928	
	1.44	1054	0.038		grn	32.60	AB014879	10886934	
	1.44	1054	0.038		grn	32.60	AB014879	10886940	
	1.44	1054	0.038		grn	32.60	AB014879	10886950	
	1.44	1054	0.038		grn	32.60	AB014879	10886956	
	1.45	985	0.035		grn	32.93	AB014879	10886960	
	1.44	1054	0.038		grn	32.60	AB014879	10886966	
	1.34	815	0.048		grn	25.54	AB014879	10886978	
	1.44	1054	0.038		grn	32.60	AB014879	10886980	
	1.44	1054	0.038		grn	32.60	AB014879	10886984	
	1.44	1054	0.038		grn	32.60	AB014879	10886990	
	1.44	1054	0.038		grn	32.60	AB014879	10886992	
	1.44	1054	0.038		grn	32.60	AB014879	10886996	
	1.44	1054	0.038		grn	32.60	AB014879	10887002	
	1.44	1054	0.038		grn	32.60	AB014879	10887006	
	1.44	1054	0.038		grn	32.60	AB014879	10887012	
	1.56	97	0.044		grn	13.36	AB014881	10886870	
	1.50	53	0.049		grn	12.37	AB014881	10886874	
	0.73	29	0.001		grn	0.86	ENSRNOT0000<br>0006441	10840318	
	0.83	57	0.003		gr	0.00	ENSRNOT0000<br>0006709	10884356	
	0.76	26	0.011		mbl	1.48	ENSRNOT0000<br>0025383	10920986	
	0.80	12	0.018		grn	3.16	ENSRNOT0000<br>0027312	10734264	
	0.81	25	0.004		brw	3.76	ENSRNOT0000<br>0030218	10890340	
	0.75	717	0.022		red	10.79	ENSRNOT0000<br>0030609	10918833	
	0.74	62	0.005		blc	1.30	ENSRNOT0000<br>0036104	10805524	
	1.34	87	0.038		blu	2.30	ENSRNOT0000<br>0037181	10819500	
	0.83	17	0.004		gr	0.00	ENSRNOT0000<br>0037615	10708693	
	0.67	92	0.042		blc	8.25	ENSRNOT0000<br>0038922	10751923	
	0.81	13	0.041		blc	1.31	ENSRNOT0000<br>0041345	10747147	
	0.75	54	0.002		grn	4.61	ENSRNOT0000<br>0044547	10759431	
	0.83	27	0.008		grn	5.99	ENSRNOT0000<br>0047859	10867629	
	0.82	44	0.004		grn	4.13	ENSRNOT0000<br>0048419	10759427	
	1.21	24	0.028		mgt	1.02	ENSRNOT0000<br>0051790	10852187	
	1.51	168	0.043		grn	26.13	ENSRNOT0000<br>0052486	10886994	
	1.50	273	0.028		grn	16.60	ENSRNOT0000<br>0052657	10886880	
	1.39	655	0.023		grn	26.46	ENSRNOT0000<br>0052666	10886850	
	0.78	221	0.024		mbl	3.84	ENSRNOT0000<br>0052720	10803841	
	0.79	16	0.014		blc	1.65	ENSRNOT0000<br>0052886	10791563	
	0.72	28	0.005		blc	0.15	ENSRNOT0000<br>0053042	10937568	
	1.47	415	0.009		grn	24.60	ENSRNOT0000<br>0053070	10886848	
	0.80	23	0.037		mbl	2.83	ENSRNOT0000<br>0053248	10868277	
	1.22	63	0.026		grn	2.93	ENSRNOT0000<br>0053459	10765038	
	0.69	48	0.042		blc	6.60	ENSRNOT0000<br>0053504	10717139	
	0.79	11	0.026		brw	5.67	ENSRNOT0000<br>0053846	10702161	
	1.76	85	0.033		grn	13.76	ENSRNOT0000	10886860	

							0053852		
	0.83	18	0.043		grn	0.59	ENSRNOT0000 0053925	10755670	
	1.47	271	0.002		grn	20.19	ENSRNOT0000 0053966	10886930	
	1.41	309	0.018		grn	17.95	ENSRNOT0000 0054009	10886856	
	0.71	27	0.002		mbl	1.64	ENSRNOT0000 0054024	10904356	
	1.35	104	0.043		grn	2.42	ENSRNOT0000 0054027	10765034	
	1.46	702	0.039		grn	22.86	ENSRNOT0000 0054282	10886884	
	1.52	371	0.022		grn	28.37	ENSRNOT0000 0054324	10917516	
	0.83	16	0.016		mbl	3.86	ENSRNOT0000 0054413	10725041	
	0.76	21	0.020		blc	0.15	ENSRNOT0000 0054484	10768132	
	0.82	28	0.016		mbl	3.94	ENSRNOT0000 0054509	10795642	
	0.82	28	0.016		mbl	3.94	ENSRNOT0000 0054509	10795644	
	0.71	57	0.005		mbl	3.42	ENSRNOT0000 0055293	10747175	
	0.71	20	0.001		blc	1.54	ENSRNOT0000 0055728	10898728	
	1.60	229	0.020		grlw	1.57	ENSRNOT0000 0055984	10873027	
	0.82	50	0.009		blc	1.13	ENSRNOT0000 0056605	10721011	
	0.69	28	0.002		brw	2.82	ENSRNOT0000 0058170	10823117	
	0.68	18	0.000		blu	1.99	ENSRNOT0000 0058699	10788685	
	0.83	13	0.008		blu	1.25	ENSRNOT0000 0059236	10754480	
	0.78	16	0.006		blc	2.30	ENSRNOT0000 0059314	10769170	
	0.75	42	0.000		brw	1.45	ENSRNOT0000 0060361	10889368	
	0.71	19	0.048		mgt	0.16	ENSRNOT0000 0061126	10875764	
	0.82	73	0.021		red	0.32	ENSRNOT0000 0062076	10859857	
	0.76	21	0.043		blc	10.83	ENSRNOT0000 0062085	10843159	
	0.81	15	0.048		trq	3.84	GENSCAN0000 0008908	10904583	
	0.70	299	0.012		cyan	9.05	GENSCAN0000 0011985	10739986	
	1.29	46	0.001		ylw	4.15	rno-mir-21	10745931	MI0000850 Rattus norvegicus miR-21 stem-loop
	0.70	44	0.000		ylw	0.29	rno-mir-32	10876933	MI0000873 Rattus norvegicus miR-32 stem-loop
	0.75	37	0.016		slm	0.30	rno-mir-342	10886771	MI0000626 Rattus norvegicus miR-342 stem-loop
	0.82	12	0.021		mbl	4.13	rno-mir-672	10938856	MI0006157 Rattus norvegicus miR-672 stem-loop
	0.70	14	0.045		mbl	1.76	rno-mir-9-1	10816485	MI0000838 Rattus norvegicus miR-9-1 stem-loop

H. Genes influenced by treatment with platelet-derived growth factor alfa b (PDGRab) - 275 probe sets									
**Apoptosis**									
**Gene Symbol**	**GF/Con Ratio**	**mean_ diff***	**t-test p-value**	**Short 55 list**	**Module ****	**k.in *****	**GenBank/ Transcript ID**	**Probe Set ID**	**Gene Title/Description**
Moap1-ps1	1.20	27	0.035		trq	0.84	XM_001073080	10795986	modulator of apoptosis 1, pseudogene 1
Nradd	1.37	83	0.023		pink	4.51	NM_139259	10920461	neurotrophin receptor associated death domain
Pawr	0.78	75	0.012		trq	21.19	NM_033485	10895337	PRKC, apoptosis, WT1, regulator
**Cytoskeleton & ECM**									
Cdh1	0.78	37	0.018		mgt	0.49	NM_031334	10807542	cadherin 1
Cdh13	0.81	42	0.021		grn	2.29	NM_138889	10808274	cadherin 13
Cgnl1	0.79	84	0.022		blu	2.81	NM_001108164	10918600	cingulin-like 1
Copz2	1.26	147	0.010		ylw	2.02	NM_001108294	10737763	coatomer protein complex, subunit zeta 2
Cntn3	0.70	70	0.016		blu	1.50	NM_019329	10864481	contactin 3 (plasmacytoma associated)
Clec11a	1.22	31	0.022		ylw	6.26	NM_001012459	10721339	C-type lectin domain family 11, member a
Esm1	1.25	13	0.049		gr	0.00	NM_022604	10813107	endothelial cell-specific molecule 1
Emp3	1.29	537	0.003		pink	7.23	NM_030847	10721962	epithelial membrane protein 3
Itm2a	1.29	166	0.043		pink	0.39	NM_001025712	10938995	integral membrane protein 2A
Lmna	1.27	102	0.000		ylw	11.16	NM_001002016	10824322	lamin A
Plp2	1.39	564	0.027		pink	5.43	NM_207601	10932534	proteolipid protein 2 (colonic epithelium-enriched)
Rpa3	0.82	57	0.011		gr	0.00	NM_001106584	10860996	replication protein A3
RGD1560248	0.83	126	0.006		trq	14.62	XM_225971	10836212	similar to formin-like 2 isoform B
LOC681362	0.77	183	0.018		trq	12.71	XM_001061409	10796679	similar to p130Cas-associated protein (p140Cap) (SNAP-25-interacting protein) (SNIP)
Sptbn2	0.81	13	0.016		blu	2.50	NM_019167	10712889	spectrin, beta, non-erythrocytic 2
Tspan5	0.83	107	0.044		trq	9.91	NM_001004090	10819442	tetraspanin 5
Tuba1a	1.35	659	0.019		ylw	2.78	NM_022298	10907086	tubulin, alpha 1A
Tuba1c	1.39	446	0.034		ylw	4.99	NM_001011995	10899055	tubulin, alpha 1C
Tubb2b	1.29	18	0.036		grn	1.62	NM_001013886	10794824	tubulin, beta 2b
**Development**									
Cobll1	0.74	57	0.006		blu	1.63	NM_001107733	10845767	Cobl-like 1
Dpt	1.35	200	0.012		gr	0.00	NM_001105965	10765280	dermatopontin
Nptx2	0.70	44	0.008		mgt	2.72	NM_001034199	10760290	neuronal pentraxin 2
Rai2	0.72	118	0.001		blu	0.83	NM_001109316	10937834	retinoic acid induced 2
Sema7a	1.41	91	0.001		ylw	6.13	NM_001108153	10910406	sema domain, immunoglobulin domain (Ig), and GPI membrane anchor, (semaphorin) 7A
Sema6d	0.83	114	0.013		trq	21.98	NM_001107768	10839327	sema domain, transmembrane domain (TM), and cytoplasmic domain, (semaphorin) 6D
LOC685792	0.79	98	0.018		blc	12.63	XP_576810	10861620	similar to Discs large homolog 5 (Placenta and prostate DLG) (Discs large protein P-dlg)
LOC501091	0.78	128	0.039		blc	10.33	XP_576810	10921318	similar to Discs large homolog 5 (Placenta and prostate DLG) (Discs large protein P-dlg)
LOC680326	0.81	94	0.018		blc	9.26	XP_576810	10921311	similar to Discs large homolog 5 (Placenta and prostate DLG) (Discs large protein P-dlg)

LOC680326	0.78	17	0.032		blc	9.03	XM_001065189	10772840	similar to Discs large homolog 5 (Placenta and prostate DLG) (Discs large protein P-dlg)
LOC680326	0.82	107	0.043		blc	9.98	XP_576810	10921322	similar to Discs large homolog 5 (Placenta and prostate DLG) (Discs large protein P-dlg)
LOC501091	0.81	109	0.034		blc	11.30	XM_573609	10772857	similar to Discs large homolog 5 (Placenta and prostate DLG) (Discs large protein P-dlg)
LOC691093	1.27	29	0.007		pink	2.83	NM_001109621	10900831	similar to gene trap ROSA b-geo 22
Tor3a	1.21	64	0.000		red	4.44	NM_001009683	10768998	torsin family 3, member A
Zpbp	0.76	63	0.047		trq	15.49	NM_001025139	10778472	zona pellucida binding protein
**Electron Transport**									
Cyp17a1	0.72	12	0.031		mgt	0.77	NM_012753	10730599	cytochrome P450, family 17, subfamily a, polypeptide 1
**Epigenetics**									
Adarb1	0.81	13	0.047		mgt	2.31	NM_012894	10829367	adenosine deaminase, RNA-specific, B1
Apeg3	0.83	33	0.033		trq	6.43	NM_001034160	10718666	antisense paternally expressed gene 3
Hist2h3c2	1.26	567	0.006		pink	5.26	NM_001107698	10817537	histone cluster 2, H3c2
Hist2h3c2	1.28	105	0.025		pink	5.91	NM_001107698	10825144	histone cluster 2, H3c2
Hist2h3c2	1.28	105	0.025		pink	5.91	NM_001107698	10817535	histone cluster 2, H3c2
Kazald1	1.28	13	0.045		ylw	2.89	NM_001033064	10715667	Kazal-type serine peptidase inhibitor domain 1
LOC308670	0.71	178	0.000		mgt	0.92	AB191343	10707548	pink-eyed dilution
Pygo1	0.81	31	0.029		cyan	0.23	XM_001079880	10911531	pygopus 1
Tbx3	1.25	16	0.001		ylw	0.26	NM_181638	10762378	T-box 3
**Growth Factors, Cyto-, and Chemokines**									
Angptl2	1.38	144	0.018		ylw	7.76	NM_133569	10835654	angiopoietin-like 2
Cort	1.23	111	0.000		ylw	5.74	NM_012835	10881671	cortistatin
Dlk1	1.70	199	0.009		ylw	3.80	NM_053744	10886806	delta-like 1 homolog (Drosophila)
Lass1	1.21	12	0.000		ylw	12.61	NM_001044230	10791011	LAG1 homolog, ceramide synthase 1
Nrgn	1.52	35	0.000	+	ylw	16.11	NM_024140	10916228	neurogranin
Npy	1.43	53	0.005		grn	1.77	NM_012614	10855506	neuropeptide Y
Vegfa	1.50	134	0.011		prp	5.57	NM_031836	10921772	vascular endothelial growth factor A
**Immune Response**									
C7	1.65	342	0.010		grlw	0.46	AF309948	10821666	complement component 7
Cd80	1.20	13	0.003		grlw	0.15	NM_012926	10754176	Cd80 molecule
Hmcn1	0.81	104	0.010		ylw	3.74	XM_222716	10768426	hemicentin 1
Igsf1	0.78	17	0.002		ylw	1.99	NM_175763	10939668	immunoglobulin superfamily, member 1
PRP-2	0.80	20	0.002		gr	0.00	BC101875	10748077	proline-rich protein
**Metabolism & Transport**									
Nt5dc3	1.21	11	0.004		gr	0.00	NM_001134887	10894660	5'-nucleotidase domain containing 3
Aprt	1.24	46	0.018		grn	2.92	NM_001013061	10811651	adenine phosphoribosyl transferase
Atp5g2	1.30	298	0.027		pink	6.21	NM_133556	10907634	ATP synthase, H+ transporting, mitochondrial F0 complex, subunit C2 (subunit 9)
Atp5g2	1.21	262	0.043		pink	7.39	NM_133556	10812587	ATP synthase, H+ transporting, mitochondrial F0 complex, subunit C2 (subunit 9)
Ddt	1.24	96	0.001		prp	1.05	NM_024131	10832551	D-dopachrome tautomerase
Dnase1l1	1.25	36	0.000		prp	1.81	NM_001014223	10936076	deoxyribonuclease 1-like 1
Eno1	1.34	1181	0.038	+	prp	8.90	NM_012554	10824780	enolase 1, (alpha)
Eno1	1.37	1179	0.046	+	prp	8.99	NM_012554	10874152	enolase 1, (alpha)
Eno1	1.36	1144	0.048	+	prp	8.99	NM_012554	10836956	enolase 1, (alpha)

Galk1	1.30	46	0.024		ylw	8.34	NM_001008282	10749061	galactokinase 1
Grid2	0.80	16	0.004		grn	0.33	NM_024379	10855950	glutamate receptor, ionotropic, delta 2
Gapdh	1.29	1405	0.030		prp	8.35	NM_017008	10905817	glyceraldehyde-3-phosphate dehydrogenase
Gapdh	1.23	1105	0.044		prp	7.16	NM_017008	10754880	glyceraldehyde-3-phosphate dehydrogenase
Hhatl	1.36	23	0.042	+	ylw	15.74	NM_001106868	10914424	hedgehog acyltransferase-like
Loxl3	1.26	18	0.038		ylw	3.11	NM_001107866	10856545	lysyl oxidase-like 3
Pgk1	1.70	582	0.040		prp	8.61	NM_053291	10934610	phosphoglycerate kinase 1
Plbd1	0.65	37	0.006		brw	4.27	NM_001013927	10866459	phospholipase B domain containing 1
Pusl1	1.24	44	0.017		prp	0.25	NM_001108699	10882238	pseudouridylate synthase-like 1
Qprt	1.34	28	0.015		pink	1.93	NM_001009646	10725947	quinolinate phosphoribosyltransferase
Rcn3	1.27	71	0.047		ylw	6.12	NM_001008694	10721641	reticulocalbin 3, EF-hand calcium binding domain
LOC682697	1.26	35	0.000		prp	0.86	XR_007968	10841064	similar to Alpha-enolase (2-phospho-D-glycerate hydro-lyase) (Non-neural enolase) (NNE) (Enolase 1)
LOC361841	0.82	12	0.047		blc	10.08	XR_007137	10830022	similar to glyceraldehyde-3-phosphate dehydrogenase
LOC366054	1.23	13	0.004		prp	0.46	XR_008392	10845378	similar to Glyceraldehyde-3-phosphate dehydrogenase (GAPDH) (38 kDa BFA-dependent ADP-ribosylation substrate) (BARS-38)
LOC304240	0.83	26	0.013		blc	8.60	XR_006695	10759838	similar to N-terminal acetyltransferase complex ARD1 subunit homolog A
LOC304240	0.83	25	0.016		blc	9.25	XR_006695	10759840	similar to N-terminal acetyltransferase complex ARD1 subunit homolog A
LOC685152	1.20	47	0.001		ylw	8.65	XM_001073641	10824550	similar to Probable phospholipid-transporting ATPase ID (ATPase class I type 8B member 2)
LOC691482	0.82	25	0.020		blc	4.77	XR_007125	10921309	similar to Reticulocalbin-1 precursor
Smpx	0.80	22	0.012		gr	0.00	NM_053395	10938060	small muscle protein, X-linked
Slc17a5	1.24	37	0.013		prp	1.94	NM_001009713	10918848	solute carrier family 17 (anion/sugar transporter), member 5
Slc26a8	0.82	26	0.027		blu	1.65	NM_001107614	10831976	solute carrier family 26, member 8
Slc29a1	1.27	64	0.033	+	ylw	15.45	NM_031684	10921833	solute carrier family 29 (nucleoside transporters), member 1
Slc7a2	1.48	218	0.020		prp	1.05	NM_022619	10791902	solute carrier family 7 (cationic amino acid transporter, y+ system), member 2
Slc7a5	1.36	67	0.014	+	ylw	15.61	NM_017353	10811531	solute carrier family 7 (cationic amino acid transporter, y+ system), member 5
Sc5dl	1.26	94	0.008		pink	1.21	NM_053642	10916534	sterol-C5-desaturase (ERG3 delta-5-desaturase homolog, S. cerevisiae)-like
Stoml1	1.20	25	0.004		gr	0.00	XM_236297	10910450	stomatin-like 1
Tpi1	1.45	539	0.045		prp	6.43	NM_022922	10798021	triosephosphate isomerase 1
Uap1l1	1.26	17	0.006		prp	2.40	NM_001134516	10843438	UDP-N-acteylglucosamine pyrophosphorylase 1-like 1
**Proteolysis**									
Adamtsl2	0.80	21	0.041		gr	0.00	XM_231125	10834670	ADAMTS-like 2

Usp27x	0.79	23	0.004		pink	1.37	XM_228777	10932570	ubiquitin specific peptidase 27, X-linked
**Receptors & Binding Proteins**									
Abca2	0.80	55	0.046		trq	23.37	NM_024396	10834241	ATP-binding cassette, sub-family A (ABC1), member 2
Ednra	1.32	173	0.007		red	2.21	NM_012550	10807022	endothelin receptor type A
Kdelr2	1.24	258	0.001		pink	3.72	NM_001013122	10760397	KDEL (Lys-Asp-Glu-Leu) endoplasmic reticulum protein retention receptor 2
Kdelr3	1.33	48	0.038		ylw	5.94	NM_001127546	10905453	KDEL (Lys-Asp-Glu-Leu) endoplasmic reticulum protein retention receptor 3
Leng4	1.25	55	0.004		ylw	4.90	NM_001134978	10703715	leukocyte receptor cluster (LRC) member 4
Lpar4	1.24	74	0.003		ylw	1.64	NM_001106940	10934631	lysophosphatidic acid receptor 4
Npr3	0.77	14	0.005		brw	1.42	NM_012868	10821991	natriuretic peptide receptor C/guanylate cyclase C (atrionatriuretic peptide receptor C)
Ngfr	0.79	31	0.041		mgt	4.85	NM_012610	10746538	nerve growth factor receptor (TNFR superfamily, member 16)
Olr922	0.64	15	0.040		blc	11.68	NM_001000705	10900037	olfactory receptor 922
Oprs1	1.30	72	0.015		ylw	2.50	NM_030996	10876185	opioid receptor, sigma 1
Ptx3	1.21	22	0.047		brw	3.59	NM_001109536	10815785	pentraxin related gene
Pear1	1.25	40	0.001		ylw	7.15	NM_001134959	10824165	platelet endothelial aggregation receptor 1
Paqr7	1.25	11	0.013		ylw	3.44	NM_001034081	10872808	progestin and adipoQ receptor family member VII
P2ry1	1.30	15	0.006		mgt	0.82	NM_012800	10815652	purinergic receptor P2Y, G-protein coupled 1
LOC500726	0.83	38	0.039		trq	9.75	XM_576105	10887516	similar to KARP-1 binding protein 1
RGD1560871	0.83	56	0.012		trq	13.87	XM_001072622	10863960	similar to plexin 1
LOC317546	0.80	78	0.009		pink	2.45	XR_005973	10933903	similar to TAK1-binding protein 3 isoform 1
Vom2r65	0.75	17	0.027		mbl	1.64	NM_001099654	10770892	vomeronasal 2 receptor, 65
**Signaling**									
Calml3	0.61	101	0.006		mgt	6.36	NM_001012054	10796090	calmodulin-like 3
Cdc42ep4	1.20	16	0.023		ylw	0.34	NM_001107063	10748797	CDC42 effector protein (Rho GTPase binding) 4
Cyr61	1.28	51	0.013		ylw	9.57	NM_031327	10827231	cysteine-rich, angiogenic inducer, 61
Defb24	1.32	12	0.000		ylw	8.60	NM_001037508	10850812	defensin beta 24
Diras2	1.39	21	0.012		ylw	4.21	XM_225214	10794240	DIRAS family, GTP-binding RAS-like 2
Dok6	0.72	40	0.036		blu	1.33	XM_001063746	10805605	docking protein 6
Dyrk1b	0.83	18	0.001		trq	2.32	NM_001107496	10705494	dual-specificity tyrosine-(Y)-phosphorylation regulated kinase 1b
Elmod2	1.24	71	0.036		trq	3.30	NM_001109506	10806873	ELMO/CED-12 domain containing 2
Enah	0.79	79	0.025		blu	1.74	NM_001012150	10770412	enabled homolog (Drosophila)
Fkbp14	1.21	78	0.002		pink	0.96	NM_001013210	10862643	FK506 binding protein 14
Freq	1.31	13	0.048		ylw	6.66	NM_024366	10835292	frequenin homolog (Drosophila)
Gnao1	0.82	10	0.003		pink	0.29	NM_017327	10809428	guanine nucleotide binding protein (G protein), alpha activating activity polypeptide O
Hipk2	0.79	112	0.004		trq	6.69	NM_001108622	10861997	homeodomain interacting protein kinase 2
Itgbl1	0.83	11	0.037		gr	0.00	NM_001017505	10782187	integrin, beta-like 1
Minpp1	0.80	34	0.000		gr	0.00	NM_019263	10719148	multiple inositol polyphosphate histidine phosphatase 1
Pde3a	0.81	69	0.032		grn	4.61	NM_017337	10859494	phosphodiesterase 3A, cGMP inhibited
Pfkl	1.27	63	0.040		prp	3.59	NM_013190	10829290	phosphofructokinase, liver

Ptprv	1.23	28	0.004		red	1.57	NM_033099	10767876	protein tyrosine phosphatase, receptor type, V
Rabac1	1.31	182	0.004		pink	9.12	NM_031774	10719722	Rab acceptor 1 (prenylated)
Rem1	1.47	19	0.035	+	ylw	14.52	NM_001025753	10840861	RAS (RAD and GEM)-like GTP-binding 1
Rhoj	1.29	110	0.000		pink	0.73	NM_001008320	10885299	ras homolog gene family, member J
Ramp1	1.40	44	0.010		ylw	5.20	NM_031645	10925365	receptor (G protein-coupled) activity modifying protein 1
Rgs4	1.50	34	0.022		ylw	7.01	NM_017214	10769672	regulator of G-protein signaling 4
Rgs5	0.82	15	0.034		brw	5.87	NM_019341	10765437	regulator of G-protein signaling 5
Rara	0.81	90	0.048		trq	11.77	NM_031528	10738130	retinoic acid receptor, alpha
Ssr4	1.28	229	0.008		pink	8.99	NM_017199	10936013	signal sequence receptor, delta
Sipa1l3	0.78	28	0.006		trq	6.82	NM_001013066	10720489	signal-induced proliferation-associated 1 like 3
LOC679206	0.42	81	0.046		blc	11.83	XM_001055219	10718197	similar to FERM domain containing 1
S1pr2	1.24	46	0.007		ylw	12.25	NM_017192	10915483	sphingosine-1-phosphate receptor 2
S1pr3	1.37	119	0.004		prp	0.80	XM_225216	10797566	sphingosine-1-phosphate receptor 3
Ubash3b	1.67	69	0.044	+	ylw	16.87	XM_236104	10916476	ubiquitin associated and SH3 domain containing, B
Wisp1	1.20	12	0.021		red	0.13	NM_031716	10896935	WNT1 inducible signaling pathway protein 1
**Transcription**									
Ddx60	0.78	110	0.035		mgt	3.52	XP_001070139	10791394	DEAD (Asp-Glu-Ala-Asp) box polypeptide 60
Ddx60	0.77	155	0.029		mgt	3.73	XP_001070139	10791358	DEAD (Asp-Glu-Ala-Asp) box polypeptide 60
Ddx60	0.79	103	0.040		mgt	3.65	XP_001070139	10787889	DEAD (Asp-Glu-Ala-Asp) box polypeptide 60
Dnajc21	0.83	66	0.038		pink	2.61	NM_138856	10821900	DnaJ (Hsp40) homolog, subfamily C, member 21
Gli3	0.83	57	0.005		trq	7.14	AB073718	10798706	GLI-Kruppel family member GLI3
Klf7	1.32	48	0.001		ylw	4.21	NM_001108800	10928524	Kruppel-like factor 7 (ubiquitous)
Myg1	1.21	49	0.002		pink	4.95	NM_001005545	10899537	melanocyte proliferating gene 1
Nudt4	1.34	187	0.008		ylw	4.13	NM_053598	10902003	nudix (nucleoside diphosphate linked moiety X)-type motif 4
Prdm10	0.82	53	0.040		pink	2.52	XM_001056255	10908827	PR domain containing 10
MGC94600	1.21	190	0.047		ylw	8.05	NM_001006989	10913487	scotin
Sertad4	1.25	34	0.002		ylw	0.57	NM_001108351	10770759	SERTA domain containing 4
Set	0.83	84	0.026		gr	0.00	NM_001012504	10749869	SET nuclear oncogene
RGD1565550	0.56	30	0.044		blc	8.22	XM_574776	10830270	similar to double homeobox, 4
LOC688583	0.83	13	0.041		gr	0.00	XM_001067503	10853238	similar to High mobility group protein 4 (HMG-4) (High mobility group protein 2a) (HMG-2a)
RGD1562997	0.77	79	0.005		pink	2.29	XM_001073245	10800323	similar to Transcription initiation factor TFIID 105 kDa subunit (TAFII-105)
LOC681381	0.83	12	0.007		pink	0.84	XM_001061491	10920787	similar to Zinc finger CW-type PWWP domain protein 1 homolog
LOC687769	0.79	18	0.023		blc	0.51	XM_001080061	10706240	similar to zinc finger protein 11B
Smad9	0.75	35	0.014		mgt	3.36	NM_138872	10815436	SMAD family member 9
Txnip	1.26	941	0.001		prp	2.45	NM_001008767	10817552	thioredoxin interacting protein
Zfp385b	0.81	17	0.009		ylw	2.23	NM_001107736	10846652	zinc finger protein 385B
Znf521	0.79	83	0.004		gr	0.00	NM_001107403	10803276	zinc finger protein 521

Znf704	0.78	37	0.001		pink	1.89	XM_001056858	10814310	zinc finger protein 704
**Translation & Protein Modification**									
Eif4e3	1.26	12	0.011		prp	1.31	NM_001106612	10864425	eukaryotic translation initiation factor 4E member 3
Mto1	0.83	31	0.001		pink	1.73	NM_001106841	10911813	mitochondrial translation optimization 1 homolog (S. cerevisiae)
Padi4	0.82	19	0.045		mgt	1.40	NM_017227	10880960	peptidyl arginine deiminase, type IV
Rnasek	1.23	307	0.001		pink	4.06	NM_001137561	10744391	ribonuclease, RNase K
Rpl31	1.21	63	0.020		pink	5.38	BC062228	10922768	ribosomal protein L31
**Miscellaneous & Unknown**									
Aff2	0.72	58	0.015		pink	1.31	XM_219832	10935739	AF4/FMR2 family, member 2
Fam105a	1.25	36	0.042		grn	1.26	BC107649	10822107	family with sequence similarity 105, member A
Gatsl3	1.23	19	0.032		pink	0.21	NM_001025128	10773838	GATS protein-like 3
LOC687502	0.70	10	0.035		blc	4.59	XM_001078856	10740560	hypothetical protein LOC687502
LOC688956	0.79	132	0.027		blc	6.31	XM_001074547	10861672	hypothetical protein LOC688956
LOC691995	1.41	120	0.000		ylw	6.34	NM_001103353	10744597	hypothetical protein LOC691995
Pqlc3	1.29	19	0.029		red	0.77	NM_001034952	10889308	PQ loop repeat containing 3
R3hdm2	0.83	79	0.038		pink	2.94	NM_001130557	10895888	R3H domain containing 2
Rcsd1	0.82	14	0.040		blu	0.32	NM_001108349	10769547	RCSD domain containing 1
Scg3	0.74	16	0.039		blu	1.16	NM_053856	10918738	secretogranin III
LOC686661	1.21	94	0.022		pink	1.17	XM_001075174	10742868	similar to CG1998-PA
RGD1305689	1.30	185	0.047		pink	2.97	BC086323	10789740	similar to DNA segment, Chr 14, ERATO Doi 449, expressed
RGD1561503	0.78	40	0.023		gr	0.00	XM_574857	10821623	similar to hypothetical protein AN1443.2
RGD1359508	1.23	19	0.000		gr	0.00	BC088236	10815189	similar to protein C33A12.3
LOC301748	0.79	84	0.034		blc	12.89	BC098021	10772847	similar to RIKEN cDNA 1700001E04
Spryd3	1.24	17	0.000		ylw	1.94	BC105624	10907529	SPRY domain containing 3
Tmem14a	1.21	12	0.004		pink	0.64	NM_001108790	10922145	transmembrane protein 14A
Tmem178	1.48	182	0.000		ylw	8.94	NM_001004282	10882525	transmembrane protein 178
Tmem97	1.29	159	0.030		red	3.41	NM_001008334	10745266	transmembrane protein 97
Tsku	1.26	53	0.001		ylw	4.76	NM_001009965	10723822	tsukushin
Wdr75	1.24	38	0.034		pink	2.79	XM_001062449	10923127	WD repeat domain 75
Wwc2	0.80	82	0.023		mgt	4.83	NM_001109111	10788101	WW and C2 domain containing 2
**EST's**									
RGD1309634	0.82	99	0.023		pink	2.81	XM_223536	10777694	hypothetical LOC305452
LOC501092	0.77	121	0.021		blc	11.15	BC098021	10861606	hypothetical LOC501092
RGD1564058	1.27	108	0.014		pink	7.46	BC166543	10809937	similar to cDNA sequence BC056474
RGD1305664	0.78	25	0.005		trq	7.33	BC089950	10743626	similar to KIAA0672 gene product
RGD1306939	0.80	15	0.047		grn	5.80	NM_001014009	10798390	similar to mKIAA0386 protein
LOC501224	0.82	105	0.016		blc	9.15	BC079060	10772809	similar to RIKEN cDNA 2610042L04
LOC501224	0.75	117	0.018		blc	12.56	BC079060	10772815	similar to RIKEN cDNA 2610042L04
LOC501224	0.78	122	0.026		blc	13.10	BC079060	10772821	similar to RIKEN cDNA 2610042L04
LOC501224	0.77	130	0.026		blc	14.40	BC079060	10772836	similar to RIKEN cDNA 2610042L04
RGD1304563	1.38	14	0.028		ylw	13.33	NM_001106762	10892050	similar to RIKEN cDNA 4831426I19
LOC500124	0.78	14	0.035		prp	0.28	BC078992	10862505	similar to RIKEN cDNA 4921507P07
	0.74	17	0.043		brw	7.45	13542930	10765444	
	0.80	10	0.005		gr	0.00	85861187	10788014	
	1.40	42	0.000		grn	12.37	AB014881	10886874	
	0.79	99	0.038		blc	1.26	AY539927	10868302	
	0.77	49	0.048		grn	1.57	BC166431	10806514	

	0.80	14	0.013		grn	1.54	ENSRNOT00000002166	10836988	
	1.23	25	0.010		pink	0.72	ENSRNOT00000014997	10811289	
	0.80	20	0.025		blu	2.33	ENSRNOT00000018898	10745340	
	1.21	15	0.044		prp	0.63	ENSRNOT00000023364	10936751	
	0.82	51	0.015		pink	1.88	ENSRNOT00000033372	10804772	
	1.40	245	0.034		prp	8.81	ENSRNOT00000034026	10937570	
	0.83	11	0.049		trq	2.14	ENSRNOT00000034326	10856011	
	0.78	19	0.019		mgt	1.04	ENSRNOT00000038519	10883880	
	0.79	24	0.023		trq	8.33	ENSRNOT00000038929	10720516	
	1.25	194	0.023		red	8.84	ENSRNOT00000040010	10918829	
	1.25	194	0.023		red	8.84	ENSRNOT00000040010	10716982	
	1.25	194	0.023		red	8.84	ENSRNOT00000040010	10902883	
	0.80	46	0.041		blc	6.35	ENSRNOT00000041432	10890020	
	1.22	12	0.038		ylw	0.78	ENSRNOT00000042423	10938712	
	0.76	248	0.002		blc	3.83	ENSRNOT00000043289	10821067	
	0.83	38	0.028		grn	4.61	ENSRNOT00000044547	10759431	
	0.44	62	0.019		blc	1.71	ENSRNOT00000045731	10787803	
	0.73	18	0.046		trq	7.83	ENSRNOT00000048596	10890923	
	0.77	17	0.024		trq	9.00	ENSRNOT00000048596	10890904	
	1.24	186	0.024		red	8.93	ENSRNOT00000049144	10836210	
	1.27	28	0.041		pink	3.79	ENSRNOT00000049785	10798501	
	0.83	16	0.000		gr	0.00	ENSRNOT00000050822	10852195	
	0.74	39	0.008		blc	1.13	ENSRNOT00000051386	10878386	
	0.80	20	0.007		gr	0.00	ENSRNOT00000051772	10752061	
	0.57	50	0.004		mgt	1.02	ENSRNOT00000051790	10852187	
	0.62	62	0.042		blc	5.86	ENSRNOT00000052507	10722471	
	0.63	64	0.049		blc	4.85	ENSRNOT00000052661	10722437	
	0.63	64	0.049		blc	4.85	ENSRNOT00000052661	10722441	
	0.78	20	0.001		grn	0.47	ENSRNOT00000052741	10726878	
	0.78	19	0.024		blu	1.25	ENSRNOT00000052858	10733992	
	0.68	13	0.022		blc	5.18	ENSRNOT00000053067	10801381	
	1.24	20	0.001		grn	0.70	ENSRNOT00000053277	10861115	
	0.78	14	0.047		blu	2.72	ENSRNOT00000053339	10813885	
	0.60	21	0.034		blu	4.48	ENSRNOT00000053934	10722465	
	1.27	37	0.013		grn	16.81	ENSRNOT00000054130	10886890	
	0.50	52	0.046		blc	10.94	ENSRNOT00000054285	10722415	

	0.66	14	0.030		tan	0.22	ENSRNOT0000 0055218	10724341	
	0.60	120	0.003		pink	3.72	ENSRNOT0000 0056162	10872699	
	0.54	84	0.039		pink	1.06	ENSRNOT0000 0056786	10939699	
	1.29	57	0.047		ylw	0.65	ENSRNOT0000 0057104	10903674	
	0.76	248	0.002		blc	3.83	ENSRNOT0000 0057163	10705411	
	0.58	70	0.001		gr	0.00	ENSRNOT0000 0057306	10815996	
	1.57	28	0.029		blc	2.11	ENSRNOT0000 0057365	10924441	
	0.72	40	0.001		gr	0.00	ENSRNOT0000 0057420	10833416	
	0.70	35	0.000		gr	0.00	ENSRNOT0000 0059226	10927803	
	0.72	40	0.001		gr	0.00	ENSRNOT0000 0059477	10791602	
	0.66	17	0.008		grn	1.15	ENSRNOT0000 0061205	10800513	
	0.66	62	0.030		blu	13.55	ENSRNOT0000 0062861	10702192	
	0.66	62	0.030		blu	13.55	ENSRNOT0000 0062861	10739209	
	0.66	62	0.030		blu	13.55	ENSRNOT0000 0062861	10788077	
	0.66	62	0.030		blu	13.55	ENSRNOT0000 0062861	10863402	
	0.66	62	0.030		blu	13.55	ENSRNOT0000 0062861	10885957	
	0.66	62	0.030		blu	13.55	ENSRNOT0000 0062861	10922249	
	0.66	62	0.030		blu	13.55	ENSRNOT0000 0062861	10937321	
	1.32	69	0.011		blc	0.96	ENSRNOT0000 0063576	10891487	
	0.82	34	0.038		trq	9.66	GENSCAN0000 0035197	10720504	
	0.76	74	0.005		trq	9.91	GENSCAN0000 0035197	10720506	
	0.77	57	0.014		trq	7.46	GENSCAN0000 0035197	10720514	
	1.30	39	0.048		grn	7.22	K02909	10918820	
	0.76	18	0.006		blc	0.91	NM_001044260, 112984231, 68235770	10877667	
	1.22	11	0.012		ylw	2.16	rno-mir-22	10735887	MI0000851 Rattus norvegicus miR-22 stem-loop
	0.83	14	0.032		blc	5.57	U15425	10701862	

I. Genes influenced by treatment with connective tissue growth factor (CTGF) - 155 probe sets						
**Cytoskeleton & ECM**						
**Gene Symbol**	**GF/Con Ratio**	**mean_ diff***	**t-test p-value**	**GenBank/ Transcript ID**	**Probe Set ID**	**Gene Title/Description**
Cdh6	1.42	26.5	0.008	NM_012927	10822043	cadherin 6
Cilp	1.36	21.8	0.002	NM_001108161	10910926	cartilage intermediate layer protein, nucleotide pyrophosphohydrolase
Elmo2	1.21	91.1	0.007	NM_001134955	10851753	engulfment and cell motility 2
Muc16	0.78	93.4	0.029	XM_001075437	10915239	mucin 16, cell surface associated
Ncam1	1.22	32.6	0.030	NM_031521	10917183	neural cell adhesion molecule 1
Pcdhb8	1.200	21.4	0.046	NM_001014779	10801180	protocadherin beta 8
Pcdhga6	1.23	28.1	0.008	ENSRNOT00000027019	10801254	protocadherin gamma subfamily A, 6
Spon2	1.25	60.9	0.045	NM_138533	10777788	spondin 2, extracellular matrix protein
**Development**						
Crispld2	1.23	57.3	0.046	NM_138518	10808377	cysteine-rich secretory protein LCCL domain containing 2
Dppa3	0.75	201.9	0.026	NM_001047864	10858517	developmental pluripotency-associated 3
Dppa3	0.75	192.5	0.025	NM_001047864	10877673	developmental pluripotency-associated 3
Dppa3	0.76	193.9	0.022	NM_001047864	10848575	developmental pluripotency-associated 3
Hps4	1.27	33.2	0.019	NM_001107148	10806174	Hermansky-Pudlak syndrome 4 homolog (human)
Hoxa5	1.33	16.2	0.042	L03556	10862561	homeo box A5
Olfml2b	1.22	27.6	0.034	NM_001107195	10765480	olfactomedin-like 2B
Ophn1	1.26	113.1	0.036	NM_001107848	10938550	oligophrenin 1
Ott	0.828	31.5	0.034	ENSRNOT00000045110	10937272	ovary testis transcribed
Shisa2	1.21	37.9	0.014	ENSRNOT00000016826	10780850	shisa homolog 2 (Xenopus laevis)
RGD1559950	0.73	45.7	0.030	ENSRNOT00000047795	10793605	similar to STELLA
RGD1560771	0.75	135.2	0.049	ENSRNOT00000051430	10783524	similar to STELLA
Tex11	0.82	12.5	0.047	XM_576992	10938606	testis expressed 11
**Electron Transport**						
Cyp4f5	1.21	21.2	0.004	NM_173124	10894180	cytochrome P450 4F5
LOC305698	0.82	43.6	0.019	ENSRNOT00000049636	10782428	similar to Glutaredoxin-1 (Thioltransferase-1) (TTase-1)
**Epigenetics**						
Tpmt	0.81	71.8	0.028	NM_001079531	10794464	thiopurine S-methyltransferase
**Golgi Apparatus**						
Gcnt1	0.82	15.3	0.044	NM_022276	10729209	glucosaminyl (N-acetyl) transferase 1, core 2
St6gal2	1.37	34.6	0.013	AJ627626	10930711	ST6 beta-galactosamide alpha-2,6-sialyltranferase 2
**Growth Factor**						
Dll4	1.23	59.5	0.026	NM_001107760	10838813	delta-like 4 (Drosophila)
Bmp3	0.77	83.7	0.015	NM_017105	10775573	bone morphogenetic protein 3
Grem2	1.28	96.2	0.037	NM_001105974	10770117	gremlin 2, cysteine knot superfamily, homolog (Xenopus laevis)
**Immune Response**						
C1qc	1.30	95.9	0.046	NM_001008524	10880731	complement component 1, q subcomponent, C chain
LOC686809	1.29	23.7	0.037	ENSRNOT00000023451	10704840	similar to protein 7 transactivated by hepatitis B virus X antigen
Ns5atp9	0.78	52.2	0.032	NM_201418	10911010	NS5A (hepatitis C virus) transactivated protein 9
**Metabolism & Transport**						
Acacb	1.23	18.0	0.028	NM_053922	10762792	acetyl-Coenzyme A carboxylase beta
Apol9a	0.830	10.5	0.007	NM_001025066	10905179	apolipoprotein L 9a
MGC72973	0.79	202.6	0.021	NM_198776	10724319	beta-glo
Lrp1b	1.31	17.4	0.046	NM_001107843	10845010	low density lipoprotein-related protein 1B (deleted in tumors)
Lrp1b	1.32	12.2	0.030	NM_001107843	10844970	low density lipoprotein-related protein 1B (deleted in tumors)
Lrp1b	1.48	42.0	0.034	NM_001107843	10845051	low density lipoprotein-related protein 1B (deleted in tumors)
Mt2A	1.30	428.4	0.022	NM_001137564	10809399	metallothionein 2A
Ppif	1.22	116.7	0.032	NM_172243	10786163	peptidylprolyl isomerase F (cyclophilin F)
Ptgs1	1.20	15.7	0.020	NM_017043	10835817	prostaglandin-endoperoxide synthase 1

Rdh2	1.55	16.6	0.039	NM_199208	10895990	retinol dehydrogenase 2
Scamp4	1.21	85.7	0.019	NM_031725	10900478	secretory carrier membrane protein 4
LOC683538	1.202	34.4	0.046	ENSRNOT0000 0061761	10812484	similar to metaxin 3
Slc16a14	1.25	10.8	0.010	NM_001108229	10929536	solute carrier family 16, member 14 (monocarboxylic acid transporter 14)
Slc4a3	1.22	21.3	0.015	NM_017049	10924621	solute carrier family 4 (anion exchanger), member 3
Star	1.24	66.5	0.006	NM_031558	10792251	steroidogenic acute regulatory protein
LOC303140	1.27	21.3	0.044	DQ119106	10742657	up-regulator of carnitine transporter, OCTN2
**Proteolysis**						
Adamts6	1.24	64.2	0.044	NM_001108544	10812834	ADAM metallopeptidase with thrombospondin type 1 motif, 6
Rnf213	0.80	49.1	0.032	XM_001081768	10739998	ring finger protein 213
Spink5	0.82	14.9	0.025	XM_001056195	10801434	serine peptidase inhibitor, Kazal type 5
**Receptor & Binding Proteins**						
Abcc3	1.21	53.6	0.038	NM_080581	10746293	ATP-binding cassette, sub-family C (CFTR/MRP), member 3
Cnr1	1.52	50.6	0.003	NM_012784	10868186	cannabinoid receptor 1 (brain)
Crhr2	1.31	27.0	0.033	NM_022714	10862677	corticotropin releasing hormone receptor 2
Il28ra	1.24	19.4	0.026	ENSRNOT0000 0049459	10872867	interleukin 28 receptor alpha
Ntrk3	1.26	22.5	0.013	NM_019248	10722864	neurotrophic tyrosine kinase, receptor, type 3
Olr167	0.66	20.0	0.004	NM_001000173	10724409	olfactory receptor 167
Pear1	1.26	41.0	0.021	NM_001134959	10824165	platelet endothelial aggregation receptor 1
Rlbp1	0.79	12.2	0.008	NM_001106274	10722918	retinaldehyde binding protein 1
**Signaling**						
Adck5	1.203	38.2	0.014	NM_001135798	10897308	aarF domain containing kinase 5
Dtx3	1.22	20.5	0.018	ENSRNOT0000 0006815	10902952	deltex 3 homolog (Drosophila)
Frmd5	1.23	91.2	0.025	ENSRNOT0000 0046659	10849156	FERM domain containing 5
Gabrb2	1.31	34.5	0.000	NM_012957	10732824	gamma-aminobutyric acid (GABA) A receptor, beta 2
Gng11	0.79	30.9	0.000	NM_022396	10853554	guanine nucleotide binding protein (G protein), gamma 11
Mrgprb4	0.82	46.9	0.034	ENSRNOT0000 0045792	10722218	MAS-related GPR, member B4
Mapkbp1	1.25	50.7	0.046	NM_001108589	10838927	mitogen activated protein kinase binding protein 1
Ncln	1.31	57.2	0.044	NM_001014082	10893484	nicalin homolog (zebrafish)
Prrg3	1.25	30.4	0.031	ENSRNOT0000 0003233	10940085	proline rich Gla (G-carboxyglutamic acid) 3 (transmembrane)
Ppp1r16a	1.200	21.8	0.033	NM_001130566	10897347	protein phosphatase 1, regulatory (inhibitor) subunit 16A
Rabl4	0.825	46.7	0.037	NM_001130495	10905270	RAB, member of RAS oncogene family-like 4
Rgn	0.82	13.9	0.015	NM_031546	10936637	regucalcin (senescence marker protein-30)
MGC109340	0.82	376.0	0.024	BC092634	10935031	similar to Microsomal signal peptidase 23 kDa subunit (SPase 22 kDa subunit) (SPC22/23)
Smad9	1.22	43.7	0.038	NM_138872	10815436	SMAD family member 9
Tom1l2	1.22	53.9	0.006	NM_001108277	10743235	target of myb1-like 2 (chicken)
**Transcription**						
Ccdc3	1.27	25.4	0.049	ENSRNOT0000 0024110	10799548	coiled-coil domain containing 3
Ccdc72	0.79	107.9	0.027	NM_001126048	10920371	coiled-coil domain containing 72
Magoh	0.82	457.0	0.047	NM_001100536	10870733	mago-nashi homolog, proliferation-associated (Drosophila)
Mrvi1	1.31	38.4	0.004	NM_001105211	10724902	MRV integration site 1 homolog (mouse)
Mlf1	0.828	10.6	0.040	NM_001107680	10815806	myeloid leukemia factor 1
Polr3gl	1.22	27.3	0.027	NM_001109571	10825171	polymerase (RNA) III (DNA directed) polypeptide G (32kD)-like
Rbm8	0.80	16.9	0.004	DQ359102	10807685	RNA binding motif protein 8
Sec16b	1.23	10.4	0.046	NM_053571	10764891	SEC16 homolog B (S. cerevisiae)
LOC685764	0.73	17.9	0.032	XR_006289	10717104	similar to High mobility group protein 1 (HMG-1) (High mobility group protein B1) (Amphoterin) (Heparin-binding protein p30)
RGD1305673	1.26	25.1	0.030	ENSRNOT0000 0011542	10847554	similar to PR-domain protein 11
RGD1304870	0.80	727.2	0.008	XM_221241	10737399	similar to Tceb2 protein
LOC680767	0.76	288.2	0.033	XM_001058777	10760816	similar to transcription elongation factor B (SIII), polypeptide 1
Sned1	1.30	26.2	0.037	AF439715	10925526	sushi, nidogen and EGF-like domains 1
Znf23	1.22	80.9	0.025	NM_001107428	10807828	zinc finger protein 23 (KOX 16)

Zfp346	1.25	50.1	0.008	NM_001107338	10797344	zinc finger protein 346
Znf467	1.25	24.0	0.016	NM_001024327	10862398	zinc finger protein 467
**Translation and Protein Modification**						
Rps6kl1	1.22	26.2	0.001	NM_001106745	10891229	ribosomal protein S6 kinase-like 1
LOC292743	1.26	11.4	0.044	XR_009190	10705440	similar to 60S ribosomal protein L23a
LOC364390	0.79	15.6	0.045	XR_007814	10780735	similar to 60S ribosomal protein L26
LOC365070	0.76	22.0	0.026	XR_007522	10717353	similar to 60S ribosomal protein L21
LOC678896	0.82	29.6	0.021	XM_001067503	10853238	similar to High mobility group protein 4 (HMG-4) (High mobility group protein 2a) (HMG-2a)
LOC680441	0.75	13.2	0.004	XM_001058885	10864846	similar to 60S ribosomal protein L23a
LOC680988	0.80	29.8	0.007	XM_001057986	10858275	similar to ribosomal protein S12
LOC686066	0.76	74.7	0.050	ENSRNOT0000 0040570	10844181	similar to 60S ribosomal protein L38
LOC692040	0.76	10.0	0.041	XR_009143	10781954	similar to ribosomal protein S13
RGD1561195	0.73	12.6	0.027	ENSRNOT0000 0046725	10877571	similar to ribosomal protein L31
RGD1561463	0.74	15.2	0.002	XR_009002	10893436	similar to Sfrs7 protein
RGD1562427	0.80	23.9	0.028	XR_007963	10903522	similar to ribosomal protein L31
RGD1562543	0.77	47.2	0.024	XR_008876	10717067	similar to ribosomal protein L27a
RGD1565806	0.62	56.7	0.021	ENSRNOT0000 0035742	10793071	similar to 60S ribosomal protein L23a
**Miscellaneous, Unknown, and ESTs**						
LOC689545	0.77	168.5	0.028	ENSRNOT0000 0061371	10775263	hypothetical protein LOC689545
Robld3	0.71	26.2	0.034	NM_001106441	10824344	roadblock domain containing 3
RGD1311910	1.24	19.2	0.012	NM_001107373	10805384	similar to hypothetical p38 protein
RGD1307722	1.24	117.2	0.012	NM_001108730	10900201	similar to hypothetical protein MGC20700
RGD1565688	1.22	42.1	0.020	ENSRNOT0000 0027802	10780527	similar to mKIAA0323 protein
RGD1306404	1.22	39.4	0.030	NM_001106574	10859988	similar to mKIAA1402 protein
RGD1560291	0.77	220.0	0.032	ENSRNOT0000 0043793	10901121	similar to NACHT, leucine rich repeat and PYD containing 4A
RGD1304644	1.85	48.3	0.028	BC099097	10840783	similar to RIKEN cDNA 2310046K01
RGD1310686	1.25	33.5	0.030	BC085860	10731738	similar to chromosome 16 open reading frame 5
RGD1560559	0.75	83.3	0.013	XM_221310	10755281	similar to RIKEN cDNA 2310042E22
RGD1565146	1.21	27.3	0.001	AF226663	10760580	similar to hypothetical gene supported by AF226663
	0.62	43.8	0.029	ENSRNOT0000 0058939	10933576	
	0.64	51.0	0.034	ENSRNOT0000 0056786	10939699	
	0.68	53.5	0.020	ENSRNOT0000 0054734	10715250	
	0.72	22.6	0.028	ENSRNOT0000 0051208	10802371	
	0.73	43.3	0.026	K02909	10918820	
	0.73	217.9	0.013	ENSRNOT0000 0039948	10767044	
	0.75	15.1	0.011	ENSRNOT0000 0039451	10908160	
	0.75	10.3	0.021	ENSRNOT0000 0009072	10810303	
	0.75	37.2	0.036	ENSRNOT0000 0036238	10914605	
	0.76	26.5	0.015	ENSRNOT0000 0040905	10705188	
	0.76	91.8	0.007	ENSRNOT0000 0050559	10917992	
	0.77	13.8	0.018	ENSRNOT0000 0020764	10794647	
	0.77	105.7	0.038	ENSRNOT0000 0013475	10785895	
	0.79	12.7	0.003	ENSRNOT0000	10867916	

				0009117		
	0.79	10.1	0.030	ENSRNOT0000 0040940	10904601	
	0.79	51.2	0.045	ENSRNOT0000 0039879	10897891	
	0.79	19.8	0.047	ENSRNOT0000 0007262	10861224	
	0.79	10.0	0.018	ENSRNOT0000 0018011	10926819	
	0.79	13.8	0.041	GENSCAN0000 0037428	10748074	
	0.80	141.1	0.045	ENSRNOT0000 0002159	10911944	
	0.80	54.2	0.024	ENSRNOT0000 0060122	10884046	
	0.80	11.5	0.031	ENSRNOT0000 0046365	10730531	
	0.81	91.5	0.042	ENSRNOT0000 0039250	10791820	
	0.81	15.4	0.012	ENSRNOT0000 0056568	10935679	
	0.82	50.2	0.019	ENSRNOT0000 0053376	10736240	
	0.82	15.8	0.040	ENSRNOT0000 0016492	10879480	
	0.82	16.9	0.049	ENSRNOT0000 0040382	10850084	
	1.20	27.48	0.02	ENSRNOT0000 0053106	10908229	
	1.20	14.90	0.02	ENSRNOT0000 0042423	10938712	
	1.21	22.9	0.032	ENSRNOT0000 0058170	10823117	
	1.23	32.0	0.024	ENSRNOT0000 0057159	10766878	
	1.24	17.1	0.015	ENSRNOT0000 0054610	10885765	
	1.25	15.4	0.000	BC168168	10856610	
	1.25	14.1	0.015	ENSRNOT0000 0060154	10771949	
	1.25	40.5	0.013	18999385, 14043292	10727640	
	1.27	12.6	0.007	rno-mir-10b	10837017	
	1.30	61.1	0.037	ENSRNOT0000 0060468	10801258	
	1.31	62.1	0.019	ENSRNOT0000 0050135	10837097	
	1.32	31.2	0.017	rno-mir-224	10935829	
	1.35	35.7	0.030	ENSRNOT0000 0052428	10909375	
